# Structural and functional insights into calmodulin-mediated lipid binding and proteolytic cleavage of the M-PMV matrix protein

**DOI:** 10.1016/j.jbc.2025.111102

**Published:** 2025-12-23

**Authors:** Karolina Buresova, Tereza Nesporova, Jan Prchal, Swati Banerjee, Marketa Castoralova, Lucie Hodbodova, Zdenek Kukacka, Petra Junkova, Tomas Ruml

**Affiliations:** 1 Department of Biochemistry and Microbiology, University of Chemistry and Technology, Prague, Czech Republic; 2 Institute of Organic Chemistry and Biochemistry, The Czech Academy of Science, Prague, Czech Republic; 3 Department of Analytical Chemistry, Faculty of Science, Charles University, Prague, Czech Republic; 4 Institute of Microbiology, The Czech Academy of Sciences, Prague, Czech Republic

**Keywords:** calmodulin, retrovirus, matrix protein, structural proteomics

## Abstract

The matrix (MA) domain of the Mason-Pfizer monkey virus (M-PMV) Gag polyprotein plays a central role in retroviral assembly and trafficking, coordinating membrane association and proteolytic maturation. Unlike HIV-1, M-PMV assembles immature particles in the cytoplasm prior to plasma membrane targeting, but the molecular mechanisms governing this process remain poorly understood. Here, we identify calmodulin (CaM) as a calcium-dependent modulator of MA structural dynamics. Using a combination of biophysical and biochemical methods, we demonstrate that CaM directly interacts with myristoylated MA, promoting its oligomerization and enhancing its cleavage by the viral protease. In-depth characterization of MA-CaM complex by protein cross-linking mass spectrometry, hydrogen/deuterium exchange mass spectrometry and NMR spectroscopy reveals that the N-terminal parts of both proteins are in close proximity within the complex and that CaM binding induces increased conformational flexibility of key regions within MA, including the basic patch and C-terminal cleavage site. These dynamic changes suggest an allosteric mechanism by which CaM regulates MA function, potentially facilitating the temporal coordination of membrane targeting, the myristoyl switch and proteolytic processing. Our findings broaden the understanding of CaM as a regulatory factor in retroviral assembly and underscore the importance of conformational plasticity in viral maturation.

Viruses rely on host cellular calcium signaling pathways to support critical stages of their life cycle, such as entry, replication, assembly, and release. They manipulate calcium signaling through three main strategies: disrupting intracellular Ca^2+^ homeostasis through modulating calcium channels, pumps, or membrane permeability; utilizing Ca^2+^-regulated proteins and pathways to control their life cycle; and directly binding Ca^2+^ to hijack host cell functions and compromise cellular integrity; as comprehensively summarized by (1, 2).

One of the most studied Ca^2+^-regulated proteins is calmodulin (CaM), a ubiquitous cellular calcium sensor that undergoes a conformational transition from a closed to an open state upon Ca^2+^ binding, exposing its helix-loop-helix motifs and hydrophobic binding domains. This structural rearrangement enables CaM to interact with over 300 target cellular proteins, including key enzymes such as kinases and phosphatases, ion channels, and transcription factors, thereby orchestrating diverse cellular processes (3, 4). CaM plays a critical role in regulating ion homeostasis by controlling Ca^2+^-ATPases to restore cytosolic calcium levels (5). It also interacts with transcription factors such as CAMTA3 to induce stress-responsive gene expression and modulates kinase and phosphatase activities that are essential for salt tolerance and reactive oxygen species balance (6, 7). Under abiotic stress conditions like salinity, cold, drought, and osmotic stress, as well as during biotic stress such as viral infection, CaM expression and activity are modulated to coordinate adaptive responses (8). Stress conditions, including viral attack, can induce post-translational modifications and subcellular relocation of CaM, fine-tuning its interactions with target proteins to enhance cellular resilience (9). Through these mechanisms, CaM acts as a dynamic integrator of calcium signals, enabling cells to adapt efficiently to environmental and pathogenic challenges and maintain homeostasis, particularly under stress conditions.

CaM expression and activity are modulated upon viral infections as shown for several phylogenetically distant viruses, including rotavirus, Ebola virus, gemini virus, and retroviruses (10, 11, 12). For example, infection by porcine epidemic diarrhea virus significantly increases the accumulation of calmodulin-like 5 protein which promotes viral internalization and virion release by regulating late endosome synthesis and modulating the innate immune response, including suppression of interferon- β (13). Epstein–Barr virus infection induces expression of Ca^2+^/calmodulin-dependent kinase in transformed B lymphocytes, indicating modulation of CaM-dependent signaling pathways and B-lymphocyte growth transformation mediated by viral infection (14). CaM also plays a calcium-dependent role in the budding of Ebola virus-like particles and recombinant Ebola virus, indicating that CaM is an important factor during Ebola virus infection (11). In addition, the VP1 protein of enterovirus activates calmodulin-dependent protein kinase II, which phosphorylates vimentin. Its phosphorylation-driven rearrangement may promote viral replication by playing a structural role in the formation of replication factories (15). It was also shown that a direct interaction of Ca^2+^-stimulated CaM with rotavirus protein VP6 positively regulates rotavirus propagation, further illustrating the role of CaM in viral life cycles (16).

In retroviruses, the presence of CaM in isolated viral particles was initially described for Feline leukemia virus (FLV), murine leukemia virus (MuLV), and Human T-lymphotropic virus (HTLV) (17). Afterward, several HIV-1 proteins were shown to bind to CaM in Ca^2+^-dependent manner. For example, amphipathic helix of cytoplasmic tail of HIV-1 envelope glycoprotein (gp160) was shown to bind CaM (18). Additionally, expression of full-length HIV-1 gp160 was shown to increase the amount of cellular CaM in comparison to the expression of its C-terminally truncated form. Finally, colocalization of gp160 and CaM was confirmed by confocal microscopy (19). Similarly, the interaction of simian immunodeficiency virus (SIV) transmembrane glycoprotein 41 and CaM was also described (20). The interaction of another HIV-1 protein, the Nef protein, with CaM was confirmed in both, *in vivo* and *in vitro* (21). Also, HIV-1 structural polyprotein Gag was shown to interact with CaM through its N-terminal domain; Matrix protein (MA) and similarly as in the case of Nef, Gag colocalized with CaM when examined by confocal microscopy (22). Although Radding *et al*. (22) did not observe the direct interaction between CaM and HIV-1 capsid protein (CA) domain of Gag in his work, this interaction was confirmed by NMR spectroscopy in the later work (23).

Structural aspects of the interaction between HIV-1 MA and CaM were determined by NMR and far-UV circular dichroism (CD) experiments showing that HIV-1 MA binds to CaM in a calcium-dependent manner, with a 1:1 stoichiometry. Additionally, these experiments revealed that HIV-1 MA undergoes significant conformational changes due to this interaction (24). Soon afterwards, these findings were expanded by Ghanam *et al*. (25) who determined the K_D_ of this interaction by isothermal titration calorimetry (ITC) to ∼ 2 μM. Additionally, Ghanam *et al*. (25) in their work confirmed that interaction between HIV-1 MA and CaM triggers the exposition of HIV-1 MA myristoyl, *i*.*e*., the mechanism known as myristoyl switch. Interestingly, the interaction between HIV-1 MA and CaM is not facilitated by myr moiety itself as it was described in case of Nef protein (21) but by the residues 8 to 43 as was later described by using NMR spectroscopy (26, 27). Structural aspects of the interaction were further studied by small-angle scattering technique, which described that dramatical conformational changes of HIV-1 MA caused by its interaction with CaM are reversibly dependent on Ca^2+^ or increasing ionic strength (28).

Although interactions between CaM and several retroviral proteins have been described, the direct impact of this interaction for retroviral life cycle remains to be elucidated. To bring further insights to this field, we focused on the previously uncharacterized interaction between Mason-Pfizer monkey virus (M-PMV) MA and CaM, as another representative of retroviruses. The results presented here reveal a direct interaction between M-PMV MA and CaM *in vitro*, as well as confirm the interaction between M-PMV structural polyprotein Gag and CaM in host cells. We also revealed the positive impact of CaM on M-PMV maturation through its interaction with M-PMV MA and its possible role in myristoyl switch of M-PMV MA, which has not been experimentally observed yet. Finally, we described the structural aspects of M-PMV MA-CaM interaction.

## Results

### Interaction of MA M-PMV with calmodulin *in vitro*

**Figure 1:**
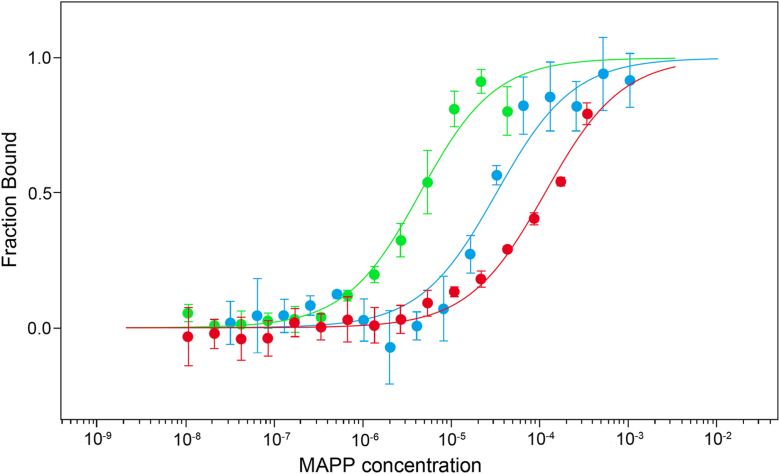
**Fraction bound plot representing interaction of myrMAPP and MAPP with calmodulin**. Binding of fluorescently labeled CaM to myrMAPP (*green*) and non-myristoylated MAPP (*blue*) in the presence of 20 mM Ca^2+^ and to myrMAPP in the presence of EDTA (*red*), as determined by MST. Error bars represent standard deviations of 3 technical replicates.

Microscale thermophoresis (MST) was used to analyze the binding of M-PMV MA to fluorescently labelled CaM (CaM-NHS-Red). Both proteins were produced as described in the Methods section. CaM was fluorescently labeled using Protein Labeling Kit Red-NHS second Generation (Nanotemper Technologies); the efficiency of labeling was 0.82. During the MST measurements, the concentration of fluorescently labelled CaM was kept constant at 466 nM, while the concentration of myrMAPP (myristoylated MA protein extended by 18 amino acids-long downstream phosphoprotein region of Gag) or MAPP (non-myristoylated version of the myrMAPP) ranged from 16 nM to 1050 μM. All experiments were measured in triplicates. The interaction between M-PMV myrMAPP and CaM was shown to be calcium dependent. The K_D_ for this interaction in the presence of calcium ions was determined as 4.2 μM, suggesting specific interaction. In contrast, removal of calcium ions by EDTA markedly reduced binding affinity, resulting in K_D_ of 115 μM, corresponding to very weak or nonspecific interaction. MAPP showed about 10-fold higher K_D_ of 33 μM in presence of calcium than myrMAPP (Fig. 1). These K_D_ values are similar to those described for interaction of HIV-1 MA with CaM (K_D_ = 2 μM) (25).

The interaction between M-PMV MA and CaM was further inspected by protein cross-linking with the use of two cross-linkers with different lengths, *i*.*e*. DSBU and DSPU with spacer arms of 12.5 Å and 10.1 Å, respectively (29). Samples containing myrMAPP with CaM in presence of calcium ions, as well as samples of the individual proteins, were subjected to cross-linking using 2 M excesses of cross-linker relative to protein (5-fold molar excess and 10-fold molar excess). Since cross-linking agents covalently link protein residues in close proximity, protein complexes are maintained under denaturing conditions and subsequently visualized by SDS-PAGE using Coomassie blue staining or immunoblotting.

**Figure 2:**
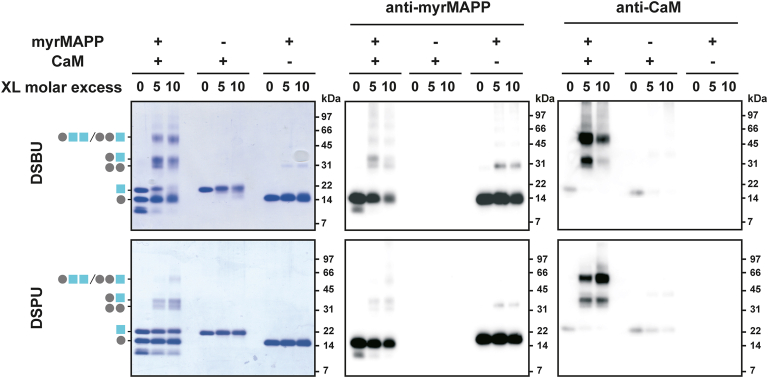
**SDS-PAGE and immunoblot analysis of cross-linked MA-CaM complexes**. The mixture of CaM and myrMAPP as well as individual proteins were treated with DSBU and DSPU cross-linkers at increasing molar excesses of cross-linker to protein (5-fold molar excess and 10-fold molar excess) and analyzed by SDS-PAGE with Coomassie detection (*left panels*) or with immunoblot detection by the use of antibodies against M-PMV MA (*middle panels*) or against CaM (*right panels*). Individual proteins were detected as signals of ∼14.4 kDa (myrMAPP; illustrated by a *gray circle*) and ∼17 kDa (CaM; illustrated by a blue square) which corresponds to their molecular weight (14.9 kDa of myrMAPP and 16.7 kDa of CaM). In mixed samples of myrMAPP and CaM, additional bands of higher molecular weight appear upon cross-linker treatment, indicating formation of oligomeric CaM–myrMAPP complexes. Data shown are representative of two independent biological replicates.

Coomassie Brilliant Blue-stained SDS-PAGE gels (Fig. 2; left panels) showed bands for CaM and myrMAPP at their expected molecular weights (∼17 kDa and ∼14.4 kDa, respectively), with no major shifts in the absence of the cross-linker. Conversely, multiple intense signals of protein complexes were detected in the lanes corresponding to mixed samples of myrMAPP and CaM treated with each cross-linker. This suggests the presence of complexes formed between CaM and myrMAPP. However, the detected signals corresponded not only to the myrMAPP-CAM complex (∼34 kDa), but also to myrMAPP dimer (∼31 kDa) and higher-order complexes. Although the myrMAPP dimer signal was also present in the sample containing only M-PMV myrMAPP, its intensity was significantly reduced compared to the corresponding signal in the mixed sample.

Immunoblot analysis of cross-linked proteins using the antibodies against either M-PMV (myr)MA (Fig. 2; middle panels) or CaM (Fig. 2; right panels) confirmed the formation of myrMAPP-CaM heterodimer, as evidenced by the expected signal (∼34 kDa) present in lines corresponding to the samples containing both proteins, but absent in those with only individual proteins. In addition, both myrMAPP and CaM provided signals corresponding to multiples of their molecular weights. Interestingly, we consistently observed a significant increase of CaM signal in immunoblots upon CaM cross-linking, probably due to the preservation of the native CaM structure recognized by the antibody, which is lost under denaturing conditions in samples without cross-linking.

**Figure 3:**
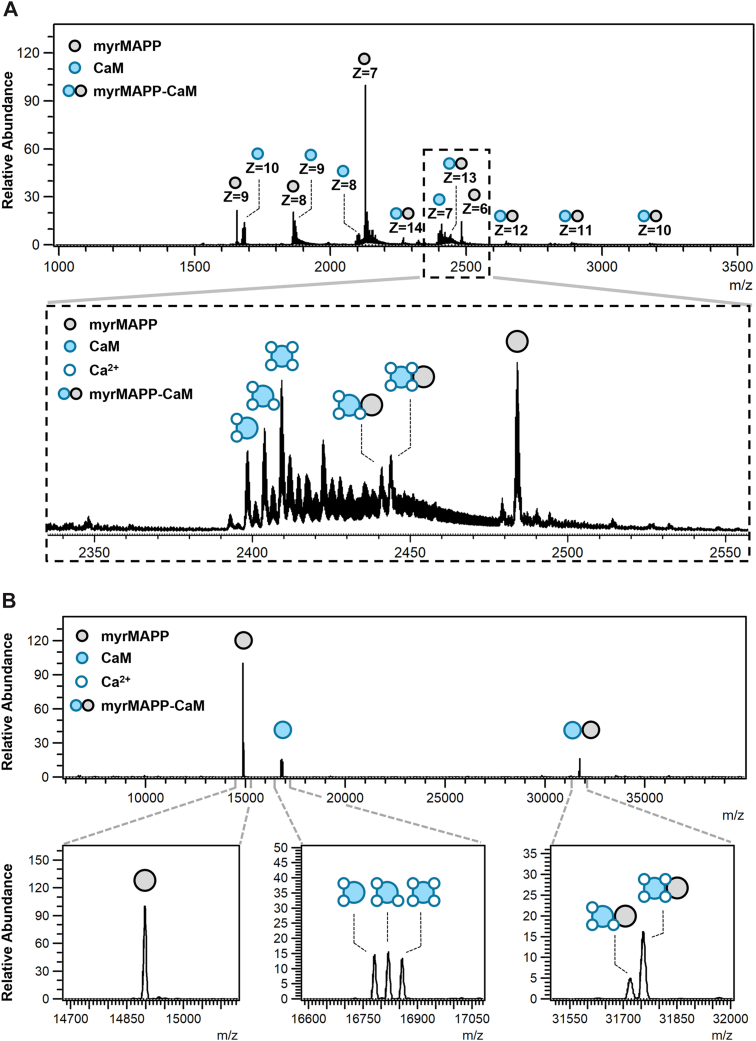
**Native mass spectrum of myrMAPP-CaM mixture**. *A*, Full scan raw mass spectrum showing the distribution of individual charge states of myrMAPP, CaM and their complexes. Enlarged m/z region shows the signals corresponding to monomeric protein states with charge state of 6 (myrMAPP) and 7 (CaM) and protein complexes with charge state of 13 detected in the presence of 0.5 mM calcium acetate. *B*, spectrum showing deconvoluted signals of monomeric protein states and protein complexes detected in the presence of 0.5 mM calcium acetate. Enlarged m/z regions show individual forms of detected proteins and complexes. MA was detected as a unique signal of 14,897 Da (*lower left panel*) while CaM was detected in three forms (*lower middle panel*), *i*.*e*. form binding two ions of calcium (16,782 Da), three ions of calcium (16,820 Da) and four ions of calcium (16,858 Da). The complex of myrMAPP with CaM was detected in two forms (*lower right panel*), *i*.*e*. form in which CaM binds three ions of calcium (signal of 31,719 Da) and four ions of calcium (signal of 31,756 Da).

Since MST and protein cross-linking provided evidence that myrMAPP interacts with CaM, native mass spectrometry (native MS) analysis was further employed. Native MS uses soft ionization techniques with very gentle conditions that preserve native, folded state of proteins and their non-covalent interactions. In mass spectra analyzed ions have a narrow charge state distribution compared to standard non-native protein analyzes (30, 31). We used this method to detect the signals confirming the presence of myrMAPP-CaM complex and to determine the stoichiometry of complexes detected in cross-linked samples by SDS-PAGE (Fig. 2). The analysis performed in 150 mM ammonium acetate in the presence of 0.5 mM calcium acetate (Fig. 3) confirmed that myrMAPP forms complex with CaM. Signals of the complex were detected in multiple charge states (z = 10–14; Fig. 3*A*) and after deconvolution of spectra it was represented by two distinct signals (Figs. 3*B*, S1). Similarly to the results of SDS-PAGE analysis of cross-linked proteins (Fig. 2), the signals of monomeric proteins were more abundant also in mass spectra.

Further native MS analysis of the controls such as individual myrMAPP or CaM in the presence and in the absence of calcium ions as well as myrMAPP and CaM mixture in the absence of calcium ions were analyzed (Fig. S1) showed the presence of exclusively monomeric forms of proteins. This confirmed the outcomes of MST that the formation of myrMAPP-CaM complex is calcium dependent. Additionally, in the presence of 0.5 mM calcium acetate, three stoichiometric forms of CaM, each binding two, three, or four calcium ions, were detected (Fig. 3*B*; bottom middle panel). Contrary to the expectations that calmodulin must bind four calcium ions to adopt a conformation suitable for interaction with target proteins, we found that the myrMAPP-CaM complex was formed even when calmodulin binds only three calcium ions. However, CaM binding four calcium ions was preferred for the formation of the complex, since the signal corresponding to MA-CAM complex with four bound calcium ions was more intensive than those with three ions bound (Fig. 3*B*; bottom right panel).

### Intracellular interaction of structural polyprotein Gag of M-PMV with CaM

**Figure 4:**
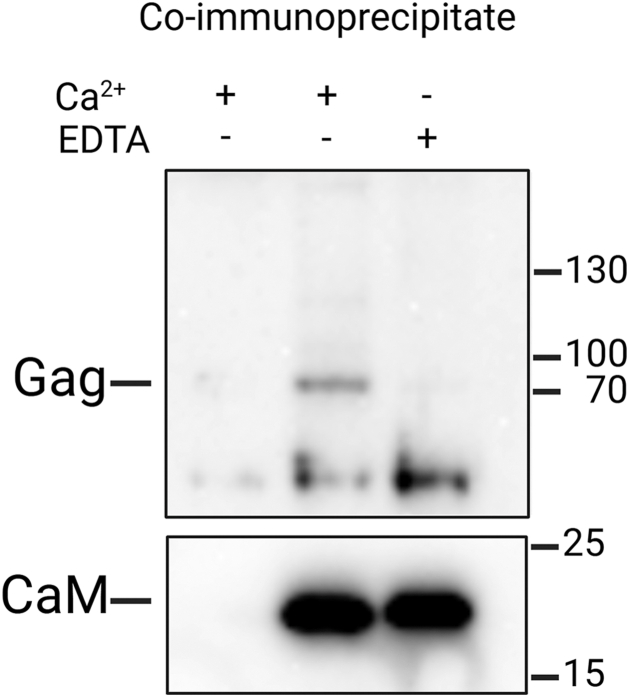
**Co-immunoprecipitation of M-PMV Gag and HA-tagged CaM in HEK293T cells**. HEK293T cells were co-transfected with plasmids encoding non-infectious M-PMV (pSARM4 RT) and HA-tagged human CaM (pCMV HA-CaM). Cell lysates were subjected to co-immunoprecipitation using anti-HA magnetic agarose beads (Chromotek HA-trap). Anti-MA antibody (1:2500) was used for immunoblot detection. The binding of HA-CaM was inspected by immunoblot using anti CaM antibody. Note, the bottom band is a nonspecific signal that was also detected in the mock sample of non-transfected cells (see Fig. S2). Data shown are representative of three independent biological replicates.

Co-immunoprecipitation using an anti-HA nanobody system was employed to assess the formation of intracellular complexes between the MA domain of M-PMV Gag and CaM. HEK293 T cells were co-transfected with pCMV HA-CaM vector for production of HA-tagged human CaM and pSARM4 RT vector for production of assembly and maturation-competent, but non-infectious M-PMV particles with catalytically inactive reverse transcriptase. It is worth noting that Gag produced in this way is N-terminally myristoylated, *i*.*e*., it contains a myristoylated MA domain. Cells were lysed 48 h post-transfection and proteins were co-immunoprecipitated using Chromotek HA-trap magnetic agarose in the presence of Ca^2+^ ions with or without EDTA. As a negative control, lysate of cells co-transfected by pSARM4 RT and empty pCMV HA was analyzed. As shown in Figure 4, co-immunoprecipitation of Gag protein predominantly occurred in the presence of Ca^2+^ (middle lane), while only barely detectable signal was observed when Ca^2+^ ions were depleted by EDTA-mediated chelation (right lane). This confirms the calcium-dependent nature of the interaction between CaM and M-PMV Gag polyprotein. The negative control (left lane) showed no detectable signal of co-immunoprecipitated Gag, confirming the specificity of the interaction between M-PMV Gag and HA-CaM. Additionally, comparable Gag expression across the tested samples was confirmed by immunoblot analysis of input samples (Fig. S2).

### Regulation of M-PMV MA protein function by calmodulin: Implications for myristoyl switch and proteolytic processing

**Figure 5:**
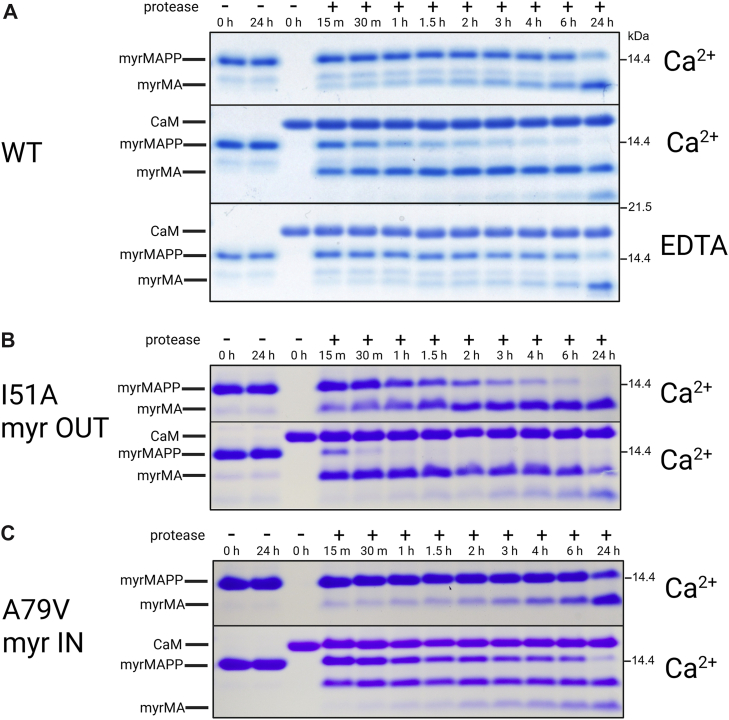
**Proteolytic cleavage of myrMAPP variants by viral protease in the presence or absence of CaM**. SDS-PAGE analysis was used to determine the time-dependent course of proteolytic cleavage of myrMAPP WT (*A*) and its mutant variants (*B*–*C*). The dependence of myrMAPP WT proteolytic cleavage on the presence of CaM and calcium ionts is shown in *panel* (*A*). The I51A mutant (*B*), representing the myr OUT state, reduces the hydrophobicity of the protein core. The A79V mutant (*C*), representing the myr IN state, increases core hydrophobicity and impairs the myristoyl switch. Each variant was analyzed either in the absence (*top panels*) or presence (bottom panels) of calmodulin (CaM). Data shown are representative of three independent biological replicates.

Subsequent experiments focused on determining the consequences of the interaction between M-PMV MA and CaM. Binding of CaM to HIV-1 MA has been shown to trigger the myristoyl switch in HIV-1. A possible way to indirectly demonstrate the occurrence of the myristoyl switch in M-PMV MA is based on our previous observation that the myristoyl switch induces conformational changes in M-PMV MA that expose the C-terminal cleavage site. This enables efficient proteolytic separation of MA from the rest of the Gag polyprotein (32). Therefore, to investigate whether the presence of CaM influences the cleavage of myrMAPP, we performed *in vitro* cleavage assays. As shown in Figure 5*A*, the presence of CaM and calcium ions in the reaction mixture significantly accelerated the appearance of cleavage products derived from the myrMAPP precursor. In the presence of both CaM and calcium ions, the viral protease cleaved the majority of myrMAPP into myrMA within 1 h. In contrast, in the absence of CaM or in its presence but in condition depleting calcium ions (EDTA), the signal of uncleaved myrMAPP remained detectable even after 24 h of incubation.

To further confirm the effect of CaM on the conformation of MA and myristoyl exposure, we compared the efficiency of proteolytic cleavage of the wild-type myrMAPP with that of myrMAPP mutants in which the myristoyl switch is either facilitated or hindered. As described in a previous study (32), the I51 A (myr OUT) mutation, which reduces the hydrophobicity of the protein core and promotes the myristoyl switch, also enhances the cleavage of myrMAPP to MA. In contrast, the A79 V (myr IN) mutation, which increases core hydrophobicity and impairs the myristoyl switch, reduces the speed of cleavage. Interestingly, the interaction of CaM with myrMAPP enhanced proteolytic cleavage not only of WT but also of both mutants (Fig. 5, *B* and *C*). The myrMAPP I51 A mutant was completely cleaved in less than 1 hour, and also the cleavage of myrMAPP A79 V, which hinders the myristoyl switch, was significantly enhanced in the presence of CaM. According to our hypothesis, the interaction of CaM, in presence of calcium ions, with the MA domain promotes the exposure of the myristoyl group from the core of myrMAPP even in the mutant with an otherwise impaired myristoyl switch and concurrently induces a conformational change that renders the C-terminal cleavage site accessible to viral protease.

**Figure 6:**
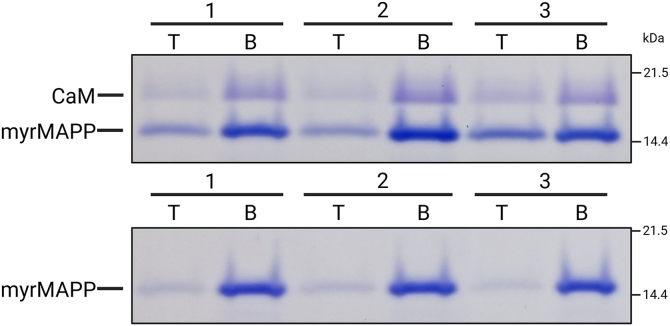
**Liposome binding assay of myrMAPP in the presence or absence of CaM**. MyrMAPP protein was incubated with liposomes mimicking the phospholipid composition of the plasma membrane in the presence or absence of CaM and the reaction mixture was subjected to ultracentrifugation in two-layer discontinuous sucrose gradient. Fractions from the top (T) of fraction containing liposomes and proteins bound to them and the bottom (B) fraction containing unbound proteins were analyzed by SDS-PAGE in three technical replicates (indicated 1–3). Data shown are representative of two independent biological replicates.

Since myristoyl exposure facilitates the interaction of MA with the membrane, we used flotation liposome binding assay to assess whether CaM affects myrMAPP binding efficiency to liposomes mimicking the cytoplasmic membrane (33). As shown in Figure 6 (upper panel), in the presence of CaM and liposomes, the myrMAPP was enriched in the top fractions containing liposomes compared to myrMAPP samples lacking CaM (Fig. 6; lower panel), where the myrMAPP exhibited significantly weaker liposome association. Both samples contained calcium ions. These results indicate that CaM promotes the association of myrMAPP with liposomes, likely by triggering the myristoyl switch, thereby enabling the viral protein to bind the plasma membrane. Control samples of liposome binding assay are presented in Figure S3.

### Structural analysis of MA-CAM complex

To further characterize the interaction between myrMAPP and CaM in presence of calcium ions, we used structural proteomics to describe the protein interaction interface in the complex. Specifically, we applied protein cross-linking with mass spectrometric detection (XL-MS), NMR spectroscopy, and hydrogen-deuterium exchange with mass spectrometry detection (HDX-MS).

To map the distances between individual myrMAPP and CaM residues, we performed XL-MS analysis. This allowed us to identify the residues of myrMAPP and CaM, which occur in the MA-CaM complex at a close proximity given by the length of the cross-linker. In these experiments, we used the same cross-linkers as in the initial cross-linking-based electrophoretic analysis of the complexes, *i*.*e*., DSBU and DSPU, which differ in the length of spacer arms (29). Both cross-linkers covalently bind predominantly lysine residues, but the reaction with serine, tyrosine or threonine residues is also possible (34). The myrMAPP and CaM equimolar mixtures were cross-linked and analyzed in three replicates. Additionally, a sample of myrMAPP was cross-linked by DSBU in the same way as the mixture of myrMAPP and CaM to determine the differences between cross-linked myrMAPP residues in the presence and absence of CaM. Finally, the same comparison was performed by the use of the equimolar mixture of isotopically labelled and non-labelled myrMAPP to determine inter-molecular linkages of myrMAPP molecules formed in the presence and in the absence of CaM.

**Figure 7:**
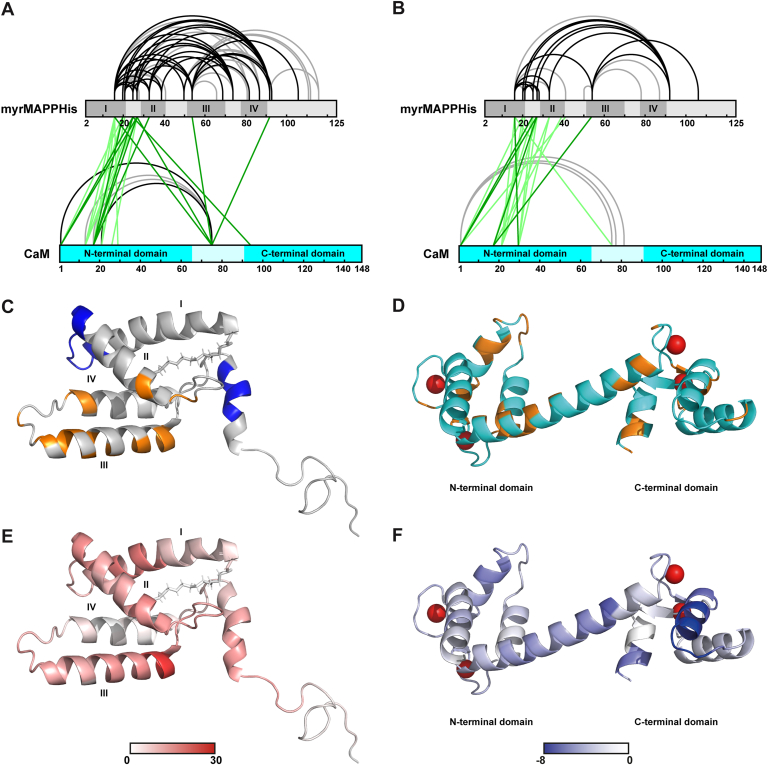
**Structural features of the MA-CaM complex**. *A*, visualization of DSBU cross-links identified in myrMAPP-CaM mixed sample. *B*, visualization of DSPU cross-links identified in myrMAPP-CaM mixed sample. Unique myrMAPP-CaM linkages identified in all three technical replicates are in *dark green*; other identified unique myrMAPP-CaM linkages are in *light green*. Unique myrMAPP-myrMAPP or CaM-CaM linkages identified in all three technical replicates are in *black*, other identified unique myrMAPP-myrMAPP or CaM-CaM linkages are colored *gray*. Darker shades of protein sequence bars show the positions of individual helices in the structure of myrMAPP or N- and C-terminal domains of CaM. Diagrams were created by xiVIEW visualization tool (69). *C*, changes identified by NMR analysis mapped onto the structure of myrMAPP (PDB entry 5lmy). Residues with chemical shifts in ^15^N-labeled myrMAPP upon equimolar addition of unlabeled CaM are colored in *orange*, residues in ^15^N-labeled myrMAPP that exhibited NMR signals line broadening induced by the presence of increased concentration of CaM (1:5) are shown in *blue*. *D*, Chemical shifts detected by NMR analysis visualized within the structure of CaM (PDB entry 1Cll). Residues with chemical shifts detected for ^15^N-labeled CaM upon equimolar addition of unlabeled myrMAPP are colored *orange*. *E*, HDX relative fractional uptake differences of myrMAPP in mixed myrMAPP-CaM sample compared to the individual myrMAPP visualized in the structure of myrMAPP (PDB entry 5lmy) for time point of 20 s. *F*, HDX relative fractional uptake differences of CaM in mixed myrMAPP-CaM sample compared to individual CaM visualized in the structure of CaM (PDB entry 1Cll) for time point of 20 s.

In total, 21 different linked pairs of myrMAPP-CaM residues were identified by DSBU cross-linking, 11 of which were identified in all three replicates (Fig. 7*A*, Table S1). DSPU cross-linking led to the identification of 18 linked myrMAPP-CaM residue pairs, 5 of which were identified in all three replicates (Fig. 7*B*, Table S2). The lower number of linkages obtained using DSPU compared to DSBU corresponds to its shorter arm. These results not only confirmed that both proteins form a complex but also helped to reveal that N-terminal portions of the two proteins are in close proximity, as most of the identified myrMAPP-CaM linkages were located between residues located in the N-terminal parts of both proteins. In the case of the longer DSBU cross-linker, several linkages connecting the residues from the middle part of the CaM chain and residues from different parts of the myrMAPP chain were consistently detected. These linkages were not detected by the use of DSPU, which indicates that these residues occur at a greater distance than the maximal distance restraints of the DSPU cross-linker. The spectra of all detected MA-CaM linkages are shown in Figure S4. In case of linkages between different myrMAPP residues identified in mixtures of myrMAPP and CaM, a total of 50 linkages were identified using DSBU, with 29 detected in all three replicates. In contrast, only 21 cross-links in myrMAPP were identified using DSPU, with 13 consistently observed across all three replicates.

Since myrMAPP behaves differently in the presence of CaM than as a single protein, as we presented previously (Fig. 5), we hypothesize that the interaction of MA with CaM induces some changes in MA conformation. Therefore, we also performed cross-linking of the sample of individual myrMAPP and compared obtained myrMAPP cross-links in two states, *i*.*e*. in the presence and absence of CaM. For this comparison, DSBU as a longer cross-linker was used to reveal the major changes in myrMAPP structure upon interaction with CaM. Indeed, we identified several myrMAPP intra-links, which were present in all three replicates of one state and absent in the replicates of the second state (Fig. S5). The most notable differences in myrMAPP cross-links identified in the presence and absence of CaM involved connections between N-terminal residues and C-terminal residues (K16-T116 and K39-K111) (Fig. S5). Both linkages were consistently observed when myrMAPP was analyzed alone and were not found in the presence of CaM. The loss of these linkages in the presence of CaM points to additional change in myrMAPP conformation caused by its interaction with CaM resulting in the spatial separation of N- and C-terminal myrMAPP regions. As shown previously in Figure 2, myrMAPP exhibits the tendency to form oligomeric complexes in the presence of CaM. To verify this, we again used XL-MS. Although the XL-MS provides valuable information about the distance restraints of targeted amino acid residues in protein complexes, the analysis of homo-oligomeric complexes is seriously challenging because the standard approach cannot distinguish between intra- and inter-links. To overcome this problem, we used a mixed-isotope labeling approach coupled with XL-MS. An equimolar mixture of uniformly ^15^N-labeled and unlabeled myrMAPP was cross-linked in the presence and absence of CaM and calcium ions. Four inter-links (K16-K25, K16-K33, K25-K39, K27-K54; Fig. S6) connecting labeled and unlabeled MA peptides were identified. Additionally, these inter-links were identified only in the presence of CaM and no inter-link was identified in the absence of CaM (whole dataset obtained by mixed-isotope labeling of MA and MA-CAM samples showing detected intra- and inter-links is deposited in the database of the ProteomeXchange Consortium *via* the PRIDE under dataset identifier PXD067304). This is consistent with the data shown in (Fig. 2) which indicate that the presence of CaM enables oligomerization of myrMAPP and aligns well with our previous findings that myrMAPP does not significantly oligomerize *in vitro* (35). Our mixed-isotope labeling XL-MS analysis also showed that the N-terminal regions of the myrMAPP molecules come into closer proximity during myrMAPP oligomerization.

In the NMR spectroscopy experiments, both CaM and myrMAPP were uniformly ^15^N-labeled and studied in the presence of their respective unlabeled binding partners, as well as calcium ions. Interaction with the unlabeled partner was detected through changes in the chemical shifts of hydrogen and nitrogen atoms in NH groups, primarily from the protein backbone. Since these chemical shifts are highly sensitive to their local environment, they serve as reliable indicators of binding events. Additionally, line broadening of NMR signals in the presence of the interaction partner can indicate a slower exchange regime, changes in local dynamics, or the formation of larger complexes. Chemical shifts of both myrMAPP and CaM were obtained from previously published assignments (35, 36). Spectra of labelled myrMAPP with equimolar addition of unlabeled CaM showed significant changes in chemical shifts of backbone NH groups of amino acid residues F45, I53, K54, R55, Q64, D68, F70, T78 and A79 (Fig. 7*C*, S7*A*). Additionally, less intensive changes in chemical shifts of residues K27, D40, W44, R57, R58, D61, D65, Y67 and E73 were also observed. At an increased concentration of CaM, reaching a myrMAPP:CaM ratio of 1:5, we additionally observed line broadening of residues in the regions spanning 19 to 29 and 98 to 103 (Fig. S7*B*). NMR spectra of labelled CaM in the presence of equimolar concentration of unlabeled myrMAPP showed intensive signal broadening and/or large chemical shift changes of backbone NH groups of amino acid residues F12, G25, I27, T28, T29, G33, B35, M36, S38, G40, Q41, N42, I52, N53, E54, V55, I63, F65, F68, M71, M72, R74, K75, M76, T79, A88, V91, G96, I100, A102, R106, L116, T117, D129, I130, D131, Q143, M145, A147 and K148 (Fig. 7*D*, S7*C*). The spectrum of CaM in the presence of myrMAPP shows more pronounced changes than those observed for myrMAPP upon CaM binding, suggesting a pronounced conformational change of CaM upon complex formation.Using hydrogen–deuterium exchange (HDX)-mass spectrometry (MS), we conducted a detailed mapping of the interaction interface between myrMAPP M-PMV and CaM in presence of calcium ions. The hydrogen exchange rates of backbone amides reflecting their surface exposure and accessibility were measured in triplicates at 4 °C over a time range from 5 s to 2 h. This approach allowed us to capture both the rapid HDX of flexible loops and the slower HDX occurring in the more rigid helices. For both proteins, exceptionally high sequence coverage (>99%) was achieved, with substantial redundancy in individual amino acids (5.7 for myrMAPP, 15.6 for CaM), ensuring a comprehensive structural analysis of their interaction dynamics. To facilitate complex formation, the proteins were mixed at a 1:4 M ratio (myrMAPP:CaM), ensuring at least 50% of myrMAPP and at least 13% of calmodulin should be incorporated into the myrMAPP-CaM complex.

Statistically significant HDX differences between individual myrMAPP and myrMAPP in the myrMAPP-CaM complex (1-p threshold = 0.95) were observed across all five time points, mostly in regions Y11-V24 and K93-Q105 (Fig. 7*E*, S8). However, a notable increase in HDX levels was detected in multiple regions throughout the entire sequence of myrMAPP. Higher HDX levels of myrMAPP peptides in myrMAPP-CaM complex were also detected in regions of I., II. and III. helices and the loops around them (Q3-R10, K28-L32, K39-I53, R58-Q64, K74-S81 regions). Interestingly, EX1 deuteration kinetics were detected in peptides spanning the regions K39–I53, R58–Q64, and especially Y11–K27 (Fig. S9*A*). Such kinetics typically indicate higher-order motions, including helix fraying, β-sheet peeling, or domain unfolding. Statistically significant HDX differences between individual CaM and CaM in myrMAPP-CaM complex (1-p threshold = 0.95) were also observed at all time points (Fig. 7*F*, S9). The regions of C-terminal globular domain (A102-L115) exhibit lower HDX levels compared to N-terminal globular domain (S18-M37) at initial timepoints (5 s, 20 s) and after 2 h of deuteration, a significant decrease in HDX was observed in multiple regions throughout the entire sequence of CaM in myrMAPP-CaM complex. Representative deuteration kinetics plots from the regions of interest are available in the supplementary data (Fig. S10). HDX thus provided insights into changes in the solvent accessibility of various regions in both proteins upon their interaction. In summary, myrMAPP appears to exhibit increased flexibility across its entire sequence in the presence of CaM, whereas several regions of CaM show reduced flexibility when bound to myrMAPP. These observations suggest that the interaction with CaM induces conformational changes in myrMAPP.

**Figure 8:**
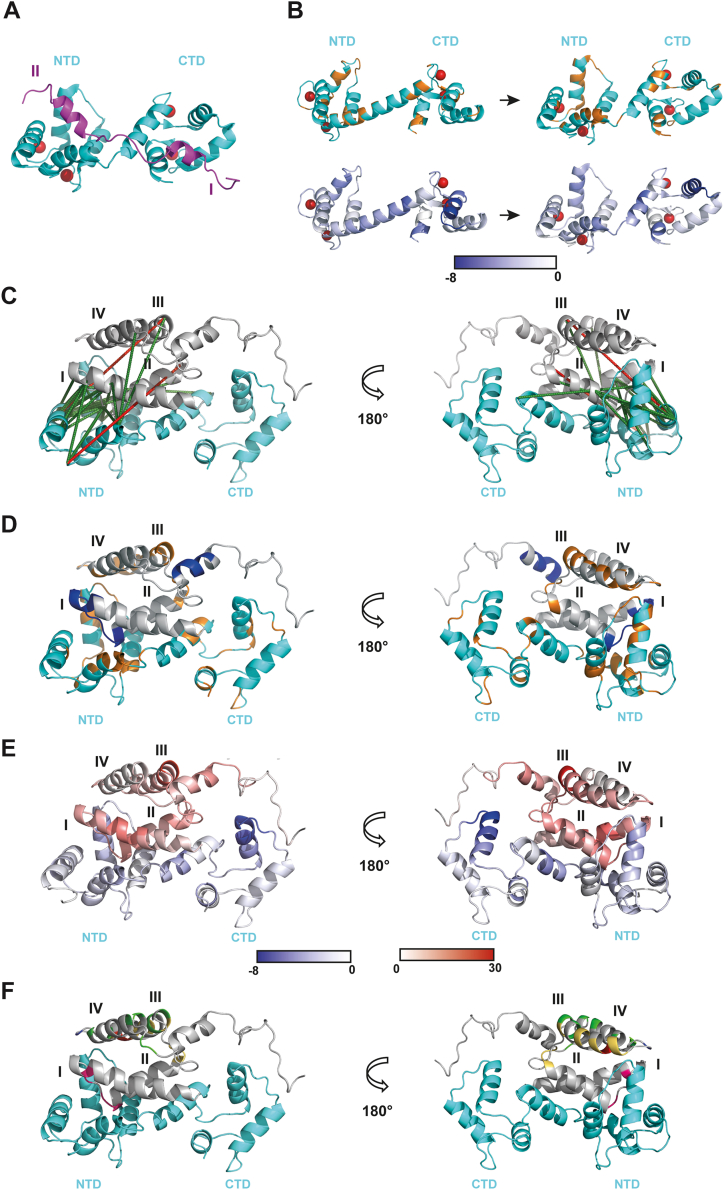
**Model of myrMAPP-CaM complex**. *A*, structure of HIV-1 MA(8–43)-CaM complex published by Vlach *et al*. (PDB entry 2mgu) (26). CaM is colored in *cyan*, HIV-1 MA(8–43) in *magenta* and calcium ions in *red*. NTD – N-terminal domain, CTD – C-terminal domain. *B*, NMR chemical shift changes and differential HDX relative fractional uptake detected for CaM interacting with M-PMV myrMAPP mapped onto the published structure of free CaM and CaM in complex with HIV-1 MA(8–43) (26). Free CaM (PDB 1Cll) is shown on the *left*, and CaM, as observed in the complex with HIV-1 MA(8–43) on the *right*, both colored according to our M-PMV myrMAPP interaction results. *Top panels*: NMR chemical shifts, with residues showing shifts in *orange* and residues without detectable shifts in *cyan*. *Bottom panels*: Differential HDX relative fractional uptake, where differential deuterium uptake at 20 s is indicated by the intensity of *blue* coloring. *C*, structure of M-PMV MAPP (*gray*) in complex with CaM (cyan) calculated by HADDOCK with visualized identified cross-links between myrMAPP and CaM. Cross-links meeting the maximal distance of 35 Å (29) are depicted as *green* dashes, cross-links exceeding the maximum distance of 35 Å are depicted as *red* dash. *D*, structure of M-PMV myrMAPP (*gray*) in complex with CaM (*cyan*) calculated by HADDOCK with visualized chemical shifts detected by NMR. Residues with chemical shifts detected in equimolar ratio of proteins in the analyzed myrMAPP sample are colored in *orange*, whereas those detected in the sample with increased concentration of CaM (1:5) are colored in *blue*, which also comprises the *orange* residues. *E*, structure of M-PMV MAPP in complex with CaM calculated by HADDOCK with visualized differential HDX relative fractional uptake. Degree of deuteration is shown as intensity of *blue* and *red* color. *F*, structure of M-PMV MAPP (*gray*) in complex with CaM (*cyan*) calculated by HADDOCK with visualized individual functional regions of MA. Residues in MA involved in MA oligomerization are colored in yellow (43, 44), residues interacting with PI(4,5)P_2_ are shown in *magenta* (39), residues interacting with the M-PMV cytotail are colored in *green* (58). Additionally, residue that interacts with both the M-PMV cytotail and PI(4,5)P_2_ is colored *blue* and residue that interacts with the cytotail and also involved in oligomerization is colored in *red*.

To interpret the features observed on M-PMV myrMAPP and CaM structures using XL-MS, HDX, and NMR, we employed HADDOCK to model the myrMAPP–CaM complex. Although HADDOCK makes only minor alterations to the conformation of the input molecules, it remains one of the most powerful tools for modeling complexes based on ambiguous experimental data such as NMR chemical shifts and XL-MS restraints. Therefore, we used NMR chemical shifts and restraints provided by the XL-MS analysis, together with the structures of M-PMV myrMAPP and CaM, the latter modelled for its complex with truncated HIV-1 MA (Fig. 8*A*) (26) used as input data. As a result of the modeling, six structural clusters were generated, one of which exhibited a markedly lower HADDOCK score (−108.9 ± 11.6) compared to the others. From this cluster, we selected a representative structure of the myrMAPP–CaM complex, onto which we mapped all our experimental data (Fig. 8, *C*–*F*). In this model the second helix of myrMAPP is in close proximity to N-terminal CaM lobe similarly to HIV-1 MA(8–43) (26). Therefore, all restraints given by XL-MS except two (MA41-CaM1 and MA54-CaM17) were satisfied (Fig. 8*C*). Most NMR contacts of myrMAPP with CaM are located in helix III, with additional contacts also in helix IV and in the loop connecting them (Figs. 7*C* and 8*D*). In the model, the loop and the N-terminal region of helix IV are in close proximity to the N-terminal lobe of CaM. At higher excess of calmodulin relative to MA, we observed NMR signal intensity changes in the loop between helices I and II and around the MA-PP cleavage site (Figs. 7*C* and 8*D*). In our model, this loop also interacts with the N-terminal lobe of CaM. The segment surrounding the MA-PP cleavage site is not in direct contact with CaM; therefore, the observed intensity changes are probably due to allosteric conformational changes in this region. This is consistent with observed faster cleavage of myrMAPP to myrMA and PP upon the interaction with CaM, which corresponds with changes in local dynamics in this region. The HDX data are consistent with the predicted secondary structure and surface accessibility of the myrMAPP–CaM HADDOCK model (Fig. 8*E*). The central helix of CaM likely stabilizes the more flexible helix I of myrMAPP, which is positioned at the solvent-exposed surface of the complex. Reduced deuterium uptake in the N-terminal globule of CaM suggests either a conformational closure or stabilization of the loop connecting helices I and II of myrMAPP, both situated near the N-terminal globule and the central helix of CaM in the model. The myrMAPP region 51 to 56 located on the beginning of helix III, which is prominently exposed in the model, exhibits the strongest increase in deuterium uptake upon myrMAPP binding in the myrMAPP-CaM complex.

## Discussion

The role of the N-terminal domain of the M-PMV Gag polyprotein, the MA protein, is to mediate Gag transport to the site of immature particle assembly and to facilitate interaction with the host cell membrane, enabling virus budding. In most retroviruses including M-PMV, this interaction is mediated by a bipartite motif of MA consisting of the basic patch that interacts with the membrane phospholipids and the N-terminal myristoyl that anchors MA in the membrane *via* myristoyl switch mechanism. Additionally, during or immediately after the virus budding, MA is cleaved from Gag by specific viral protease. We previously demonstrated that the myristoyl switch of the M-PMV MA plays a role in viral maturation by inducing conformational changes that expose a protease cleavage site at the C-terminus of MA, thus enabling its separation from the downstream region of Gag polyprotein during viral assembly (32). This finding was further supported by demonstrating that the I51 A mutation in MA, which enhances myristoyl exposure, promotes both membrane interaction and MA proteolytic separation from Gag.

Although M-PMV and HIV-1 share several features in how Gag interacts with the plasma membrane, such as similar secondary structure and N-terminal myristoylation of the MA domain of Gag, there are still substantial differences in the nature of this interaction between the two viruses. The reason is related to the difference in morphogenesis of these two viruses. Unlike HIV, in which individual molecules of Gag interact with the plasma membrane to trimerize, trigger the myristoyl switch and initiate assembly, M-PMV assembles immature particles in the cytoplasm prior to membrane association. These particles then interact primarily with the intracellular membrane vesicles containing membrane-bound Env glycoprotein. The vesicles subsequently travel to the plasma membrane (37). It remains a question what prevents the myristoyl switch from occurring at the intracellular vesicles and induces it only after their fusion with the plasma membrane. A related questions arise: why does M-PMV Gag avoid budding into internal membranes, and what mechanisms prevent activation of the M-PMV protease and subsequent Gag processing within the condensed interior of the preassembled particle, and which conditions may favor protease dimerization and activation in already preassembled intracytoplasmic particle? In HIV-1, it is believed that phosphatidylinositol 4,5-bisphosphate (PI(4,5)P_2_) is the key membrane component that is required for specific interaction of MA domain of Gag with the plasma membrane and its trimerization (38). However, we found that in contrast to HIV, enhanced concentration of PI(4,5)P_2_ in liposomes does not facilitate the interaction of M-PMV MA, which displays the same affinity to membranes of various compositions (39). Together with the fact that M-PMV Gag first interacts with internal membranes, an additional factor is likely required to initially prevent and later facilitate the myristoyl switch, ensuring that subsequent steps, such as budding and proteolytic processing, take place specifically at the plasma membrane.

CaM has been identified as a molecular trigger of the myristoyl switch in HIV-1 MA, binding in a calcium-dependent manner to induce conformational changes of HIV-1 MA and promote myristoyl exposure (24, 25). We found it intriguing to investigate whether M-PMV MA also interacts with CaM, and whether this interaction facilitates the myristoyl switch to induce conformational changes in MA occurring specifically at the plasma membrane.

Our initial MST experiments confirmed a calcium-dependent interaction between M-PMV myrMAPP and CaM, with a dissociation constant of 4.2 μM. Notably, the observed affinity is comparable to that reported for the interaction between HIV-1 MA and CaM (K_D_ ≈ 2 μM) (25) suggesting that the ability of both MA proteins to bind CaM may be a conserved feature across morphogenetically divergent retroviruses. Subsequently, protein cross-linking followed by SDS-PAGE electrophoresis with immunoblot detection and native MS confirmed the formation of the M-PMV myrMAPP-CaM heterodimer. Interestingly, in the reaction mixture containing both CaM and M-PMV myrMAPP, a signal corresponding to the myrMAPP homodimers, which were stronger than in the mixture without CaM, was detected in SDS-PAGE gels. These findings indicate that the CaM–MA interaction facilitates MA oligomerization.

Published *in vitro* data indicate that in HIV-1, exposure of the myristoyl group in myrMA initiates its trimerization. *In vitro*, HIV-1 myrMA exists in a monomer–trimer equilibrium, where the myristoyl group is buried in the monomer and exposed in the trimer (40). HIV-1 MA trimerization is also regulated by factors like Gag polyprotein concentration, pH, and PI(4,5)P_2_ binding (41, 42). Unlike HIV-1 myrMA, M-PMV myrMA does not oligomerize *in vitro* (35) and only non-myristoylated MA occurs in a monomer–dimer–trimer equilibrium (43, 44).

To further confirm that the interaction between M-PMV MA and human CaM occurs also in the M-PMV producing cells, we performed a pull-down assay with the lysate of the HEK293 T cells transfected with vectors encoding both HA-tagged calmodulin and whole M-PMV (Fig. 4). In line with the *in vitro* data, we were able to confirm that the Gag-CaM interaction occurs in the cell lysate and is dependent on the presence of Ca^2+^. Interaction of CaM with the MA domain of the full-length HIV-1 Gag polyprotein was demonstrated by Radding *et al*. (22) using colocalization studies. In the same study, they did not observe any interaction between the HIV-1 capsid (CA) domain and CaM in an *in vitro* overlay assay with 1 mM calcium (22). However, Tzou *et al*. (23) later demonstrated a direct interaction between CaM and the CA domain using isothermal titration calorimetry and NMR spectroscopy.

To verify whether the interaction with CaM induces changes in M-PMV MA that facilitate the myristoyl switch, we used the approach described earlier in Častorálová *et al*. (32), where the myristoyl switch causes a conformational change in the C-terminal part of MA and downstream sequence of Gag, which then undergoes cleavage by viral protease. We confirmed that CaM accelerates cleavage of M-PMV myrMAPP fusion protein by the viral protease, supporting the involvement of the MA–CaM interaction in facilitating the myristoyl switch. To further investigate this, we performed the same cleavage on two mutant variants of myrMAPP that either facilitate or hinder the myristoyl switch. Notably, CaM-induced acceleration of cleavage was observed even in the presence of the amino acid substitution in MA known to impair the myristoyl switch, reinforcing the notion that CaM can actively promote this process. The earlier appearance of CaM-induced cleavage products compared to the membrane-induced myristoyl switch observed in Častorálová et al. (32), may suggest that interaction with CaM enhances myrMAPP cleavage by viral protease even more effectively than interaction with liposomes. However, a direct comparison is not possible because the molar concentration of liposomes and the proportion of myrMAPP bound to them cannot be accurately determined. Using liposome-binding assay, we showed that CaM promotes the association of myrMAPP with liposomes (Fig. 6), likely by triggering the myristoyl switch, thereby facilitating viral protein binding to the plasma membrane. It has been shown that, at elevated intracellular calcium concentrations, CaM localizes near its binding partners, which include a variety of receptors and signaling proteins situated close to the cytoplasmic membrane (45). Additionally, it was proposed that the intracellular Ca^2+^ gradient plays a role in the trafficking of HIV-1 Gag polyprotein (46). The reported enrichment of calmodulin and calcium ions at the cytoplasmic membrane suggests a possible mechanism by which these factors might support the myristoyl switch and membrane binding observed in our system. It is also important to note that HIV-1 is a complex retrovirus which, in addition to structural polyproteins and enzymatic components, encodes several accessory and regulatory proteins, including Tat, Nef, Rev, Vif, Vpr, Vpu, and the antisense protein (ASP). Notably, the Tat protein has been shown to induce an increase in intracellular calcium levels (47). In addition to HIV-1 MA, also Nef and signal peptide fragment of gp160 were shown to interact with calmodulin; however, functional consequences of these interactions remain to be elucidated (21, 48). In contrast, M-PMV does not encode such accessory proteins, and there is no report on calmodulin interaction with M-PMV Env.

Binding of myrMAPP to the membrane likely induces the release of CaM from MA. This is supported by MST measurements, where CaM binds with lower affinity to the non-myristoylated MA, which mimics the MA with exposed myristoyl (Fig. 1). Moreover, CaM was not detected among 38 host cell proteins stably packed into mature M-PMV viral particles (49) further indicating its role as a temporary mediator rather than a permanent component of the complex. Such a CaM-dependent transient mechanism is reminiscent of other well-characterized systems. For instance, the N-terminaly myristoylated oncogenic protein v-Akt (50) uses this modification to enhance its stable membrane association specifically within cholesterol-rich microdomains, which in turn enhances the tumorigenicity of v-Akt–expressing cells (51). CaM serves here as a calcium sensor that shuttle v-Akt to the plasma membrane where its binding to phosphatidylinositol 3,4,5-trisphosphate (PI(3,4,5)P_3_) displaces CaM (52). This displacement allows Akt to anchor firmly to the membrane and undergo activation *via* phosphorylation. Similarly, other proteins such as MARCKS and KRAS4b utilize CaM for reversible membrane targeting, illustrating a widespread strategy wherein calcium-sensing CaM transiently interacts with proteins to regulate their membrane association (53). CaM was also shown to direct protein complexes made of striatin, zinedin, and caveolin to different sub-membrane micro-domains in response to changes in calcium levels (52). Supported by these findings from other proteins systems, we are convinced that M-PMV could also use CaM as a transient regulator of the plasma membrane association of its immature particles.

Structural changes induced by myrMAPP–CaM interaction were further analyzed using three complementary structural proteomics techniques, providing insights into the interaction interface and associated conformational dynamics. XL-MS data showed that the N-terminal parts of both proteins are in close proximity (Fig. 7, *A* and *B*). The higher flexibility of C-terminal part of myrMAPP was also observed by HDX-MS (K93-Q105 region; Fig. 7*E*) and by NMR the line broadening of residues near C-terminus (E98-V103) was observed when the greater excess of CaM was applied. Interestingly, this increased flexibility concerns the region where the myrMA-PP cleavage site is located, which may explain the accelerated cleavage of myrMAPP by viral protease in the presence of CaM.

NMR spectra of M-PMV myrMAPP in the presence of CaM at a 1:1 ratio indicate a fast exchange regime, suggesting a dynamic interaction, but no significant conformational changes of myrMAPP (Fig. S7*A*). Even when CaM was provided in greater excess (1:5), no significant changes were found in myrMAPP spectra. This differs from the interaction of HIV-1 MA with CaM, as reported by Taylor *et al*. (28) where large conformation changes leading to significant extension of MA were observed. The residues of myrMAPP affected by interaction with CaM are primarily clustered in helix III. However, residues located in the first half of helix III are also known to be part of the oligomerization interface of MA. Since both interactions—binding to CaM and MA oligomerization—produce similar effects in this type of NMR spectrum, we cannot distinguish their individual contributions, especially since our data obtained by protein cross-linking with SDS-PAGE visualization indicated that CaM binding enhances MA oligomerization (Fig. 2).

The 1:4 excess of CaM was applied during HDX-MS experiments, where the increased HDX levels in MA peptides within the myrMAPP-CaM complex (Fig. 7*E*) suggest destabilization of alpha-helical secondary structure regions, potentially leading to their unfolding or reorganization. Similarly to NMR performed in the 1:5 CaM excess, the higher deuteration rate was observed in the regions of Q3-R10 and Y11-K27. The higher hydrogen exchange rate and especially EX1 deuteration kinetics of these regions in the myrMAPP-CaM complex, compared to when it is present alone, may indicate a disruption of the hydrogen bond network and cooperative unfolding events. This could lead to destabilization of the hydrophobic pocket in M-PMV MA, which is stabilized by hydrophobic residues such as Y11, I51 and I86, ultimately triggering myristyol switch. It should be also noted that MA residues K20-Y28 constitute the core of the basic patch responsible for the interaction with negatively charged membrane phospholipids (54). Therefore, higher flexibility and/or accessibility of this region may also contribute to better binding of M-PMV MA to liposomes. Taken together, these results support the notion that CaM is pivotal in facilitating the myristoyl switch. Interestingly, in our previous study, where HDX-MS was applied to investigate the structure of both, myr OUT and myr-IN, myrMAPP mutants (32), the K39-I53 and K93–Q105 regions of the myrMAPP myr OUT mutant displayed also increased deuteration compared to myrMAPP WT, reflecting greater structural accessibility at the cleavage site. In contrast, helix I of myr OUT mutant exhibited reduced deuteration relative to the myrMAPP WT, and peptides in this region showed exclusively EX2 kinetics (32). This shift suggests that, without proper CaM engagement, structural rearrangements occur in a way that destabilizes the cleavage site while constraining the conformational dynamics of helix I.

CaM NMR spectra changed more upon binding myrMAPP than myrMAPP spectra did upon binding CaM. Even at the equimolar ratio of CaM to myrMAPP, we observed line-broadening and chemical shift changes of several residues (Fig. S7). Some of these residues are located in methionine rich crevices, which serve as binding domains for various proteins (55). We also found changes in some loops adjacent to globular domains of the protein, indicating that CaM wraps around MA domain, forming an interaction interface in a similar manner as with other CaM-binding proteins (56). These changes are similar to those observed by (27) in spectra upon its interaction with HIV-1 MA, suggesting the CaM adopts a similar conformation when interacting with M-PMV MA. Complementary to NMR findings, the reduced deuteration level of CaM in myrMAPP-CaM complex compared to CaM alone was also detected in HDX experiments, suggesting a conformational closure of CaM. Interestingly, the regions with the most reduced deuteration level overlap with those detected as affected by NMR (Fig. 7, *D* and *F*). The structure of a truncated HIV-1 MA comprising residues 8 to 43 in complex with CaM was published by Vlach *et al*. (26), showing that the conformation of CaM differs from its free form. This is consistent with observations from other CaM–protein complexes, where flexibility of the linker region between the two Ca^2+^ binding lobes enables CaM to act as a Ca^2+^-dependent adaptor protein (57). The truncated HIV-1 MA(8–43), which comprises two helices, binds to CaM in an antiparallel orientation with the N-terminal helix (α1) anchored to the C-terminal lobe of CaM, and the C-terminal helix (α2) of MA(8–43) interacts with the N-terminal lobe of CaM (Fig. 8*A*). When we projected our NMR and data on the M-PMV myrMAPP–CaM complex onto the structure of CaM in a complex with HIV-1 MA(8–43), we found that the regions of CaM located in close proximity to HIV-1 MA(8–43) were also affected in our experiments (Fig. 8*B*).

Our HADDOCK model provided an insight into what the MA-CaM complex probably looks like. As shown in results, it corresponds to experimental data very well, although several regions identified as affected by NMR or HDX did not align with the HADDOCK model. One such region is helix III, which contains a substantial number of residues that display chemical shift perturbations in NMR. Notably, many of these residues are also implicated in myrMAPP oligomerization (Fig. 8*F*) (44). Since we observed myrMAPP oligomerization upon interaction with CaM in our study (Fig. 2), confirmed by MA inter-links (Fig. S6), we propose that chemical shifts observed on helix III are more likely associated with mutual interactions among individual myrMAPP molecules rather than direct binding to CaM. Interestingly, the majority of myrMAPP residues previously identified as interacting with Env cytoplasmic tail are also located within helix III (Fig. 8*F*) (58). Given the proposed MA–cytoplasmic tail complex and the stoichiometry of MA to Env molecules in the virus particle, it is unlikely that both, the cytoplasmic tail and CaM, bind to the same MA molecule simultaneously. This suggests that helix III serves as a versatile interaction domain, capable of mediating multiple interactions of M-PMV MA.Additional differences between our results and the HADDOCK model are within myrMAPP helix I and the helixes of the CaM C-terminal lobe. Although the myrMAPP helix I is located in close proximity to residues of the CaM N-terminal lobe in the model, we detected an increased deuteration rate in this CaM region compared to the free protein. Conversely, lower deuteration rate was detected for the first and last helices of CaM C-terminal lobe, which are not in contact with any nearby residues in the model. We speculate that these two differences may be related. Inspired by the position of HIV-1 MA(8–43) helix I in the complex with CaM (26), where helix I is located directly between first and last helix of CaM C-terminal lobe, we hypothesize that this can reflect a myristoyl switch not represented by the HADDOCK model, as myristoylation could not be included in the docking. In fact, the C-terminal lobe of CaM lies directly opposite myrMAPP helix I, implying that the observed HDX changes may be associated with extrusion of the myristoyl group and/or with progressive destabilization and reorganizing of myrMAPP helix I into this region. Further, this reorganization would lead to the better accessibility of MA-PP cleavage site for viral protease, as this site is spatially protected in the myristoyl-sequerested form of M-PMV MA. Additionally, the suggested repositioning of MA helix I would expose the MA residues previously identified to specifically interact with PI(4,5)P_2_ (Fig. 8*F*), thereby enhancing membrane accessibility (54). This is consistent with our finding that CaM facilitates the binding of myrMAPP to liposomes.

In conclusion, we have demonstrated that M-PMV MA interacts with CaM both *in vitro* and in cell lysates and mapped the interaction interfaces. This binding promotes myrMAPP oligomerization, triggers the myristoyl switch, a key step in membrane association that facilitates cleavage of MA-PP junction by viral protease. Notably, CaM likely dissociates from the myrMAPP complex upon membrane interaction, coinciding with the transition of myrMAPP to the myr OUT state. This observation aligns with the higher affinity of CaM for myrMAPP compared to the myr OUT-like MAPP state, as well as the absence of CaM in the mature M-PMV virion proteome. Structural proteomics data from XL-MS, HDX-MS, and NMR techniques consistently identify helices I and II of myrMAPP, along with their connecting loop, as the primary CaM-interacting regions. This interaction increases flexibility in these segments, particularly helix I, potentially enabling the extrusion of the myristoyl group and subsequent membrane binding. In contrast, helix III appears to be more structurally stabilized during the interaction, possibly contributing to enhanced MA oligomerization. Furthermore, binding of CaM induces increased flexibility in the MA C-terminus and downstream Gag regions, which correlates with elevated proteolytic cleavage at this site.

Our findings thus support a model in which CaM may function as a transient molecular trigger that increases the flexibility of MA, thereby inducing conformational reorganization, promoting membrane interaction and MA oligomerization, and regulating proteolytic maturation. This mechanism highlights the broader role of CaM-mediated interactions in providing precise spatial and temporal control over viral protein function.

## Experimental procedures

### Expression vectors

Plasmids used for the expression of myristoylated and non-myristoylated variants of MAPP of Mason-Pfizer monkey virus corresponded to constructs previously described by Prchal *et al*. (59). Expression vectors encoding myrMAPP variants with the I51 A and A79 V amino acid substitutions were described by Častorálová *et al*. (32). Calmodulin (CaM) was expressed from a commercially available plasmid obtained from Addgene (Plasmid #47598). For the production of non-infectious M-PMV viral particles, the pSARM4 RT vector was used. PSARM4 RT was prepared from pSARM vector, encoding M-PMV genome and kindly provided by Eric Hunter (Emory University), by EMILI cloning method (60) using primers MPMV_RT_D188 A_F - CATTACATGGCGGACATCCTAATAGCTGG and MPMV_RT_D188 A_R - GTCCGCCATGTAATGTATAATATACATTTGTTTCCAGG. An expression construct for N-terminally HA-tagged calmodulin was generated by Gibson assembly, inserting the CaM coding sequence from the commercial plasmid into the pCMV-HA backbone (primers CaM_F - GTTCCAGATTACGCTgctgaccagctgactgagg, CaM_R - GGGCCTCCATGGCCATAAGtcactttgcagtcatc, pCMVHA_F – CTTATGGCCATGGAGGCCCGAATTC, pCMVHA_R – AGCGTAATCTGGAACATCGTATGGGTAC).

### Production of recombinant proteins

MAPP and myrMAPP were produced in *Escherichia coli* BL21 (DE3) cultivated in LB medium, in case of myrMAPP co-transformed by plasmid coding yeast N-myristoyl transferase. The proteins were purified using metal affinity chromatography using Ni-NTA agarose according to a previously published protocol (59). Calmodulin was produced in *E*. *coli* DH5α cultivated in LB medium and purified using HIC according to previously published protocol (61). Uniformly isotopically labeled proteins were produced using M9 minimal medium with [U-^15^N]NH_4_Cl (CIL). The 13 kDa form of M-PMV protease was prepared using a previously published protocol (62).

### Co-immunoprecipitation of cytoplasmic complexes of CaM with M-PMV Gag

For co-immunoprecipitation analysis, HEK293 T cells (3 × 10^6^, STR authenticated) were seeded and 24 h later co-transfected with 5 μg of pSARM4 and 5 μg of pCMV-HA-CaM plasmids (or pCMV HA-for the negative control) using polyethyleneimine (PEI 1 mg/ml) at a transfection ratio of 2:1 PEI:DNA. Four hours post-transfection, the cells were washed and supplied with fresh medium. Cell lysis and co-immunoprecipitation was performed according to the supplier's protocol. Briefly: 48 h post transfection cells were lysed with ice cold lysis buffer (150 mM NaCl, 0.5% Triton X-100, 50 mM Tris-Cl 10 mM CaCl_2_, pH 7.4), supplemented with protease inhibitor cocktail (cOmplete, Roche). Lysates were cleared by centrifugation at 17,000 × *g* for 10 min at 4 °C. The supernatants were incubated with HA-Trap magnetic agarose beads (Chromotek) for 1 h at 4 °C under constant agitation. Beads were then washed four times with wash buffer (250 mM NaCl, 50 mM HEPES, 10 mM CaCl_2_, pH 7.4). Bound fraction was eluted by boiling the beads in 2 × SDS sample buffer (80 μl) for 10 min and subsequently analyzed by SDS–PAGE and Western blot analysis (using either antiCaM or antiMA M-PMV antibodies). SuperSignal West Femto substrate (ThermoFisher) was used for blot development, and images were acquired using a Uvitec Cambridge imaging system.

### Microscale thermophoresis

CaM was fluorescently labeled using Protein Labeling Kit Red-NHS second Generation (Nanotemper Technologies). The labeling reaction was performed according to the manufacturer’s instructions and protein was transferred to the buffer containing 50 mM Tris-HCL, 100 mM NaCl, 0.05% Tween 20, pH 7.4. For the MST measurement, the ligand MAPP protein was subjected to a series of 16 consecutive 1:1 dilutions using the same buffer, either alone or supplemented with 20 mM CaCl_2_. Each diluted solution was mixed with the same volume of labeled CaM with resulting concentration of CaM 50 nM and concentrations of (myr)MAPP ranging from 1 mM to 10 nM. The samples were loaded into Monolith NT.115 Capillaries (NanoTemper Technologies). MST was measured using a Monolith NT.115 instrument (NanoTemper Technologies) at a temperature of 25 °C. Instrument parameters were adjusted to 20% LED power and medium MST power. Data of three independently pipetted measurements were analyzed using MO. Affinity Analysis software version 2.3, (NanoTemper Technologies) using the signal from an MST-on time of 1.5 s.

### Cleavage of myrMAPP variants by M-PMV protease

Cleavage reactions of all myrMAPP variants were carried out using in-house prepared recombinant M-PMV protease, following a previously published protocol (32, 62). Cleavage buffer consisted of 50 mM sodium acetate (pH 5.3), 300 mM NaCl, 20 mM CaCl_2_, and 0.05% β-mercaptoethanol. Each reaction was set up in a total volume of 220 μl, containing equimolar concentrations (∼25 μM) of myrMAPP variant and CaM, where indicated. Protease was added at a concentration of 2 units per reaction (1 unit defined as the amount required to cleave 100 μg of non-myristoylated MAPP within 1 h). Samples (20 μl) were collected at defined time points (15 min, 30 min, 1 h, 1.5 h, 2 h, 3 h, 4 h, 6 h, and 24 h), mixed with 2 × reducing protein loading buffer, and subjected to Tris-Tricine SDS–PAGE for analysis of cleavage efficiency.

### Liposome preparation

To prepare liposomes mimicking the PM inner leaflet (33), individual lipids (purchased from Avanti Polar Lipids, Inc.) were dissolved in chloroform or in chloroform/methanol/water (20:9:1) in the case of PI(4,5)P_2_ and thoroughly mixed to a final lipid concentration of 5 mg/ml. To obtain 250 μl of lipid mixture, we mixed 310 μg of cholesterol, 400 μg of phosphatidylethanolamine, 75 μg of phosphatidylcholine, 290 μg of phosphatidylserine, 38 μg of PI(4,5)P_2_ and 140 μg of phosphatidylinositol. Chloroform was evaporated, and the lipid mixture was resuspended in a protease cleavage buffer or in phosphate-buffered saline (PBS). Liposomes were formed using a mini-extruder (Avanti Polar Lipids, Inc.) with a 100-nm polycarbonate filter.

### Liposome binding assay

Liposome binding was assessed by flotation through a sucrose gradient. For each sample, 20 μl of myrMAPP and CaM were mixed in an equimolar ratio (final concentration ∼100 μM each) in reaction buffer containing 50 mM Tris (pH 7.4), 10 mM NaCl, and 70 mM CaCl_2_. The protein mix was incubated either with 20 μl of liposomes prepared according to procedure described above or with the reaction buffer alone, and the mixtures were incubated for 1 h at 4 °C. After incubation, 75 μl of 67% sucrose was added to each sample, and the entire volume was transferred to an ultracentrifugation tube (ref. number). Samples were overlaid with 120 μl of 40% sucrose and 70 μl of 4% sucrose without mixing the layers. The gradients were centrifuged at 500,000 × *g* for 45 min at 4 °C (Beckmann – rotor number TLA 120.1). Following centrifugation, top and bottom fractions were collected using a Hamilton syringe, mixed with SDS sample buffer, and analyzed by SDS–PAGE.

### Cross-linking

The equimolar mixtures of MAPP and CaM were cross-linked by DSBU (C4 urea cross-linker) and DSPU (C3 urea cross-linker) in 20 mM HEPES, 100 mM NaCl_2_, 10 mM CaCl, 0.01% mercaptoethanol, pH 6. Additionally, a single MAPP sample was cross-linked by DSBU. The final concentration of each protein in a single sample as well as in the mixture was 50 mM. The five-fold and ten-fold molar excesses of cross-linkers to protein were applied when the cross-linking products were visualized by SDS-PAGE. The five-fold molar excess of both cross-linkers was applied for the XL-MS analysis. Reaction was quenched by addition of Tris buffer pH 8 to final concentration of 50 mM. Samples containing the mixture of uniformly ^15^N-labeled and unlabeled MA were cross-linked by DSBU in the presence and in the absence of CaM by the same procedure described above. All the analyzed samples were prepared in technical triplicate.

When the cross-linked proteins were visualized by SDS-PAGE, 10 ug of protein samples were mixed with protein loading buffer, analyzed by Tris Tricine SDS-PAGE with 10% separation gels and visualized by Coomassie Brilliant Blue R-250 (Sigma Aldrich) or by immunoblot analysis using anti-M-PMV MA rabbit polyclonal antibody (Moravian Biotechnology, s.r.o.) or HRP conjugated anti-CaM mouse monoclonal antibody (G-3; Santa Cru Biotechnology Inc.). When the cross-linked proteins were analyzed by XL-MS cross-linked samples were reduced by 50 mM DTT, alkylated by 25 mM IAA and digested by Pierce Trypsin protease, MS Grade (Thermo Scientific) overnight at 37 °C. Resulting peptide samples were acidified by the addition of TFA to a final concentration of 0.5%. Peptides were then desalted by Bondesil-C18 phase (Agilent technologies) and dried.

### LC-MS/MS analysis of cross-linked proteins

For LC-MS/MS analysis samples were diluted by 0.1% TFA and 2 ug were loaded to be analyzed by UHPLC Dionex Ultimate 3000 RSLC nano (Dionex) coupled with Orbitrap Fusion Lumos Tribrid (Thermo Scientific). Peptides were initially captured on a μ-precolumn (Acclaim PepMap 100 C18 HPLC column, 300 μm × 5 mm, particle size 5 μm) and then separated on an EASY-Spray PepMap RSLC C18 column (Thermo Fisher Scientific, 150 μm × 15 cm, particle size 2 μm). The mobile phase flow rate was set to 300 nl/min. Peptides were eluted by mobile phase A (0.1% formic acid in water, *v*/v) and mobile phase B (0.1% formic acid in ACN, v/v). The gradient profile was as follows: 0 min – 5% B, 95% A; 22 min – 40% B, 60% A; 23.1 min – 90% B, 10% A; 25 min – 90% B, 10% A; 25.1 min – 10% B, 90% A; 30 min – 10% B, 90% A. The eluted peptides were immediately ionized (1600 V, 275 °C).

For MS/MS analysis of sample data-dependent acquisition mode was used. MS spectra were scanned using the Orbitrap analyser within a range of 350 to 2000 *m/z* at a resolution of 120,000, maximum accumulation time of 50 ms and AGC value of 40,000. For MS/MS measurement, ions were isolated in a 1.6-Th window and fragmented by higher-energy collisional dissociation with a normalized collision energy of 30%. Precursor ions with charge states ranging from +2 to +6 were selected for fragmentation. MS/MS spectra were scanned using the Orbitrap analyser at a resolution of 30,000 and with AGC values set to 50,000 a dynamic mode for injection time. Dynamic exclusion was enabled with an exclusion duration of 10 s and an exclusion window of 10 ppm. All scans were collected in profile mode.

To analyze myrMAPP inter-links,^15^N-labeled myrMAPP was mixed in equimolar ratio with non-labeled one. Cross-linking experiment was performed with DSBU in the same way as in the case of non-labeled proteins. Dried samples were reconstituted with 30 μl of 0.1% FA and 5 μl of resulting solution was injected using the Agilent 1290 series UPLC system onto a desalting precolumn Luna Omega Polar C18 (5 μm, 100 Å, 0.3 × 30 mm) at a flow rate of 20 μl/min. After 5 minutes, the peptides were separated using an analytical column Luna Omega Polar C18 (3 μm, 100 Å, 0.3 × 150 mm) heated to 50 °C at a flow rate of 10 μl/min. The acetonitrile gradient of 5% to 35% over 35 min was used. The chromatographic system was directly connected to an electrospray source of the solariX XR (Bruker Daltonics) mass spectrometer equipped with a 15T superconducting magnet. The sprayed peptides were analyzed in a positive mode with 1M transient data points with the m/z range of 250-2500. For the MS analysis, 0.4 s ion accumulation was used with 4 accumulated scans per spectra.

### XL-MS data analysis

Raw mass spectrometry files were converted to Mascot generic format (MGF) using MSConvert (ver. 3.0.22045). The converted MGF files were then imported into MeroX software (ver. 2.0.1.4) to identify cross-linked peptides (63). The sequence of M-PMV MA C-terminally extended with 16 amino acids of the downstream phosphoprotein (MAPP) domain of M-PMV Gag and the sequence of human calmodulin-1 (UniProt entry P0DP23) without initiator methionine was used. Trypsin was used as a protease with a maximum of three missed cleavage sites. Cysteine carbamidomethylation and methionine oxidation were employed as variable modifications. Peptides with a minimum length of three amino acids were included in the search. The lower mass limit was set to 200 Da and the upper mass limit was set to 8000 Da, with a signal-to-noise ratio of 2.0. Ion types b and y were searched for. The first linkable site was defined as the amino group of lysine and the N-terminal amine group whereas the second linkable site included lysine, serine, threonine and tyrosine (34). The mass tolerance for precursor and fragmented ions was set to 10 ppm and 15 ppm, respectively. The results were filtered at a 5% false discovery rate at the spectrum level. All acquired cross-links were inspected manually and low-quality linkages, homeotypic linkages and N-terminal linkages of MAPP were not considered, as well as the linked consecutive peptides. The data obtained from LC-MS analysis of mixed-isotope labeled samples were deconvoluted using the SNAP 2.0 algorithm in the DataAnalysis 4.4 software. m/z values from MS spectra together with intensity value were exported to.txt file that can serve as input for LinX software (64). Our XL mass spectrometry proteomics data are deposited in the database of the ProteomeXchange Consortium *via* the PRIDE (65) partner repository under dataset identifier PXD067304. All identified linkages are listed in Tables S1 and S2 and fragmentation spectra of cross-links between myrMAPP and CaM are shown in Figure S4.

### Native mass spectrometry

The buffers of myrMAPP and CaM sample were exchanged with 150 mM ammonium acetate by Amicon Ultra–0.5 Centrifugal Filters with 3 kDa MWCO (Millipore) according to the manufacturer’s instructions. Proteins were mixed in equimolar ratio to prepare myrMA-CaM mixture of the 150 μM concentration of both proteins in mixture (300 μM total protein concentration) or with 150 mM ammonium acetate to prepare individual protein samples of 150 μM concentration. All samples were measured in the absence of calcium acetate or in the presence of 0.5 mM calcium acetate. Samples were inserted into pulled glass capillary with a tip I.D. of about 1 μm and mounted on an in-house built nESI source connected to Waters SELECT SERIES Cyclic Ion Mobility instrument. Voltage was applied using a platinum wire, and sample was sprayed directly into the inlet of the mass spectrometer. Data was acquired over an m/z range of 50 to 4000, with nESI voltage between 0.8 and 1 kV, source temperature of 30 °C, Sampling cone setting between 10 V and trap and transfer collision energies of 10. The instrument was further tuned for increased high-mass ion transmission by increasing the quadrupole Pre-filter voltage to 20V, Ion Guide RF to 700V and Transfer RF to 800V. The acquisition time was 3 min.

Raw spectra were inspected manually and deconvoluted in UniDec software version 7.0.2. All native mass spectrometry data as well as the parameters of data processing are deposited in the database of the ProteomeXchange Consortium *via* the PRIDE (65) partner repository under dataset identifier PXD067292.

### HDX-MS

M-PMV myrMAPP was diluted in 10 μl HEPES buffer (25 mM, pH 7.4, 150 mM NaCl, 10 mM Ca2+) to a concentration of 10 μM, mixed with 10 μl of 40 μM human calmodulin in HEPES buffer (25 mM, pH 7.4, 150 mM NaCl, 10 mM Ca^2+^) to get a theoretical population of bound state of myrMAPP to at least 52%. After 10 min of pre-incubation, samples were diluted with 20 μl D_2_O HEPES buffer (HEPES buffer (25 mM, pD 7.4, 150 mM NaCl, 10 mM Ca^2+^) to create a 50% deuterium labeling mixture. Labeling was conducted over multiple timepoints in 4 °C (5 s, 20 s, 2 min, 20 min and 2 h) and 40 μl aliquots were quenched 1:1 (v/v) with 500 mM glycine, 6 M urea buffer (pH 2.3) and then flash frozen in liquid nitrogen. Samples were prepared in triplicates for each timepoint and thawed immediately prior to HX-MS2 analysis. Samples for sequence mapping were processed in the same way, except D_2_O was replaced with H_2_O in the labeling phase. Data acquisition was carried out on SELECT SERIES Cyclic IMS instrument coupled to an M-class nanoACQUITY UPLC system and an HDX manager (Waters Corporation, MA, USA). All samples were prepared manually and injected into the cold compartment of the autosampler set to 4 °C. The injected proteins were on-line digested on a protease column (Nepenthesin-2, 2.1 x 20 mm, AffiPro, temperature of digestion: 25 °C), trapped on Acclaim PepMap 100 C18 HPLC Trap Cartridge (C.N.: 160,434, Thermo Scientific) and separated on Thermo Hypersil GOLD C18 Selectivity HPLC Column 1 mm × 50 mm (C N.: 25,002–051030, Thermo Scientific) using a 22 min 5% to 35% solvent B gradient. Solvent A was 0.4% FA in H_2_O and solvent B was 0.4% FA in ACN. The flowrate was 100 μl/min for loading, digestion and desalting, and 50 μl/min for separation. Data were collected in MSe mode for mapping and MS1 mode for deuterated samples, using a standard ESI source with Source Temperature set to 100 °C, desolvation gas temperature set to 350 °C, desolvation flow to 800 l/h, Cone Voltage 45V, Capillary voltage 3.15 kV and Source Offset 20V. Manual quadrupole profile optimized for peptides was used. The mass range was set to m/z 350 to 1200. Detailed instrument setup parameters have been uploaded alongside raw data to the PRIDE data repository.

### HDX data analysis

Non-deuterated peptides were identified using PLGS, version 3.0.3 (Waters) using replicates of non-deuterated control samples. Processing parameters were as follows: automatic chromatographic peak width, automatic MS TOF resolution, lock mass window 0.25 Da, low energy threshold 150.0 counts and elevated energy threshold 30.0 counts. Workflow parameters were set to automatic peptide tolerance, 3 minimum fragment ion matches per peptide, 7 minimum fragment ion matches per protein and false discovery rate was set to 100 as instructed by the manufacturer. Raw MS data were imported into Mass Spec Studio, version 2.4.0.3643 (Trajan Scientific and Medical) and filtered with the MS Mass Tolerance set to 50 ppm, integration range 0.25 min, RT variance 0.5 min and XIC extraction mass tolerance 10 ppm. Those peptides meeting the filtration criteria were processed automatically by Mass Spec Studio followed by manual inspection of all spectra. All HDX mass spectrometry data as well as the parameters of data processing are deposited in the database of the ProteomeXchange Consortium *via* the PRIDE (65) partner repository under dataset identifier PXD067350.

### NMR spectroscopy

All NMR data were collected on a Bruker AvanceIII 600-MHz NMR spectrometer equipped with a cryoprobe (Bruker BioSpin, GmbH, Germany). Spectra of backbone NH groups were collected using a HN-HSQC experiments. The data were processed with TopSpin (Bruker BioSpin GmbH, version 3.6) and analyzed using Sparky 3 NMR software (T. D. Goddard and D. G. Kneller, SPARKY 3, University of California, San Francisco).

### Calculation of the MA-CaM complex model

The structure of the MA-CaM complex was calculated by the HADDOCK 2.4 webserver (66, 67). As an input for docking, we used the structure of myrMAPP (35) and structure of CaM after interaction with truncated HIV-1 MA (26). The residues determined by NMR spectroscopy were used as active. Linkages identified by XL-MS were used as unambiguous restraints. Passive residues were determined automatically. Docking was performed according to the standard protocol provided by the webserver. The resulting structures were clustered by structural similarity based on root mean square deviation (RSMD), and the cluster with the lowest overall energy was selected. The structure was visualized using PyMOL 3.1.4.1 (Schrödinger, LLC) and UCSF ChimeraX 1.10.1 software (68). Some figures were created in part using Biorender software (https://www.biorender.com/).

## Data availability

Mass spectrometry data are deposited in the database of the ProteomeXchange Consortium *via* the PRIDE partner repository under dataset identifiers PXD067292 (native MS data), PXD067304 (XL-MS data) and PXD067350 (HDX-MS data).

Reviewer access details - Log in to the PRIDE website using the following details.

Project accession: PXD067292 Token: d0vj013C2r9e

Project accession: PXD067304 Token: IqNqpHu7an x 4.

Project accession: PXD067350 Token: mM58Hw2TJfdi.

## Supporting information

This article contains supporting information.

## Conflict of interest

The authors declare that they have no conflicts of interest with the contents of this article.
