# Supplementary material for: Structural and functional insights into calmodulin-mediated lipid binding and proteolytic cleavage of the M-PMV matrix protein
Source: J Biol Chem. 2025 Dec 23;302(2):111102. doi: 10.1016/j.jbc.2025.111102 (PMC12858339; doi:10.1016/j.jbc.2025.111102)
Supplement: Table S2 [file mmc3.docx]

**Suplements:**


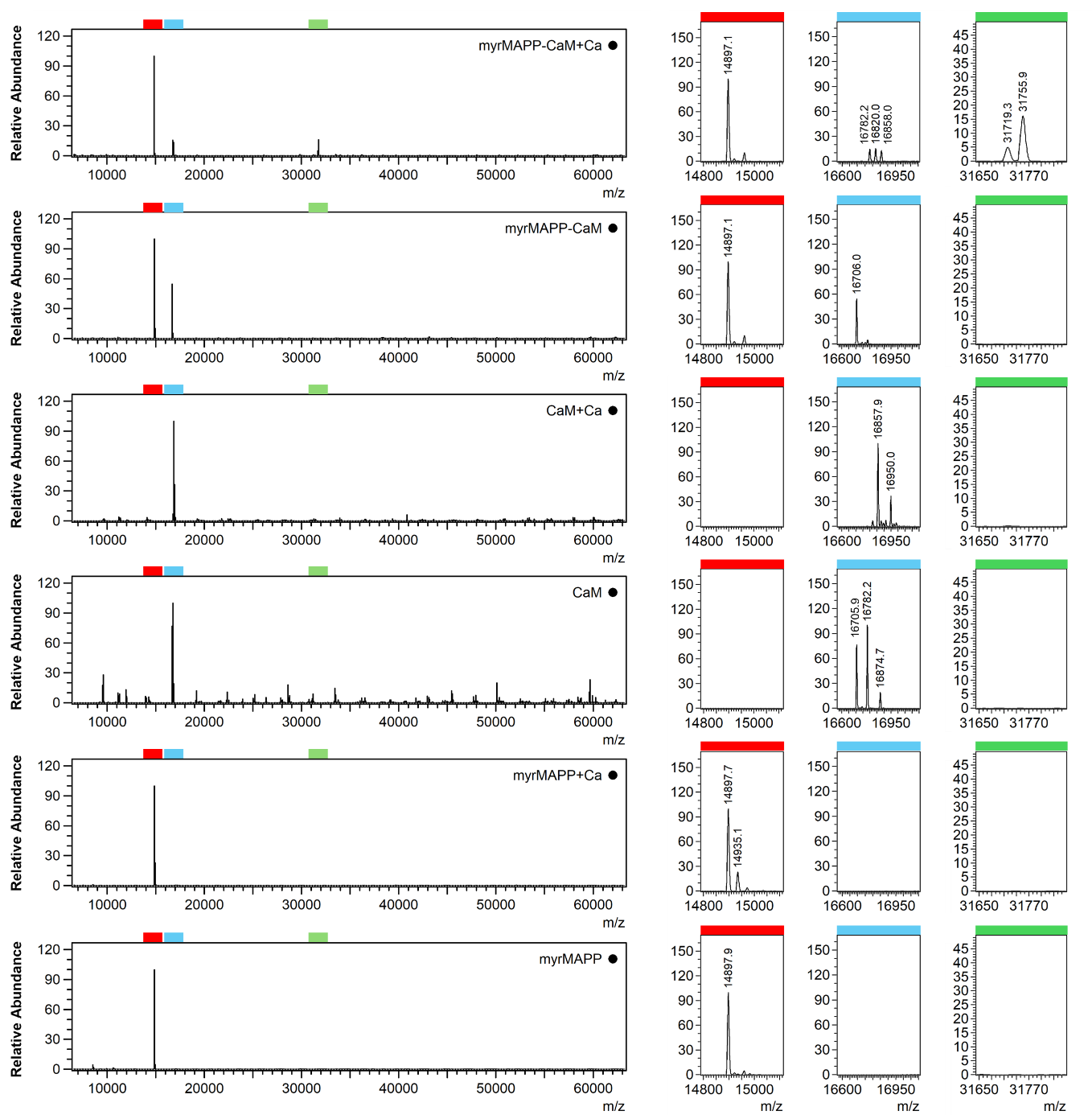


**Figure S1. Deconvoluted native mass spectra of myrMAPP, CaM and their mixture.**

Mixed samples of myrMAPP and CaM, as well as individual protein samples, either with or without 0.5 mM calcium acetate, were measured by native mass spectrometry. Mixed samples without calcium acetate and all samples of individual proteins served as control samples. Signals of the myrMAPP and CaM complex appeared only in the presence of calcium acetate (signal of 31,719 Da represent the signal of myrMAPP-CaM complex with three calcium ions bound to CaM and signal of 31,756 Da represent signal of myrMAPP-CaM complex with four calcium ions bound to CaM). In individual protein samples without calcium acetate, as well as in the mixed samples without calcium acetate, signals of 14,897 Da represent signals of myrMAPP, signals of 16,706 Da represent CaM without calcium ion bound, signals of 16,782 Da represents CaM with two calcium ions bound, signal of 16,820 Da represents CaM with three calcium ions bound and signals of 16,858 Da represents CaM with four calcium ions bound. The color bars above the spectra in the left panel correspond to the enlarged m/z regions in the right panel marked with the same color.


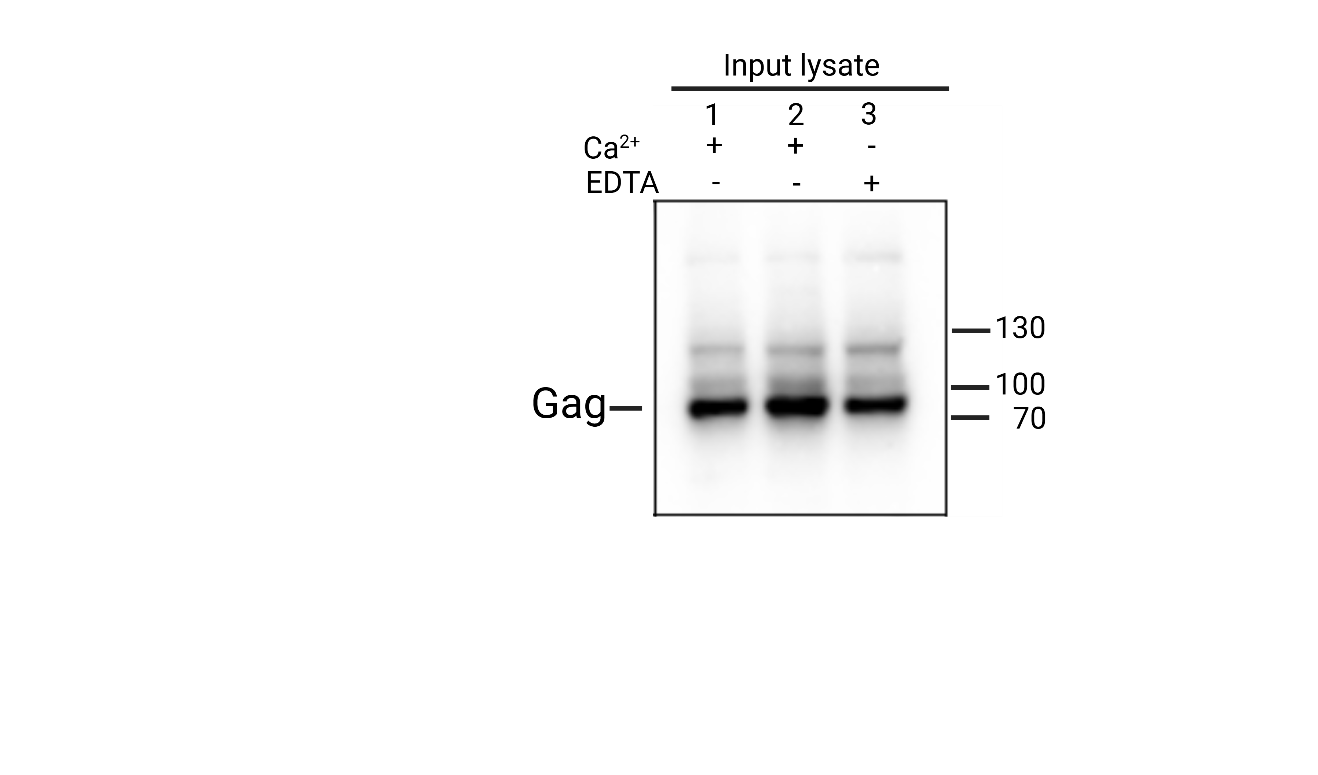

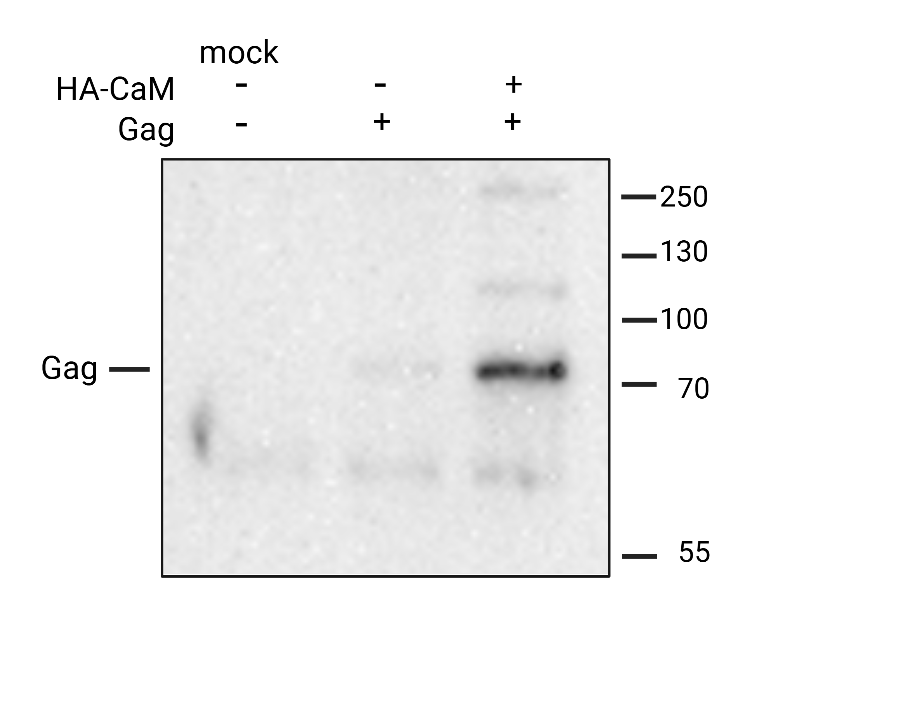


**Figure S2. Control samples for co-immunoprecipitation of M-PMV Gag and HA-tagged CaM**

Left panel: analysis of Gag concentrations in the lysates used for co-immunoprecipitation of M-PMV Gag and HA-tagged CaM in HEK293T cells shown in Figure 4; Right panel: confirmation that the band of lower molecular weight (about 60 kDa) is nonspecific signal present also in the mock sample of non-transfected cells.


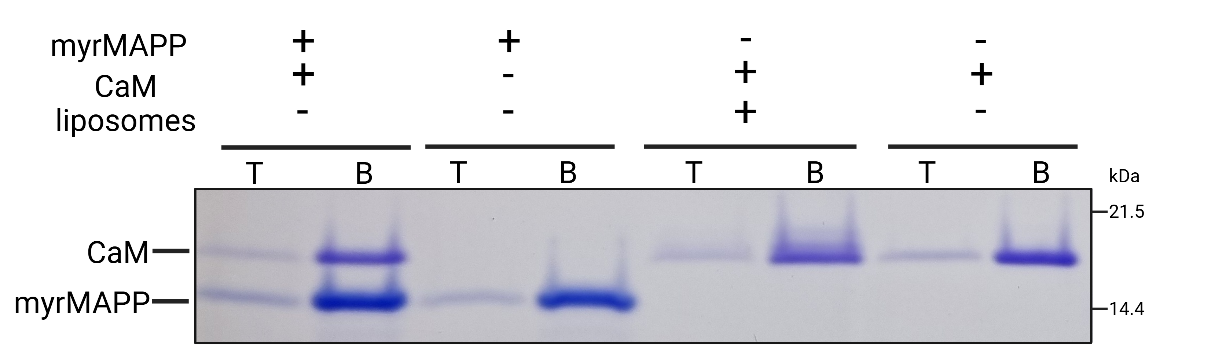


**Figure S3. SDS-PAGE analysis of myrMAPP and CaM distribution in fractions obtained by liposome binding assay.**

Liposome binding assay was performed to assess the distribution of myrMAPP and CaM between top (T) and bottom (B) fractions in the presence or absence of their counterparts or liposomes. The weak signals of both proteins in top fraction (liposome bound fraction) of samples without liposomes are the basal signals of both proteins given by the method. CaM itself does not bind to the liposomes as its signal in the top fraction of the sample containing both CaM and liposomes, does not change compared to signal of CaM in the absence of liposomes.

Figure S4: Fragmentation spectra of identified myrMAPP-CaM cross-links - Separate word file


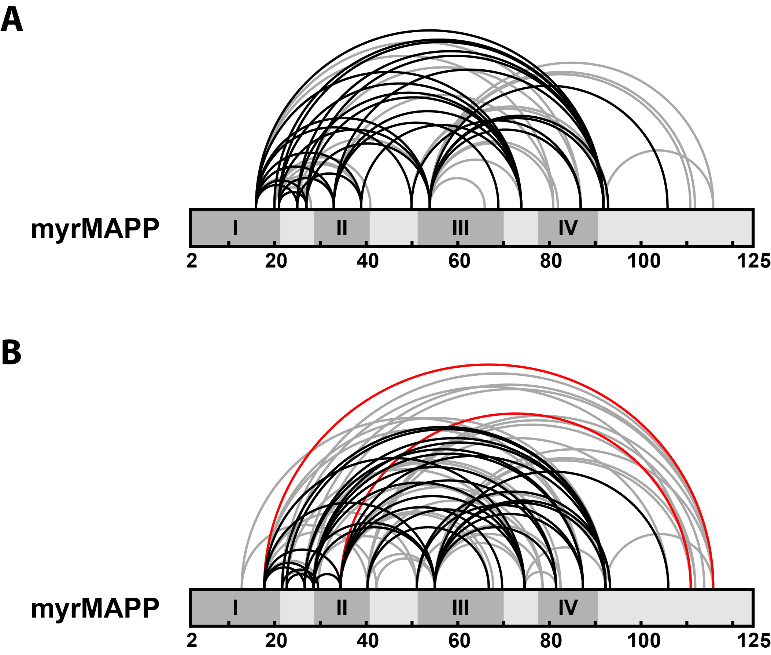


**Figure S5: Linkages between residues of myrMAPP identified by the use of DSBU in the presence of CaM (A) and in the absence of CaM (B).**

Black lines represent linkages identified in all three replicates; gray lines represent linkages identified in one or two replicates; red lines represent linkages identified in all three replicates of individual myrMAPP sample and in none of replicates of mixed myrMA-CAM sample pointing to the possible spatial separation of N- and C-terminal myrMAPP regions in the presence of CaM.

Table S1. myrMAPP and CaM cross-links obtained by the use of DSBU - Separate excel file

Table S2. myrMAPP and CaM cross-links obtained by the use of DSPU - Separate excel file


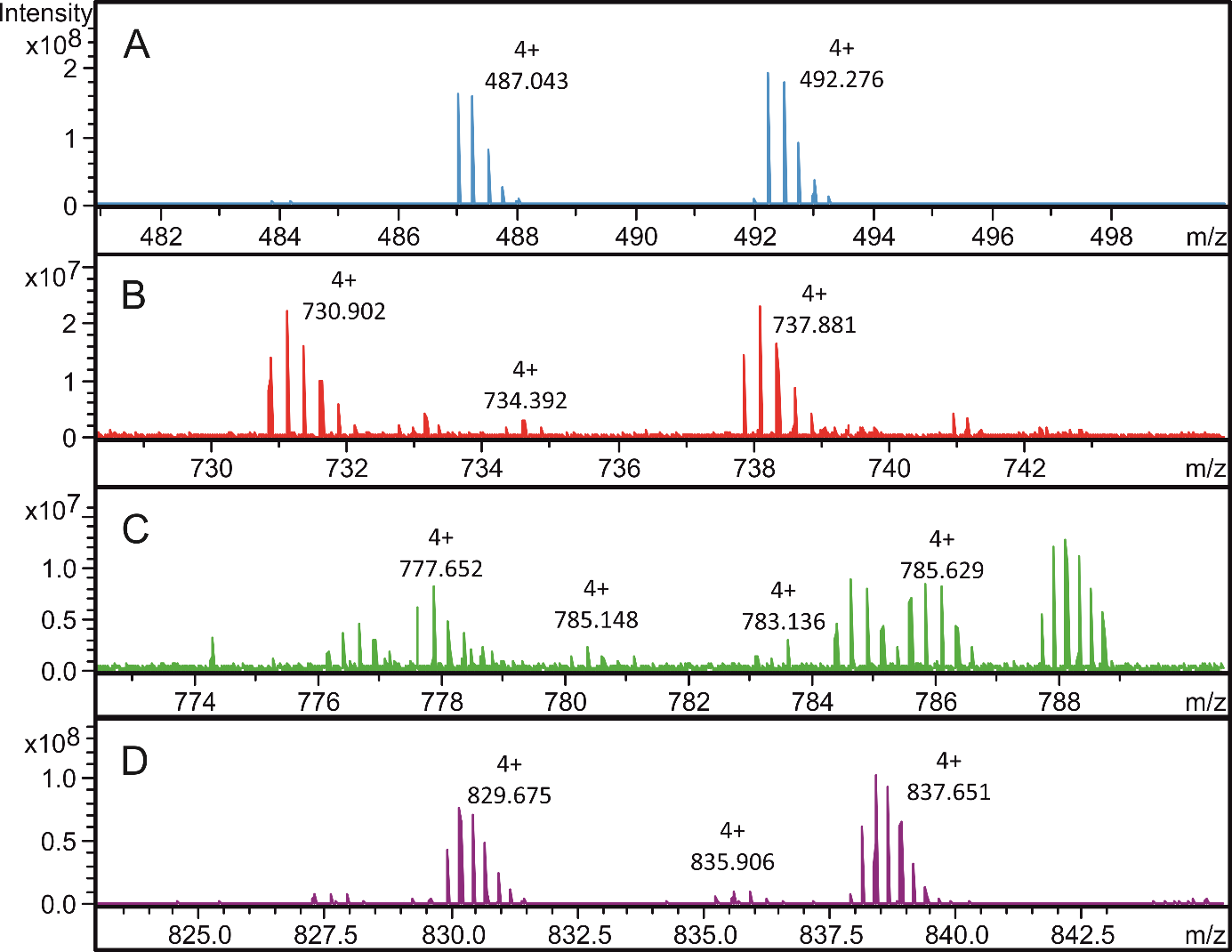


**Figure S6. Mass spectrometric identification of myrMAPP inter-links via mixed-isotope labeling approach coupled with XL-MS.** (A) signals of myrMAPP inter-link K16-K25 (YVEQLKQALK – GVKVK), (B) signals of myrMAPP inter-link K16-K33 (YVEQLKQALK – YADLLKFFDFVK), (C) signals of myrMAPP inter-link K27-K54 (VKYADLLK – DTCPWFPQEGTIDIKR), (D) signals of myrMAPP inter-link K25-K39 (GVKVK – FFDFVKDTCPWFPQEGTIDIK). Complementary ^15^N labeled doublets in the samples of MA-CaM complex modified by DSBU prove the linkage between labeled and non-labeled peptides.


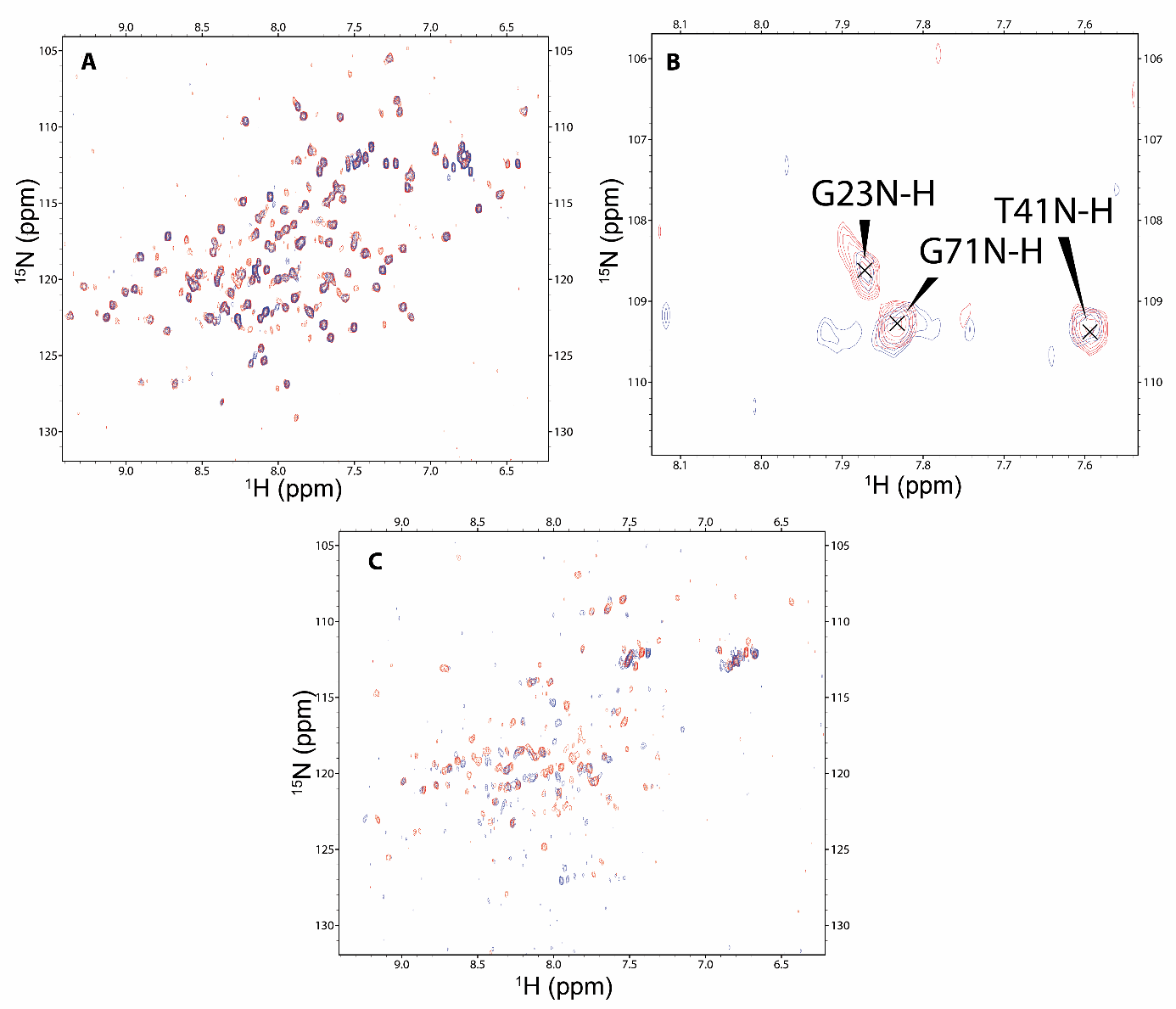


**Figure S7. NMR Spectra of M-PMV myrMAPP andCaM before and after their interaction**

(A) Overlaid HN-HSQC spectra of free ^15^N-labeled myrMAPP (red) and ^15^N-labeled myrMAPP with equimolar addition of unlabeled CaM (blue). (B) Detail of overlaid HN-HSQC spectra of free ^15^N-labeled myrMAPP (red) and ^15^N-labeled myrMA with five-fold excess of unlabeled CaM (blue). (C) Overlaid HN-HSQC spectra of free ^15^N-labeled CaM (red) and ^15^N-labeled CaM with equimolar addition of unlabeled myrMAPP (blue).


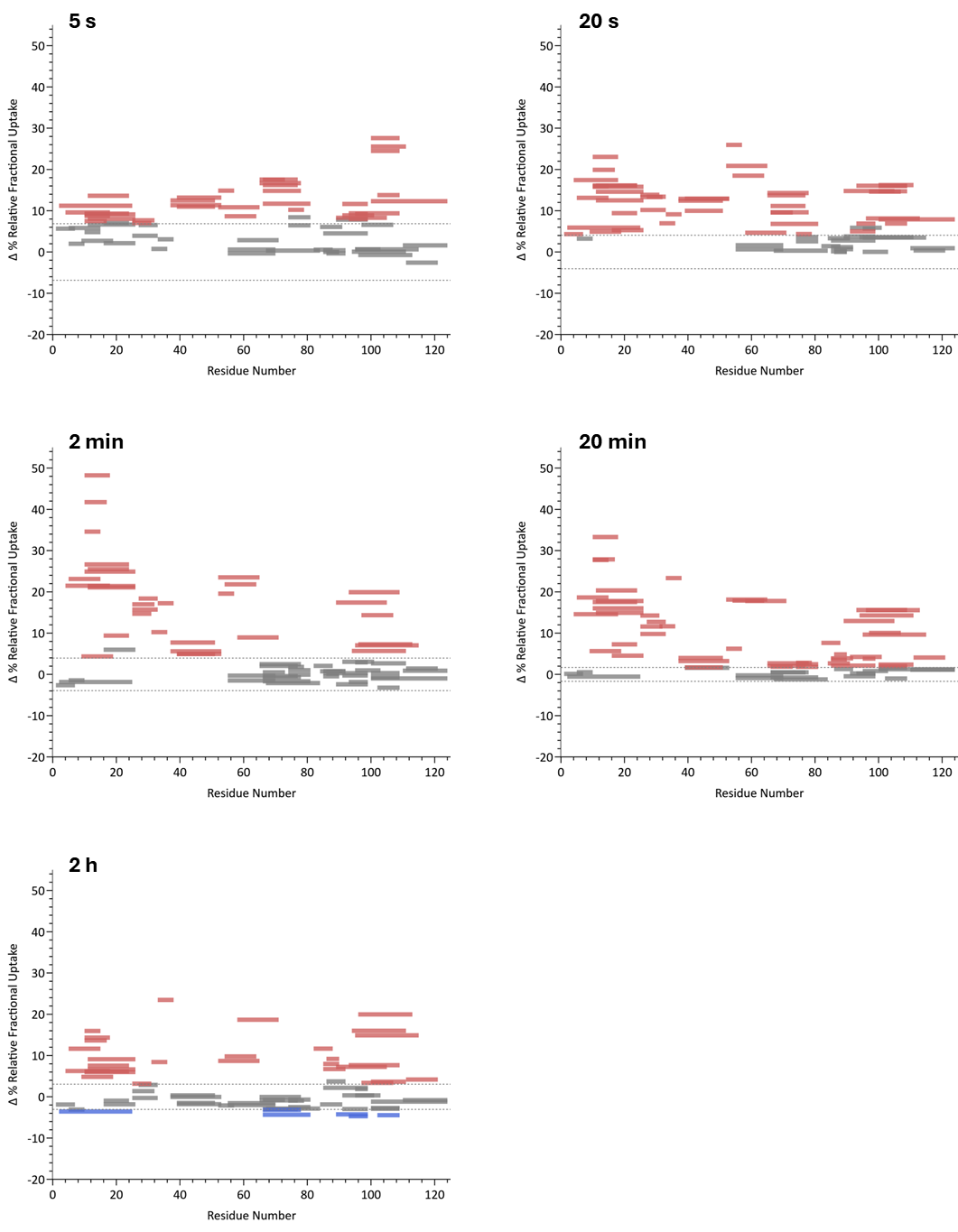


**Figure S8:** Woodsplots of myrMAPP when bound to-CaM versus myrMAPP alone. Red bars show peptides with significant increase of deuteration upon the interaction with CaM, and blue bars show peptides with significantly decreased deuteration upon the interaction. The grey bars show peptides classified as statistically unsignificant.
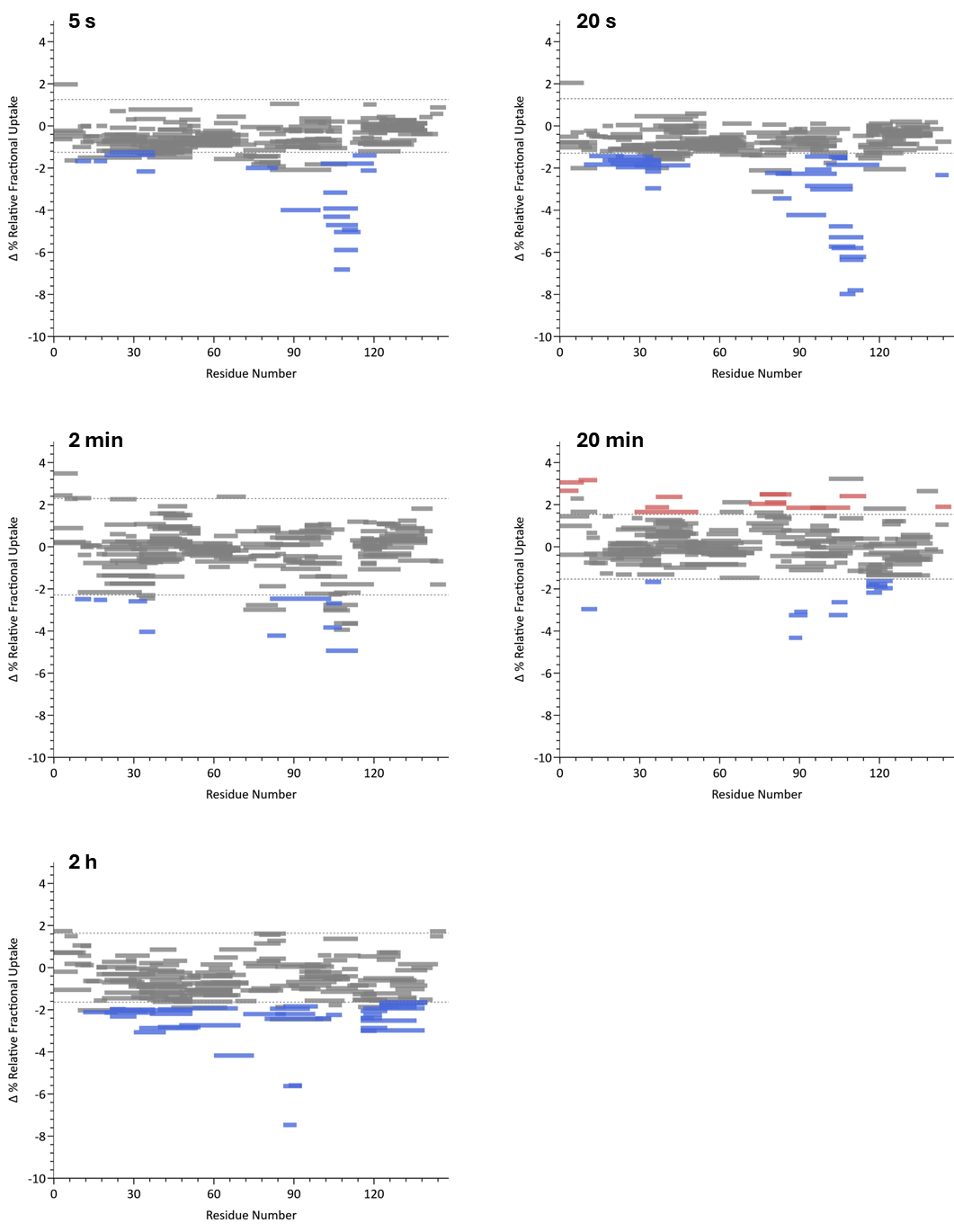


**Figure S9:** Woodsplots of CaM when bound to-CaM versus CaM alone. Red bars show peptides with significant increased deuteration upon the protein interaction, and blue bars show peptides with significant decreased deuteration upon the protein interaction. The grey bars show peptides classified as statistically unsignificant.


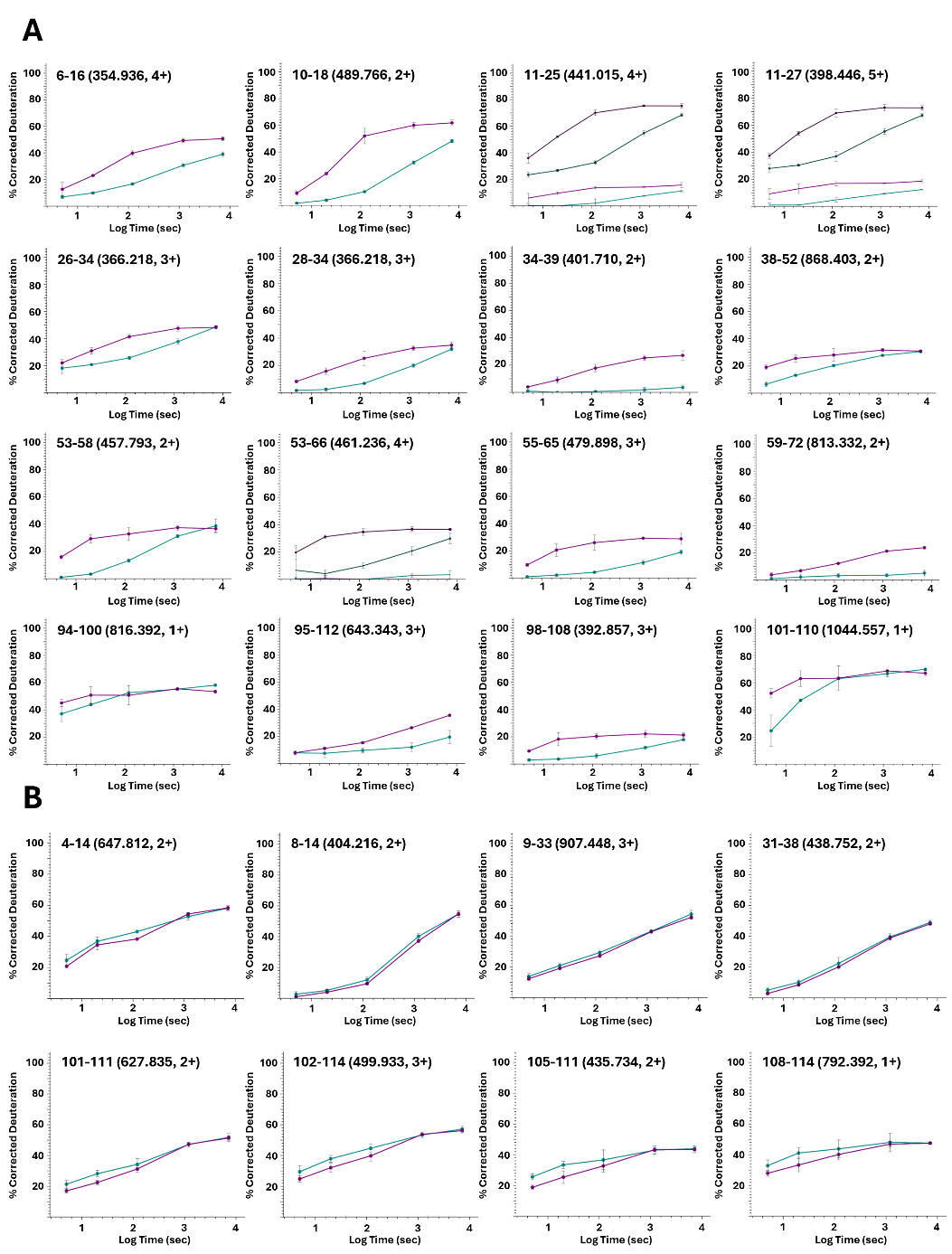


**Figure S10: Representative deuteration kinetics plots of individual peptides.**

(A) myrMAPP in myrMAPP-CaM complex (purple lines) vs. myrMAPP (green lines). (B) calmodulin in myrMAPP-CaM complex (purple lines) vs. calmodulin (green lines)
