# Supplementary material for: Structural and functional insights into calmodulin-mediated lipid binding and proteolytic cleavage of the M-PMV matrix protein
Source: J Biol Chem. 2025 Dec 23;302(2):111102. doi: 10.1016/j.jbc.2025.111102 (PMC12858339; doi:10.1016/j.jbc.2025.111102)
Supplement: Supplement [file mmc4.docx]

**Fig S4: MeroX identification of myrMAPP-CaM cross-links**


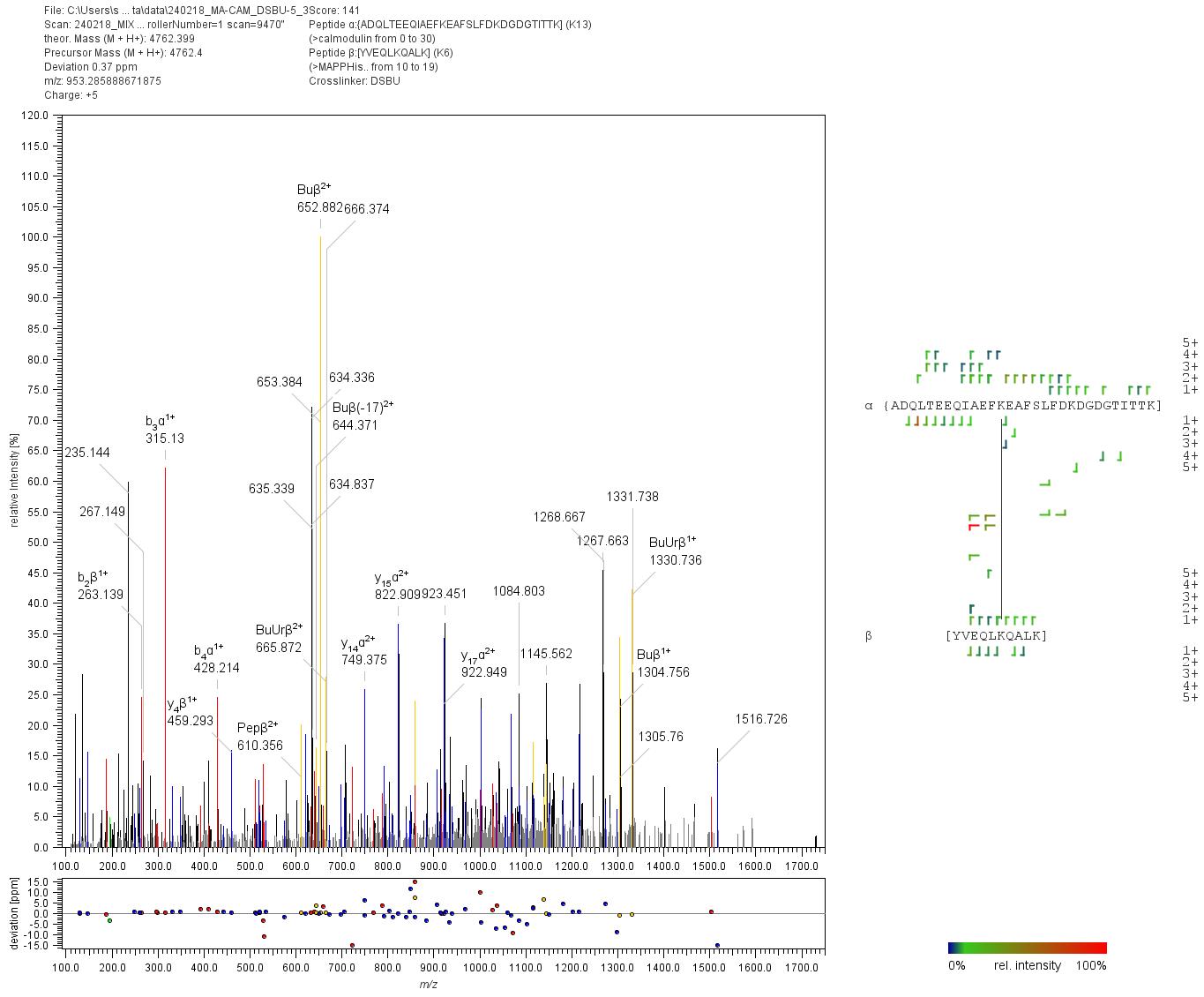


**DSBU: myrMAPP_K16-CaM_K13**

Linkage: {ADQLTEEQIAEFKEAFSLFDKDGDGTITTK] - [YVEQLKQALK] (K13-K16)

Peptide α: {ADQLTEEQIAEFKEAFSLFDKDGDGTITTK] (K13); (> CaM from 0 to 30)

Peptide β: [YVEQLKQALK] (K6); (>myrMAPP from 11 to 20)


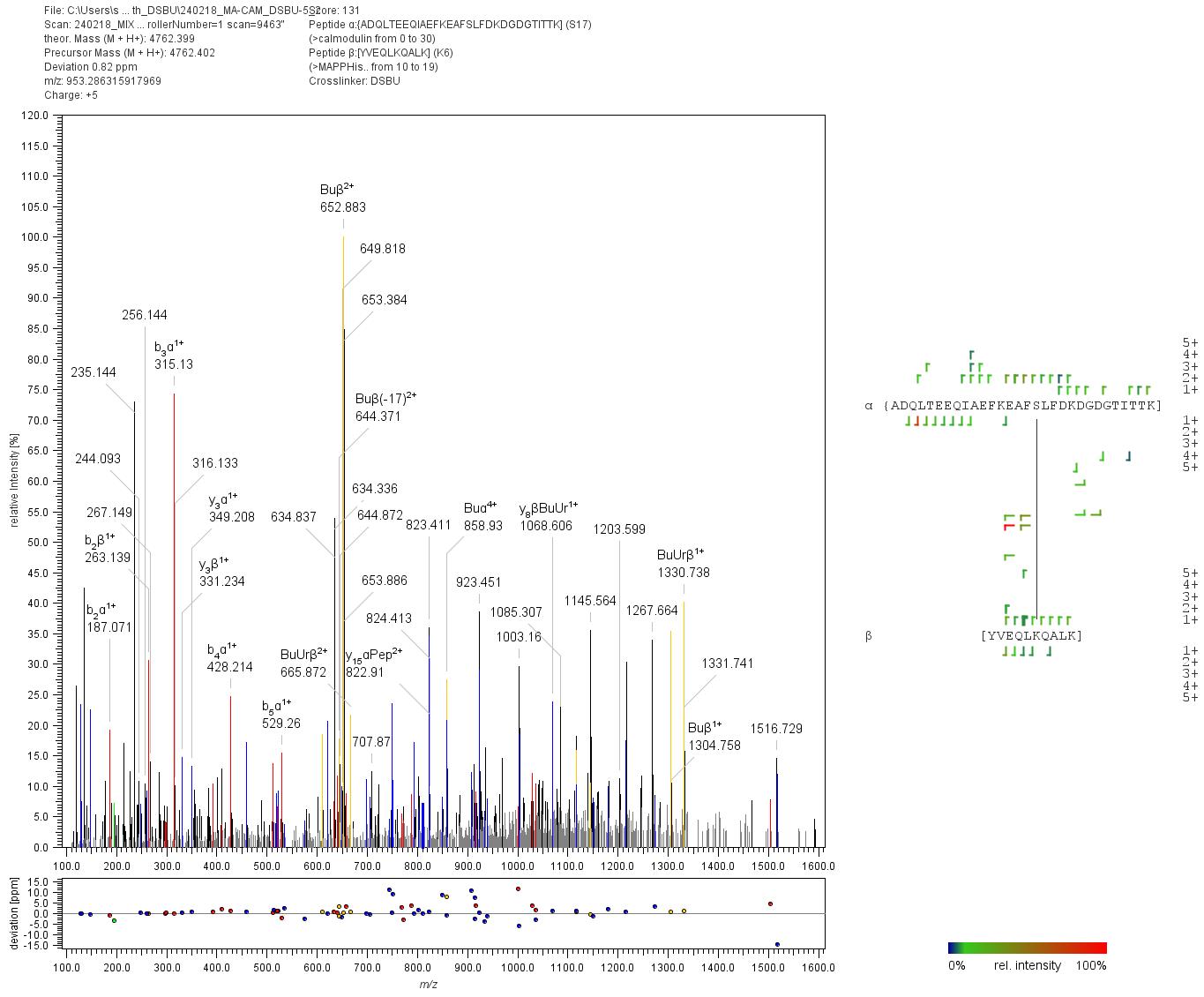


**DSBU: myrMAPP_K16-CaM_S17**

Linkage: {ADQLTEEQIAEFKEAFSLFDKDGDGTITTK] - [YVEQLKQALK] (S17-K16)

Peptide α: {ADQLTEEQIAEFKEAFSLFDKDGDGTITTK] (S17); (> CaM from 0 to 30)

Peptide β: [YVEQLKQALK] (K6); (>myrMAPP from 11 to 20)


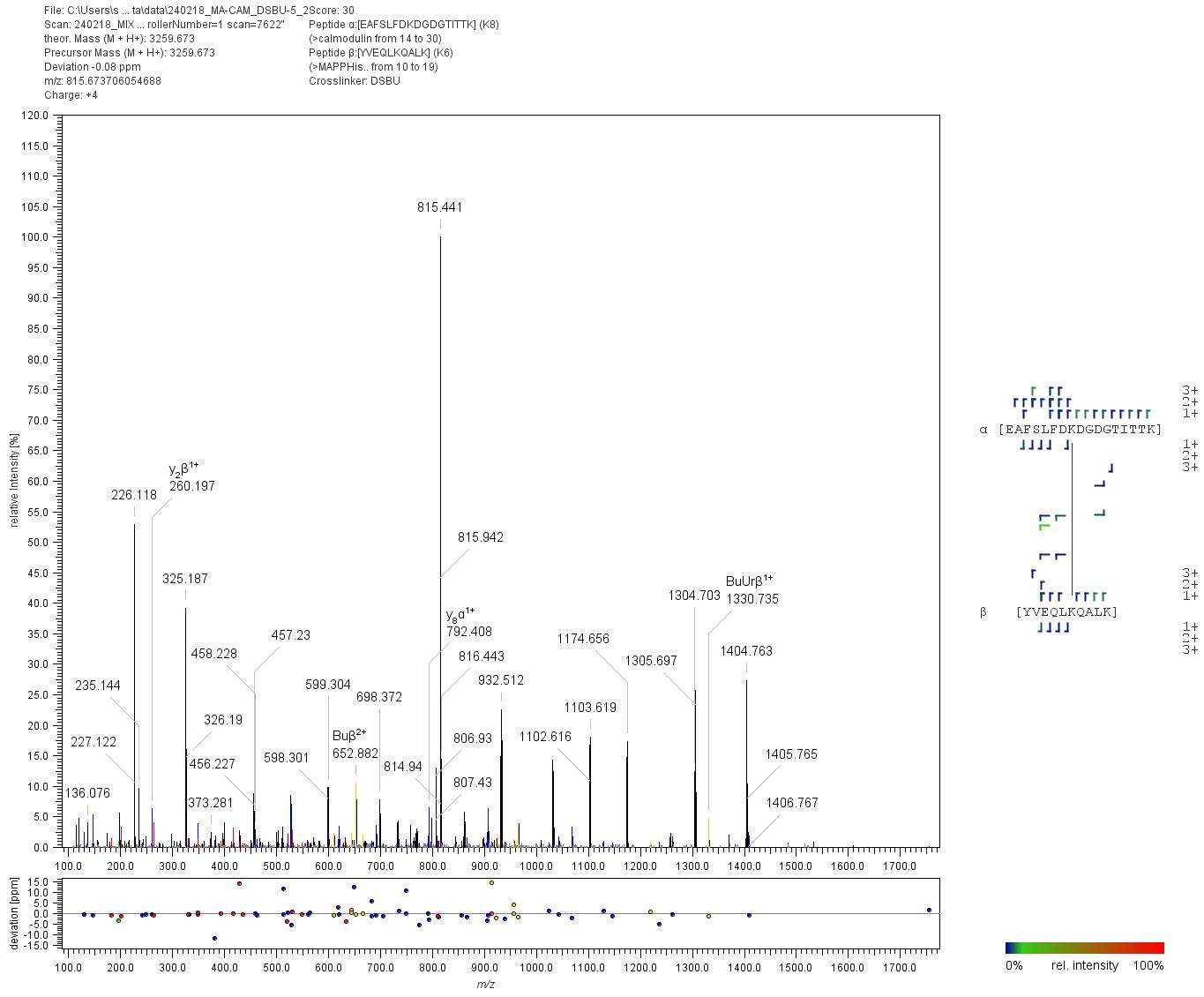


**DSBU: myrMAPP_K16-CaM_K21**

Linkage: [EAFSLFDKDGDGTITTK] - [YVEQLKQALK] (K21-K16)

Peptide α: [EAFSLFDKDGDGTITTK] (K8); (> CaM from 14 to 30)

Peptide β: [YVEQLKQALK] (K6); (>myrMAPP from 11 to 20)


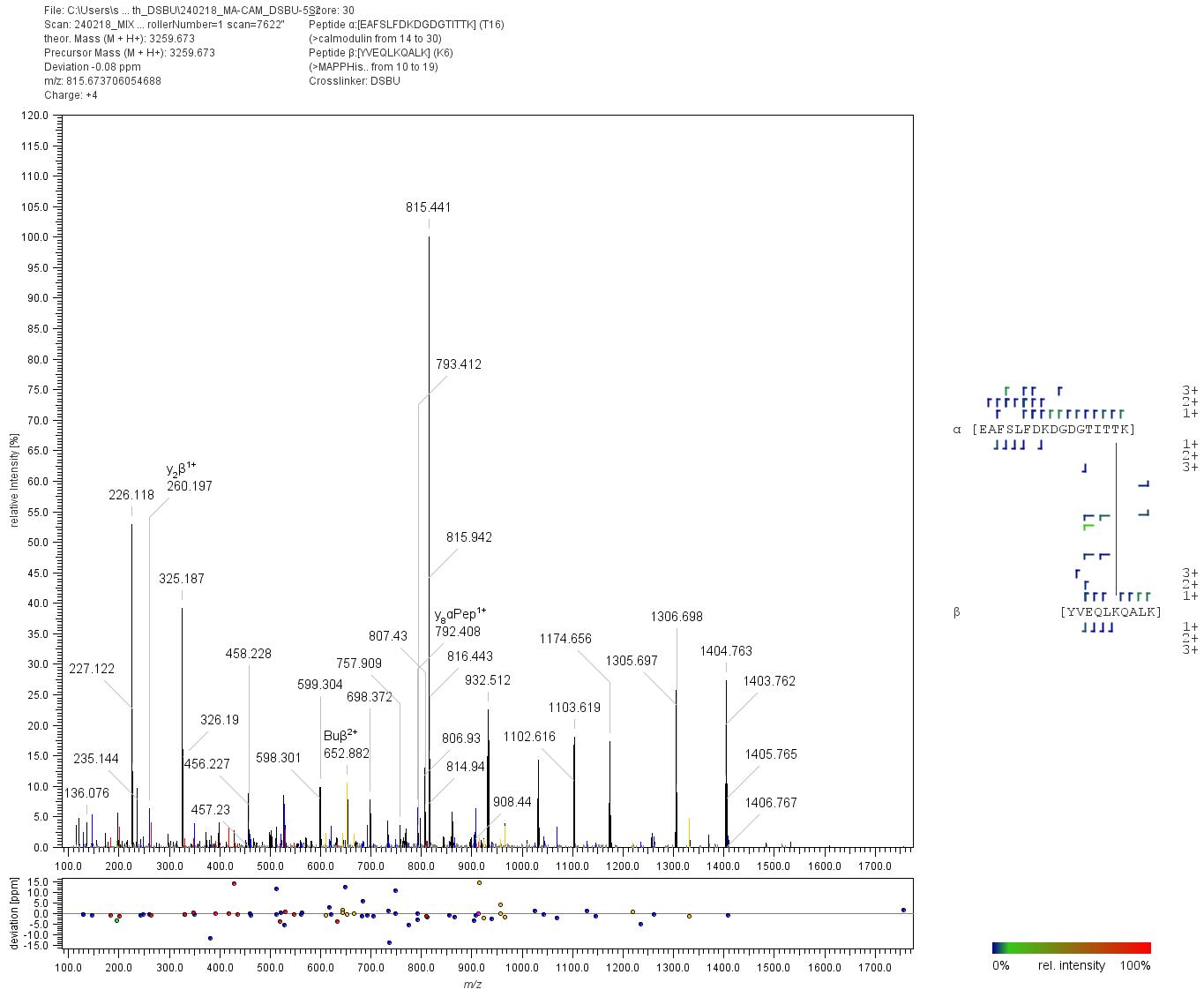


**DSBU: myrMAPP_K16-CaM_T29**

Linkage: [EAFSLFDKDGDGTITTK] - [YVEQLKQALK] (T29-K16)

Peptide α: [EAFSLFDKDGDGTITTK] (T16); (> CaM from 14 to 30)

Peptide β: [YVEQLKQALK] (K6); (>myrMAPP from 11 to 20)


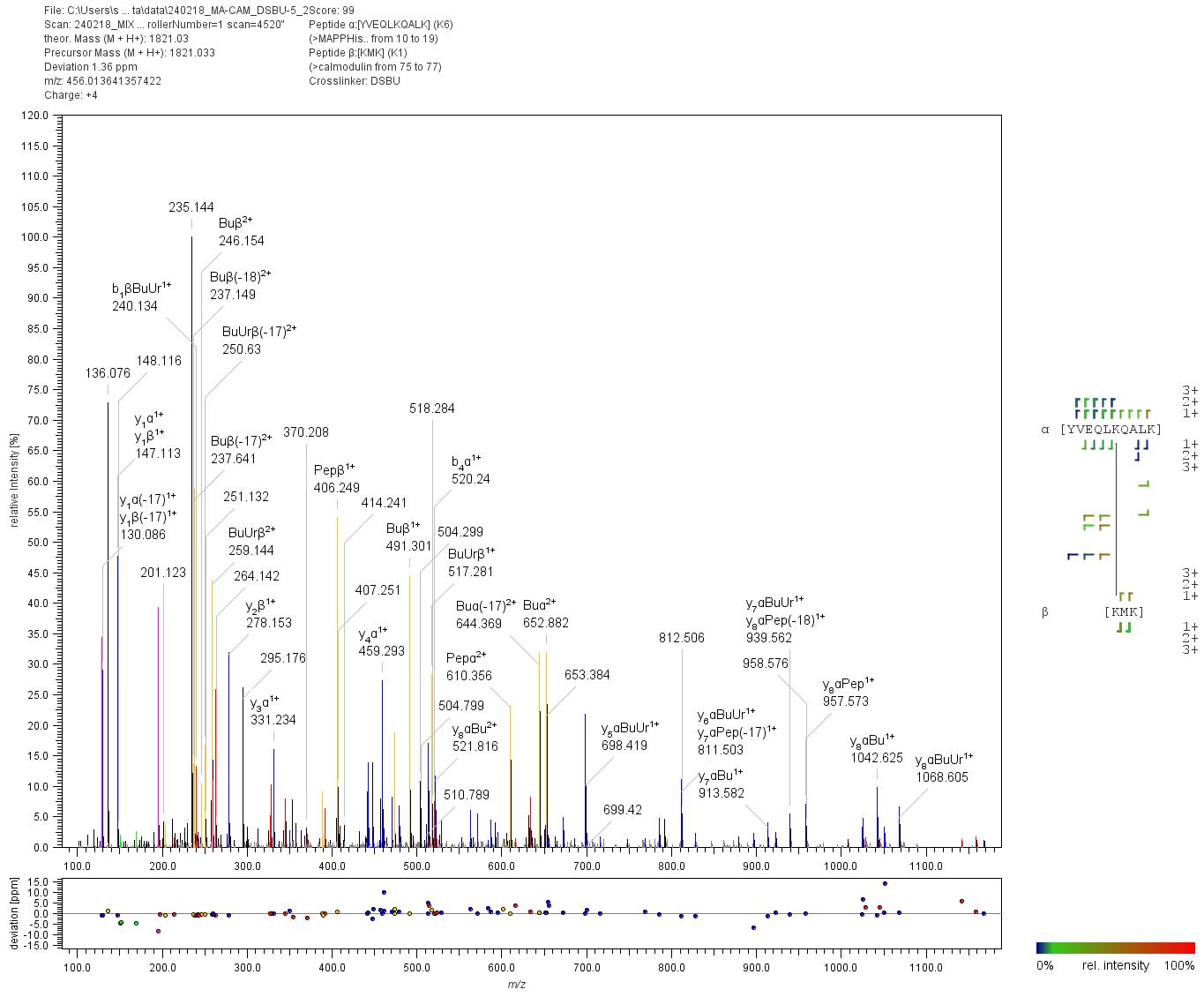


**DSBU: myrMAPP_K16-CaM_K75**

Linkage: [YVEQLKQALK] - [KMK] (K16-K75)

Peptide α: [YVEQLKQALK] (K6); (>myrMAPP from 11 to 20)

Peptide β: [KMK] (K1); (>CaM from 75 to 77)


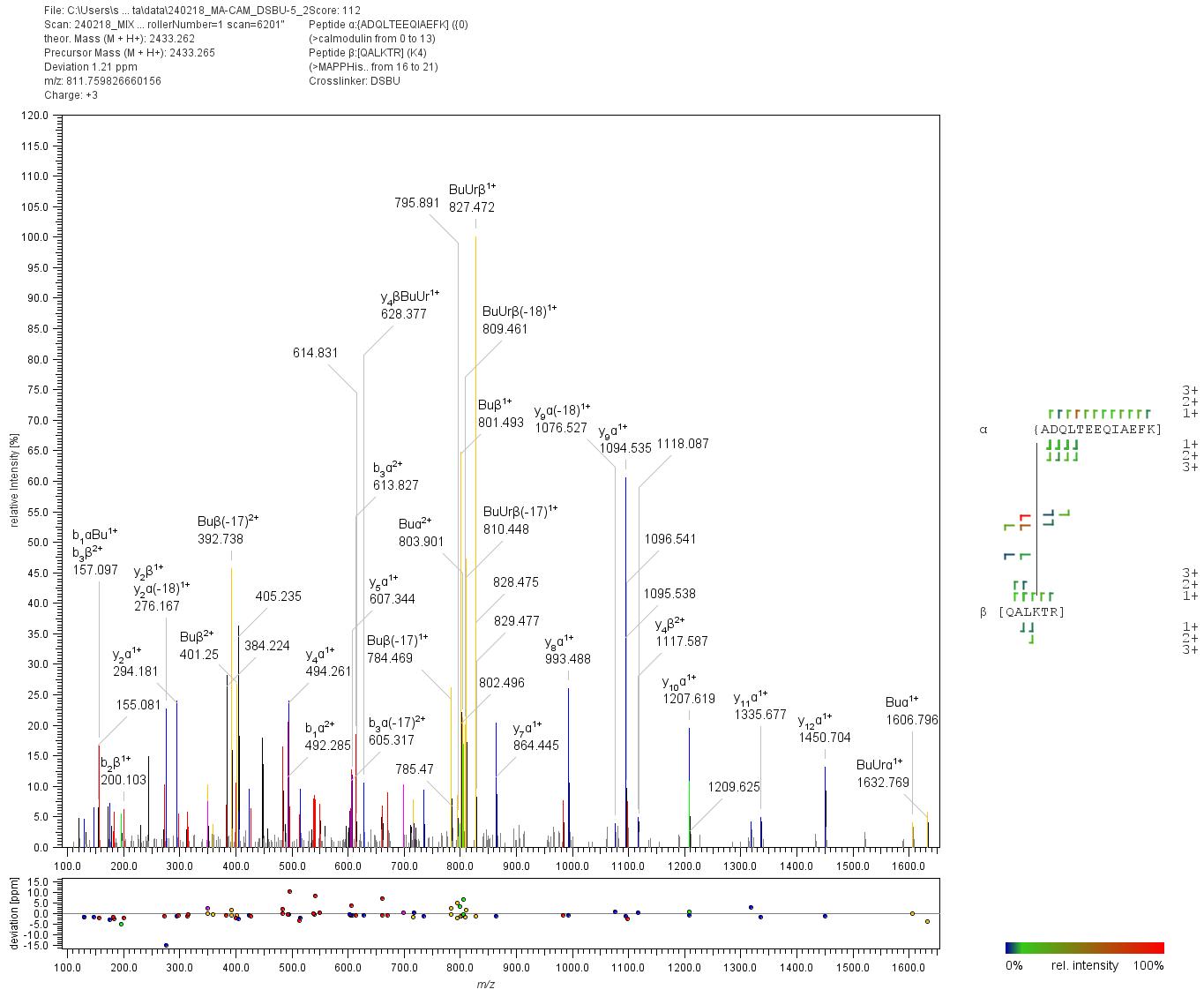


**DSBU: myrMAPP_K20-CaM_A1**

Linkage: {ADQLTEEQIAEFK] - [QALKTR] {0-K20)

Peptide α: {ADQLTEEQIAEFK] {0}; (> CaM from 0 to 13)

Peptide β: [QALKTR] (K4); (>myrMAPP from 17 to 22)


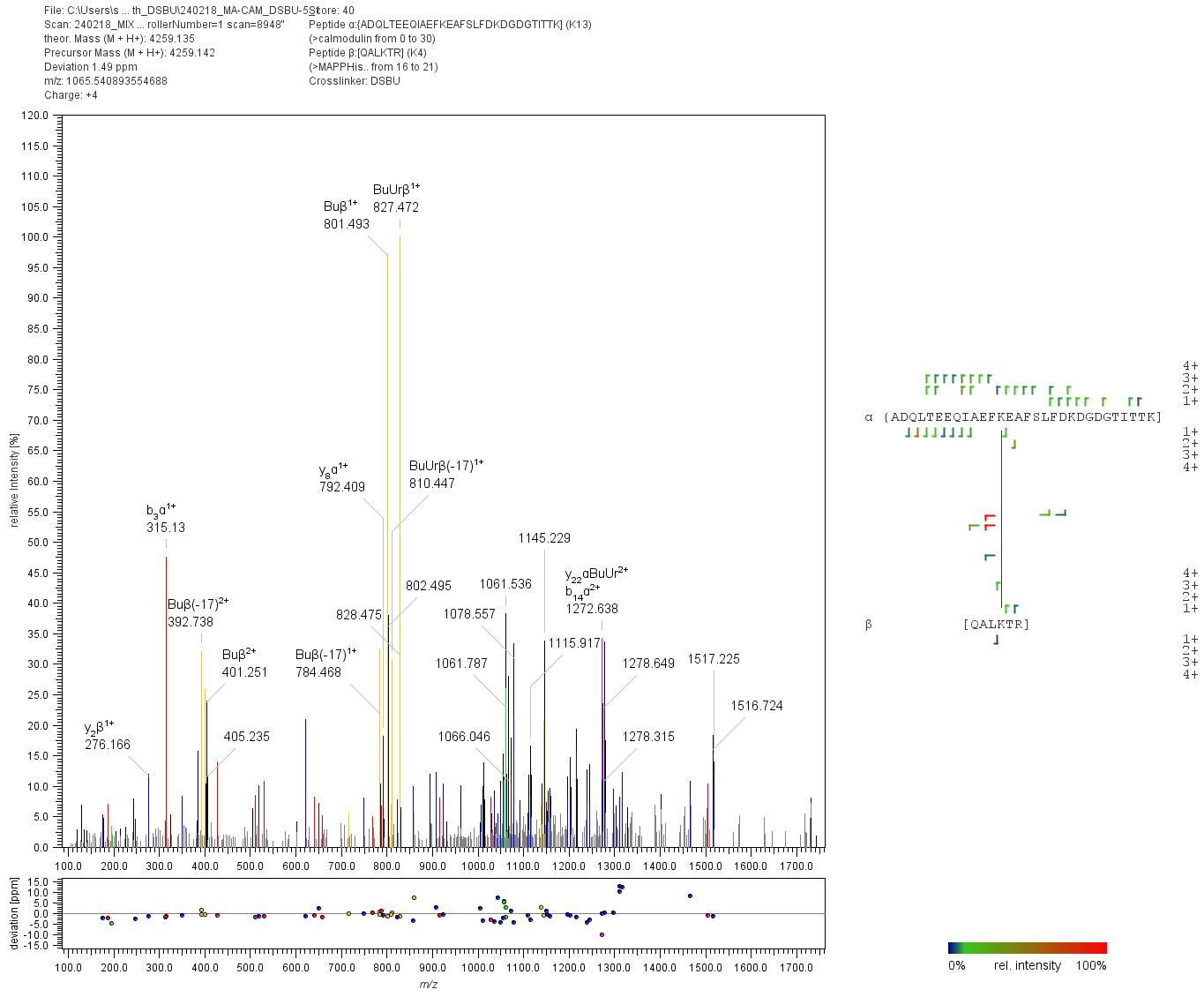


**DSBU: myrMAPP_K20-CaM_K13**

Linkage: {ADQLTEEQIAEFKEAFSLFDKDGDGTITTK] - [QALKTR] (K13-K20)

Peptide α: {ADQLTEEQIAEFKEAFSLFDKDGDGTITTK] (K13); (> CaM from 0 to 30)

Peptide β: [QALKTR] (K4); (>myrMAPP from 17 to 22)


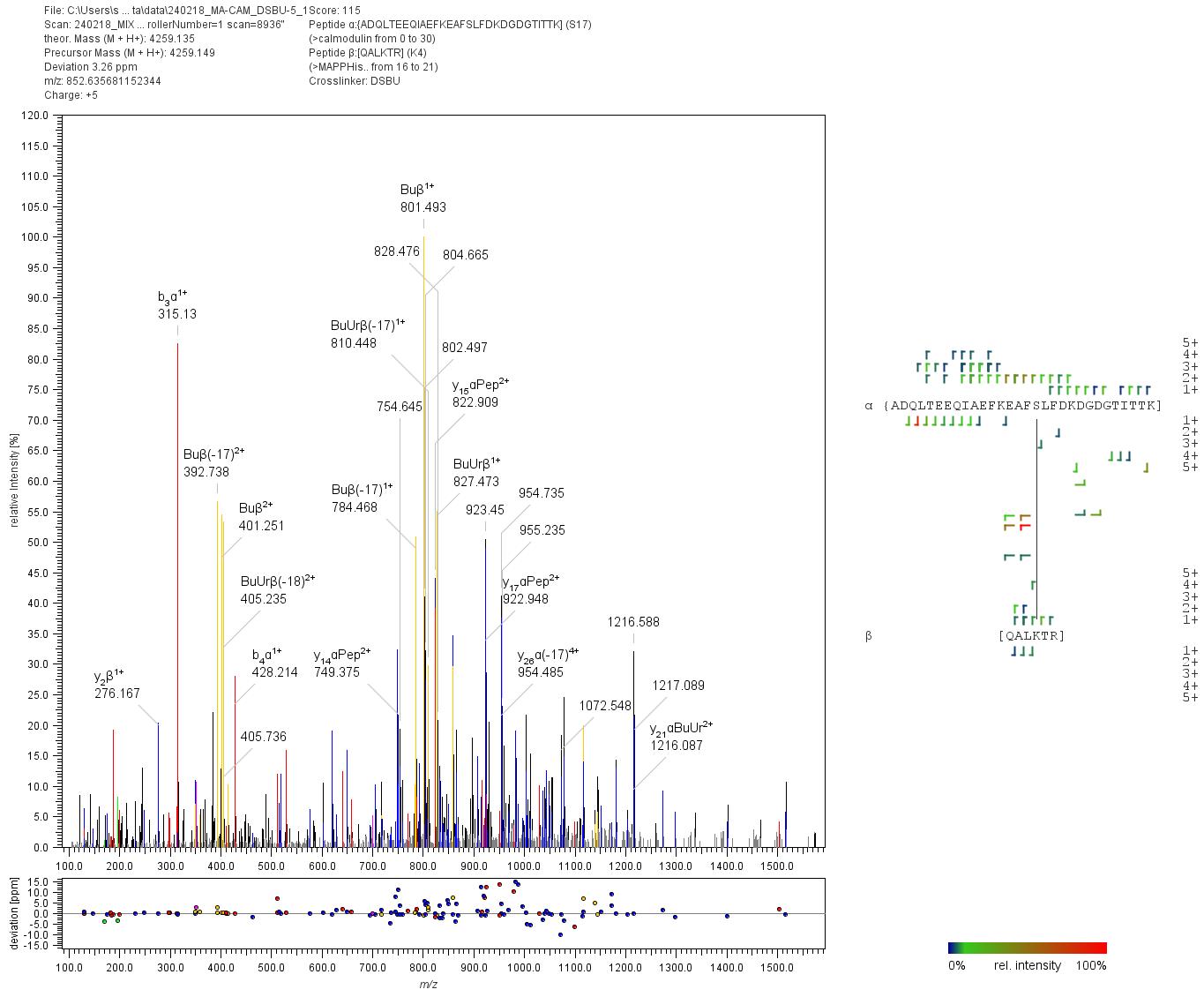


**DSBU: myrMAPP_K20-CaM_S17**

Linkage: {ADQLTEEQIAEFKEAFSLFDKDGDGTITTK] - [QALKTR] (S17-K20)

Peptide α: {ADQLTEEQIAEFKEAFSLFDKDGDGTITTK] (S17); (> CaM from 0 to 30)

Peptide β: [QALKTR] (K4); (>myrMAPP from 17 to 22)


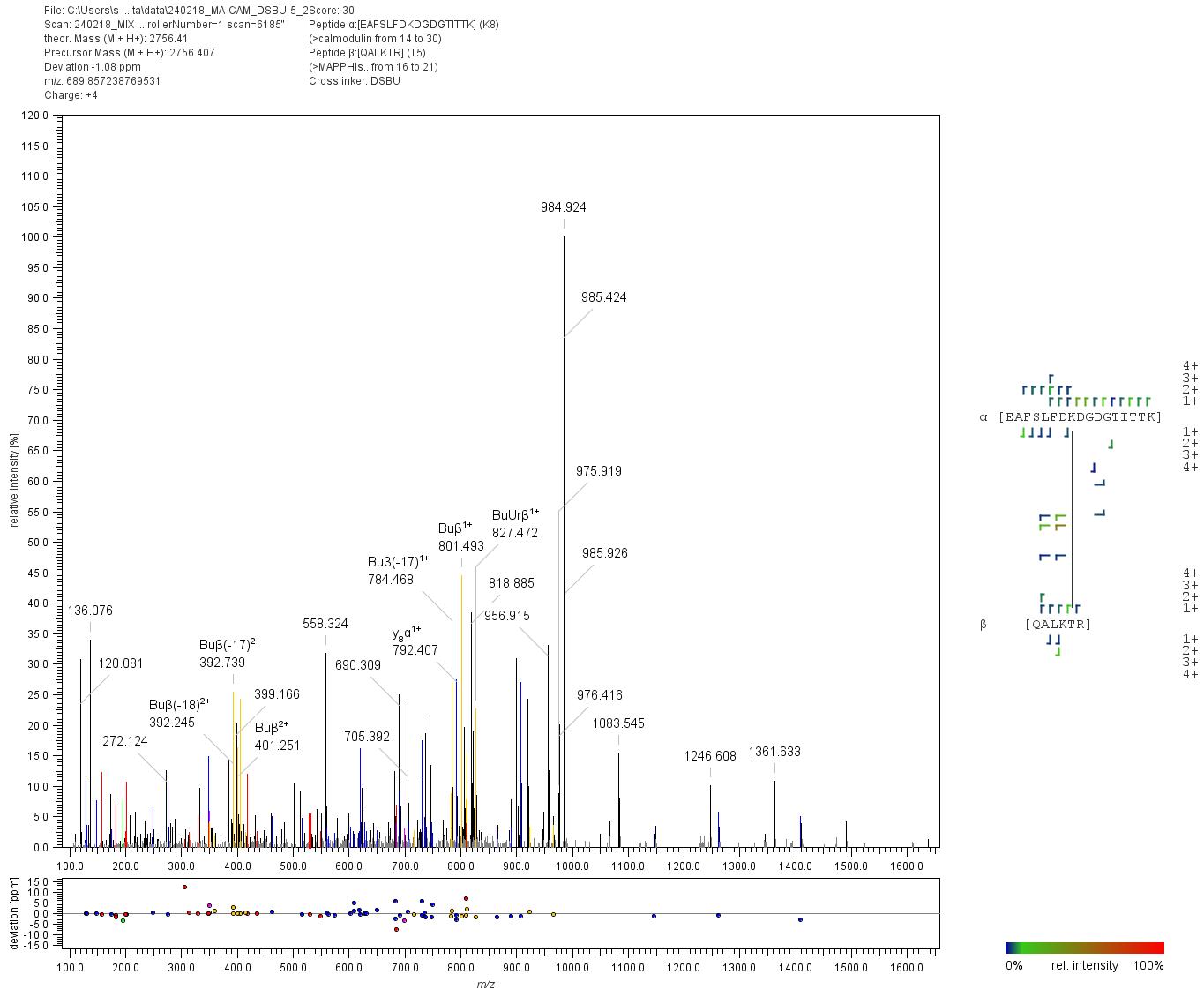


**DSBU: myrMAPP_T21-CaM_K21**

Linkage: [EAFSLFDKDGDGTITTK] - [QALKTR] (K21-T21)

Peptide α: [EAFSLFDKDGDGTITTK] (K8); (> CaM from 14 to 30)

Peptide β: [QALKTR] (T5); (>myrMAPP from 17 to 22)


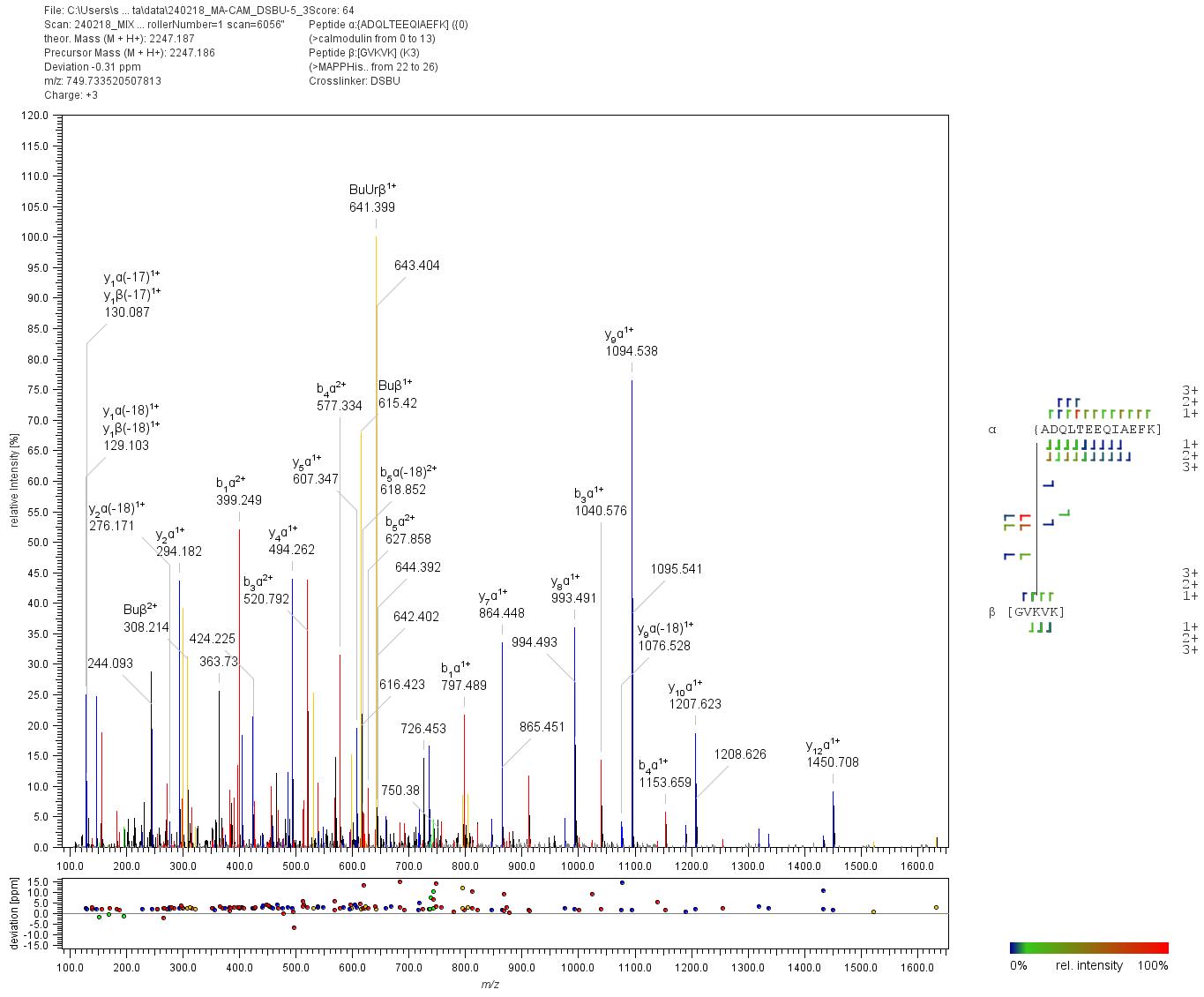


**DSBU: myrMAPP_K25-CaM_A1**

Linkage: {ADQLTEEQIAEFK] - [GVKVK] {0-25)

Peptide α: {ADQLTEEQIAEFK] {0}; (> CaM from 0 to 13)

Peptide β: [GVKVK] (K3); (>myrMAPP from 23 to 27)


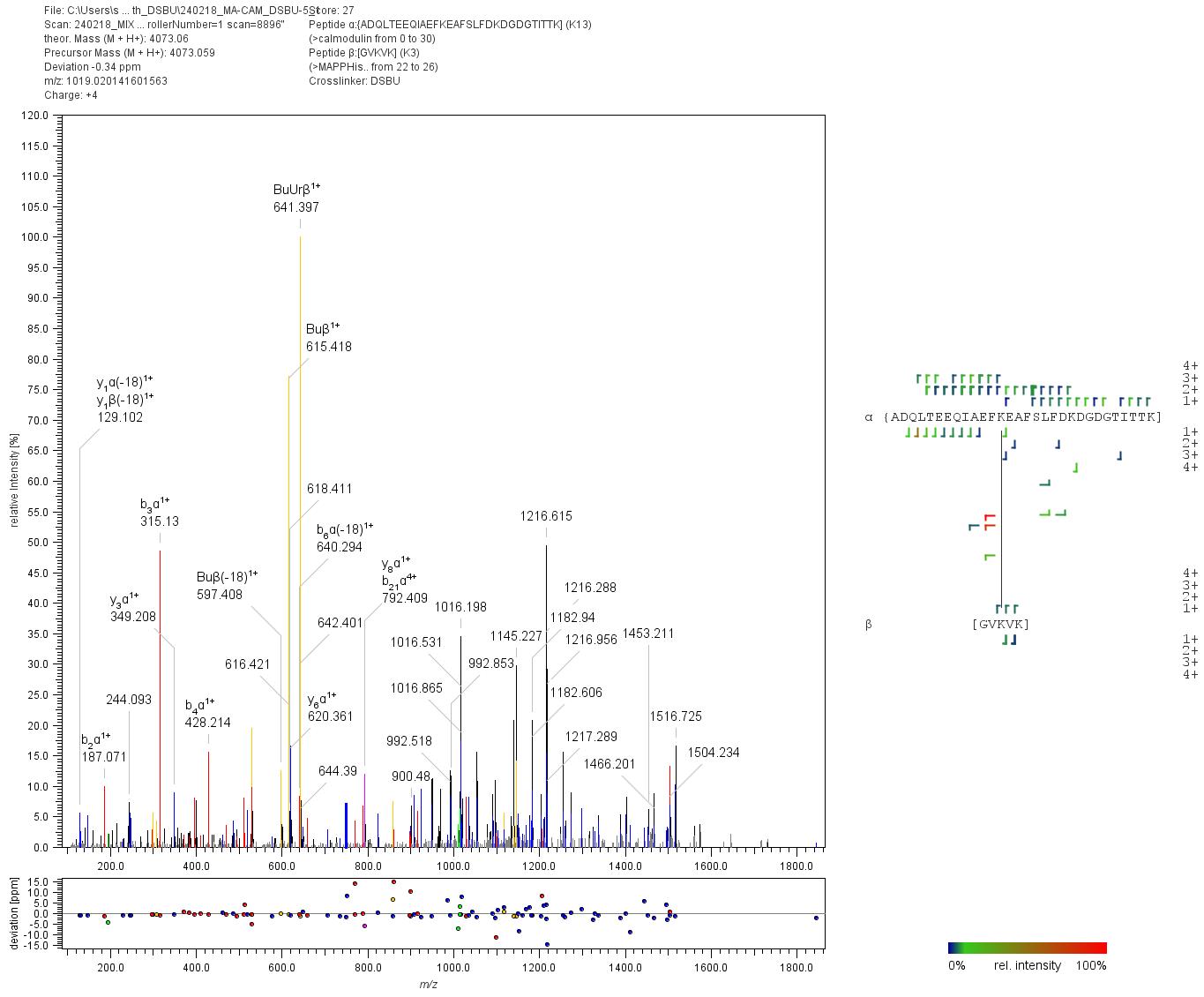


**DSBU: myrMAPP_K25-CaM_K13**

Linkage: {ADQLTEEQIAEFKEAFSLFDKDGDGTITTK] - [GVKVK] (K13-K25)

Peptide α: {ADQLTEEQIAEFKEAFSLFDKDGDGTITTK] (K13); (> CaM from 0 to 30)

Peptide β: [GVKVK] (K3); (>myrMAPP from 23 to 27)


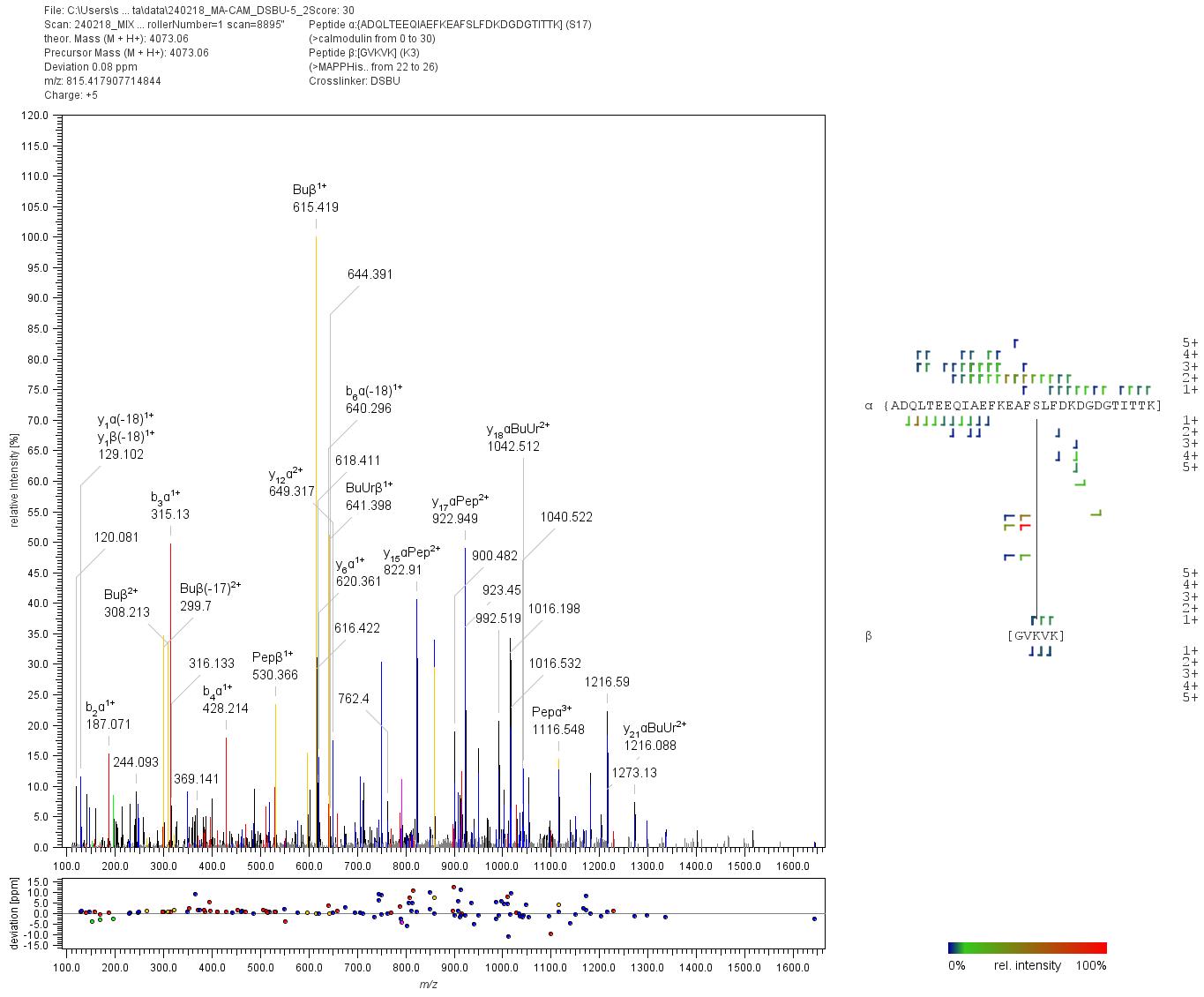


**DSBU: myrMAPP_K25-CaM_S17**

Linkage: {ADQLTEEQIAEFKEAFSLFDKDGDGTITTK] - [GVKVK] (S17-K25)

Peptide α: {ADQLTEEQIAEFKEAFSLFDKDGDGTITTK] (S17); (> CaM from 0 to 30)

Peptide β: [GVKVK] (K3); (>myrMAPP from 23 to 27)


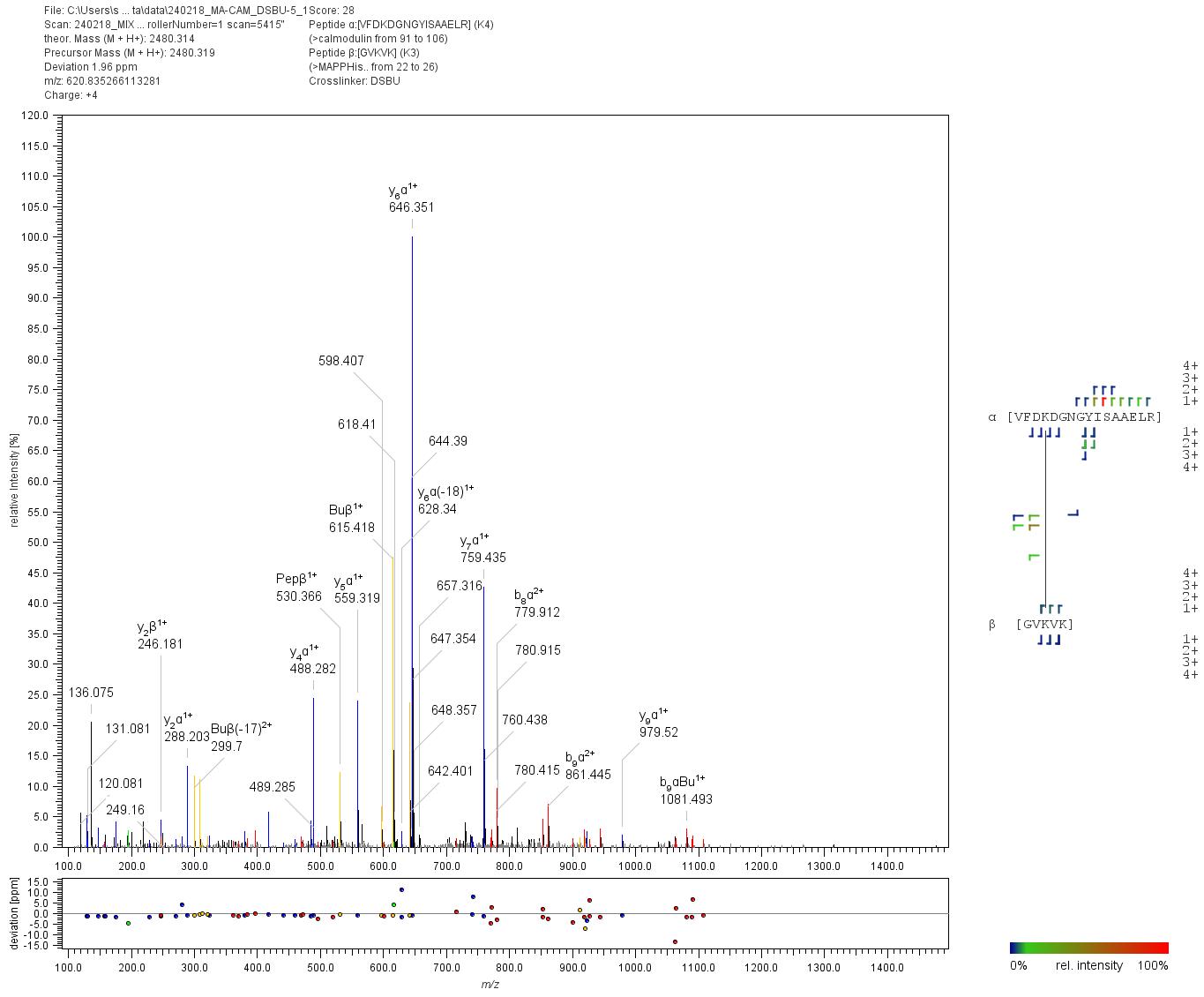


**DSBU: myrMAPP_K25-CaM_K94**

Linkage: [VFDKDGNGYISAAELR] - [GVKVK] (K94-K25)

Peptide α: [VFDKDGNGYISAAELR] (K4); (> CaM from 91 to 106)

Peptide β: [GVKVK] (K3); (>myrMAPP from 23 to 27)


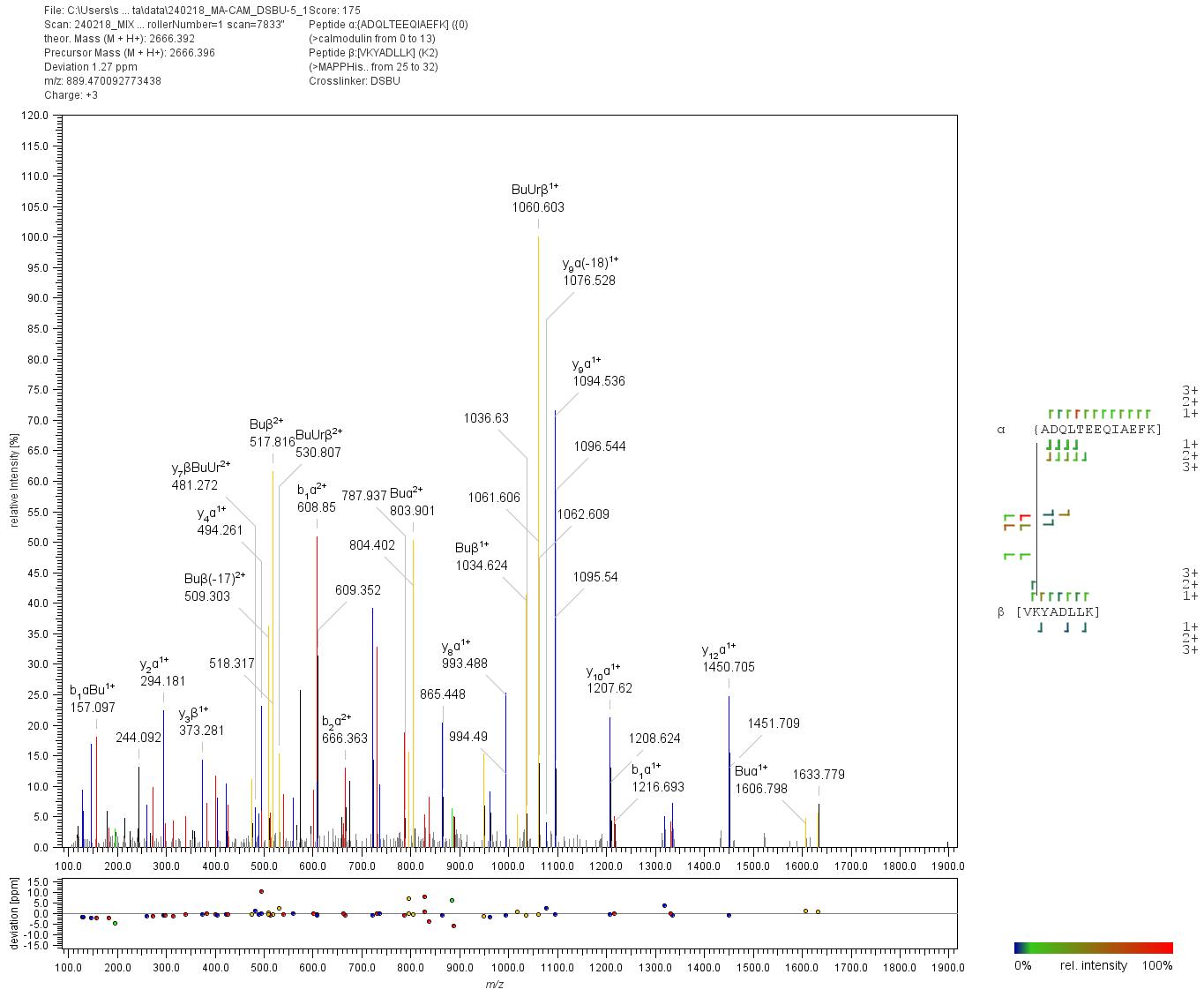


**DSBU: myrMAPP_K27-CaM_A1**

Linkage: {ADQLTEEQIAEFK] - [VKYADLLK] {0-K27)

Peptide α: {ADQLTEEQIAEFK] {0}; (> CaM from 0 to 13)

Peptide β: [VKYADLLK] (K2); (>myrMAPP from 26 to 33)


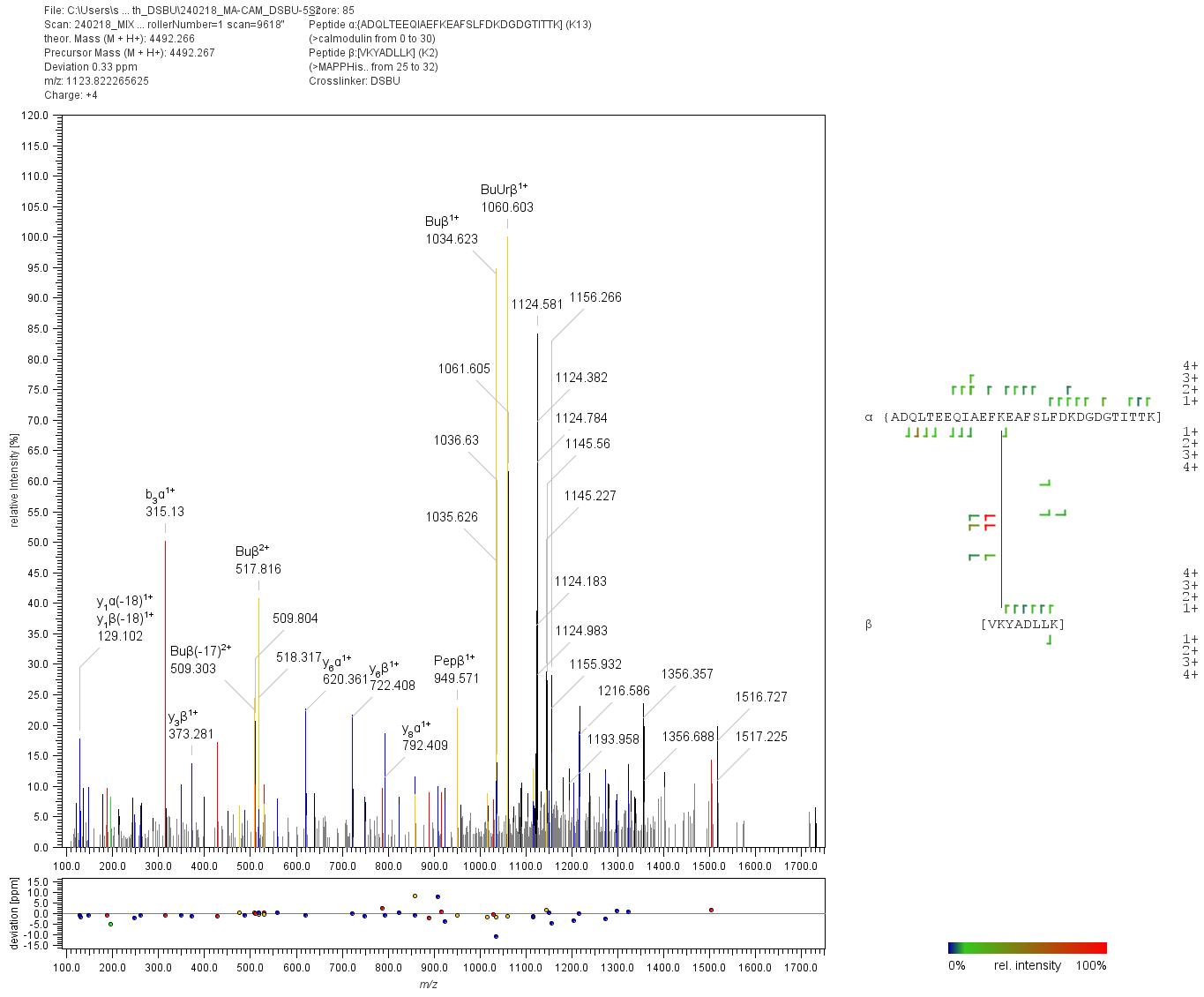


**DSBU: myrMAPP_K27-CaM_K13**

Linkage: {ADQLTEEQIAEFKEAFSLFDKDGDGTITTK] - [VKYADLLK] (K13-K27)

Peptide α: {ADQLTEEQIAEFKEAFSLFDKDGDGTITTK] (K13); (>CaM from 0 to 30)

Peptide β: [VKYADLLK] (K2); (>myrMAPP from 26 to 33)


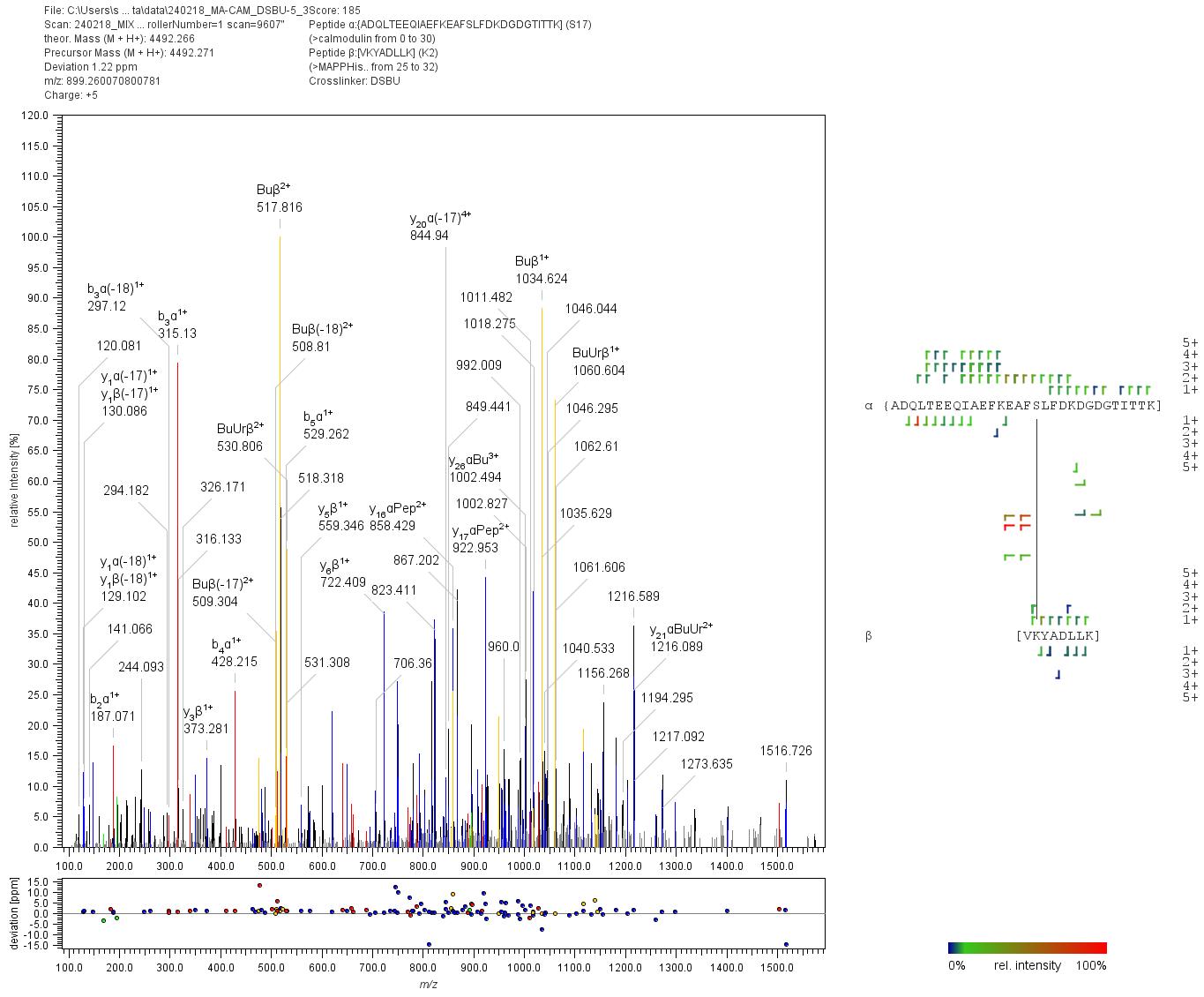


**DSBU: myrMAPP_K27-CaM_S17**

Linkage: {ADQLTEEQIAEFKEAFSLFDKDGDGTITTK] - [VKYADLLK] (S17-K27)

Peptide α: {ADQLTEEQIAEFKEAFSLFDKDGDGTITTK] (S17); (> CaM from 0 to 30)

Peptide β: [VKYADLLK] (K2); (>myrMAPP from 26 to 33)

**DSBU: myrMAPP_K27-CaM_T26**

Linkage: {ADQLTEEQIAEFKEAFSLFDKDGDGTITTK] - [VKYADLLK] (T26-K27)

Peptide α: {ADQLTEEQIAEFKEAFSLFDKDGDGTITTK] (T26); (>CaM from 0 to 30)

Peptide β: [VKYADLLK] (K2); (>myrMAPP from 26 to 33)


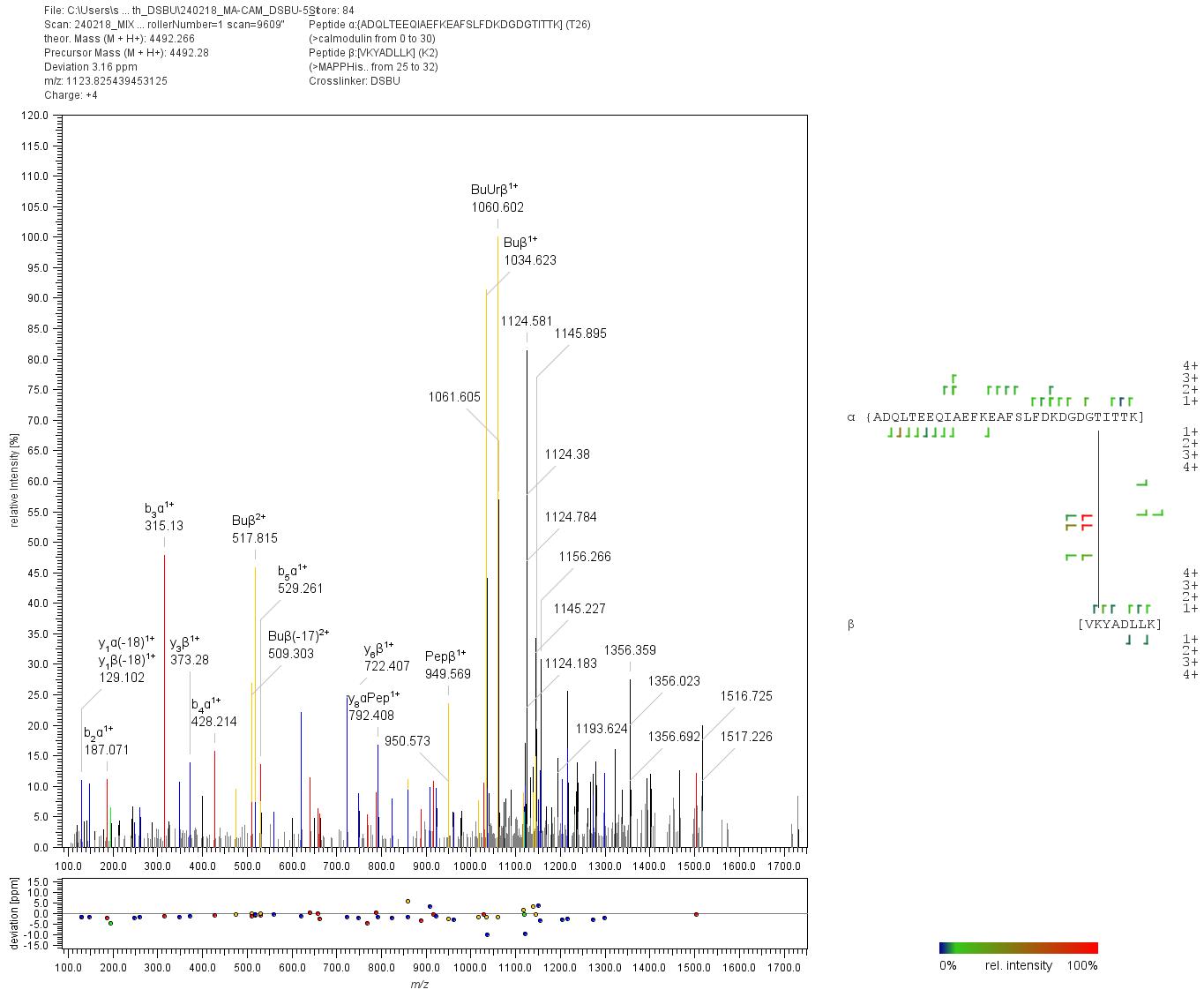

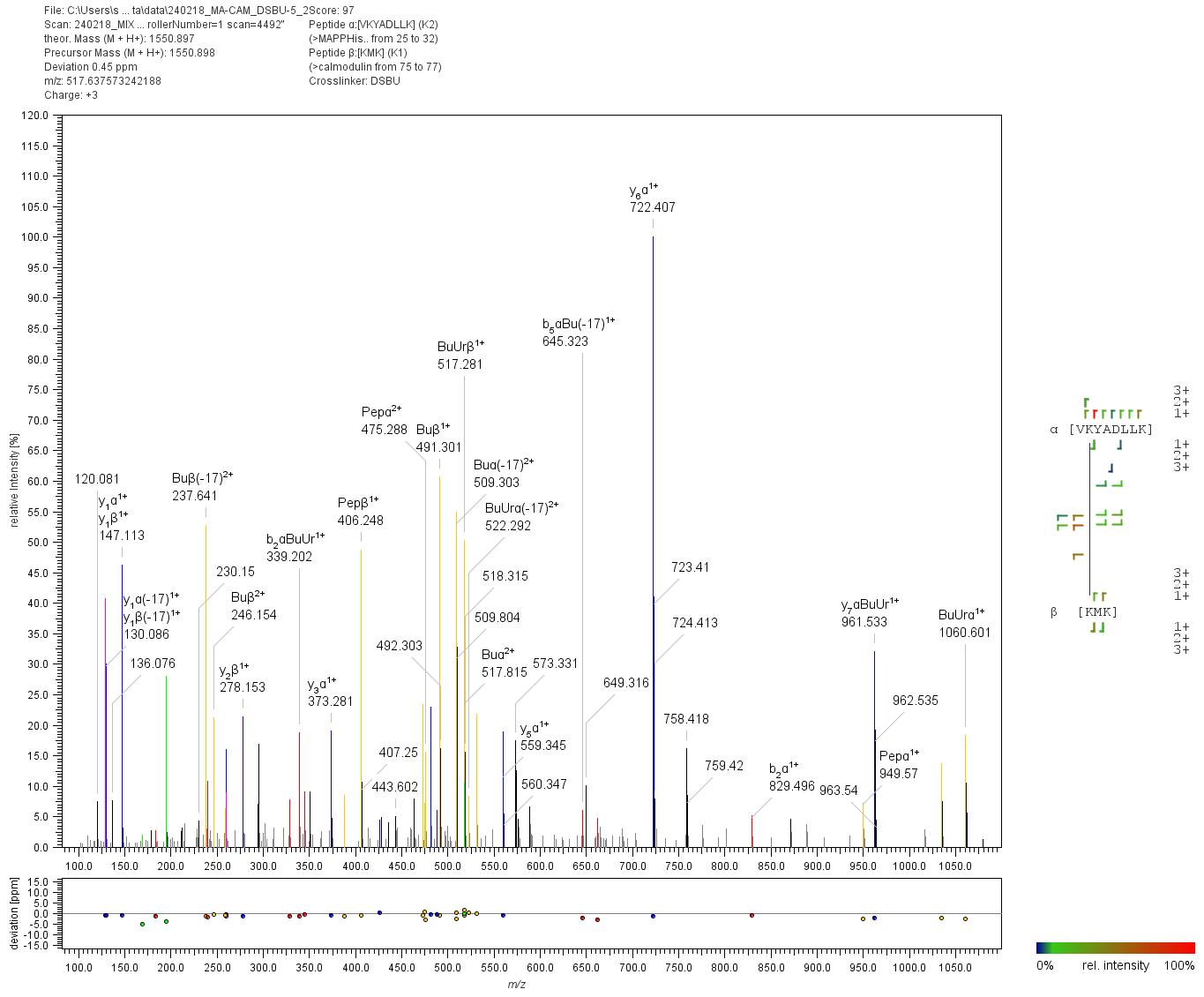


**DSBU: myrMAPP_K27-CaM_K75**

Linkage: [VKYADLLK] - [KMK] (K27-K75)

Peptide α: [VKYADLLK] (K2); (>myrMAPP from 26 to 33)

Peptide β: [KMK] (K1); (>CaM from 75 to 77)


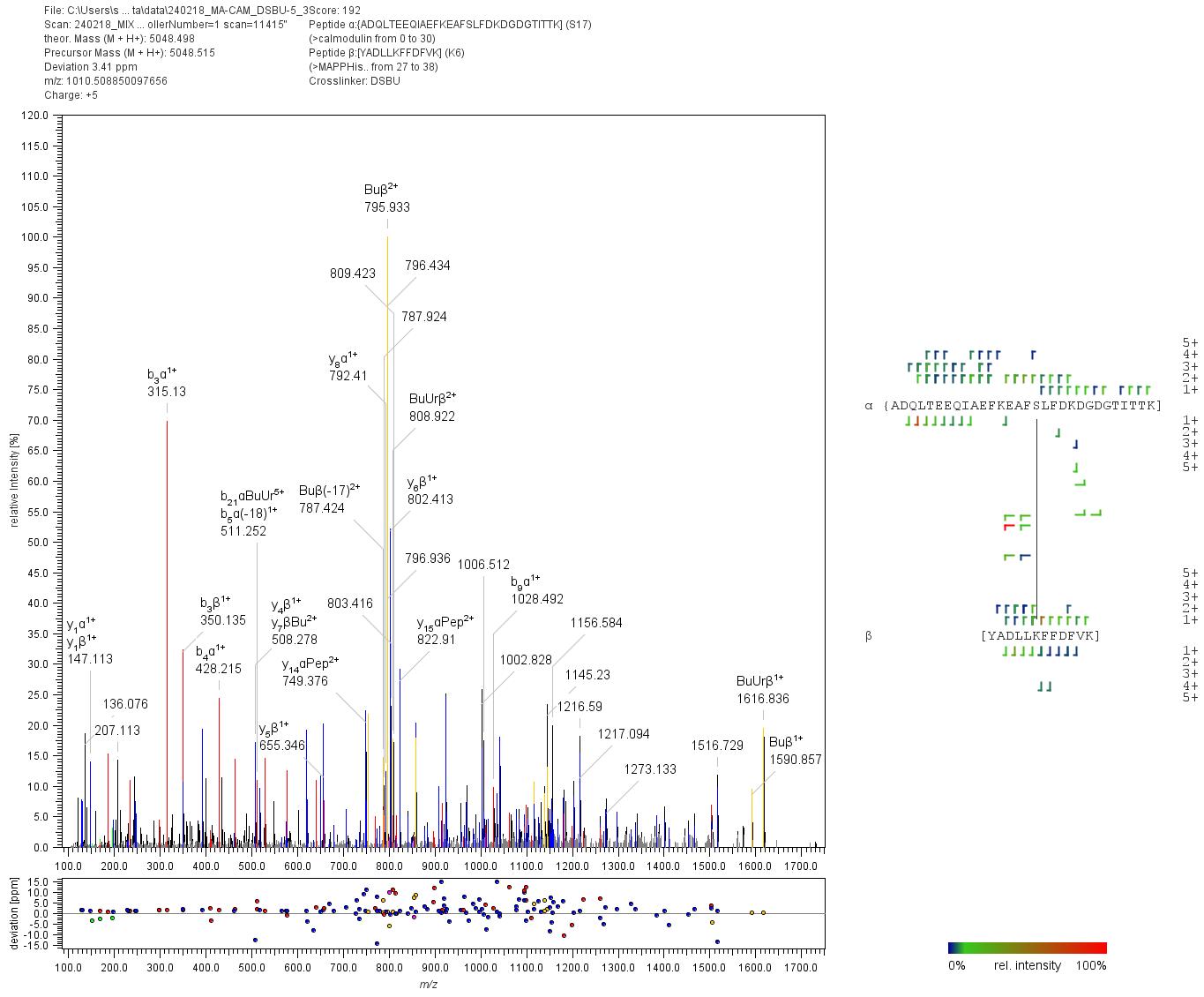


**DSBU: myrMAPP_K33-CaM_S17**

Linkage: {ADQLTEEQIAEFKEAFSLFDKDGDGTITTK] - [YADLLKFFDFVK] (S17-K33)

Peptide α: {ADQLTEEQIAEFKEAFSLFDKDGDGTITTK] (S17); (> CaM from 0 to 30)

Peptide β: [YADLLKFFDFVK] (K6); (>myrMAPP from 28 to 39)


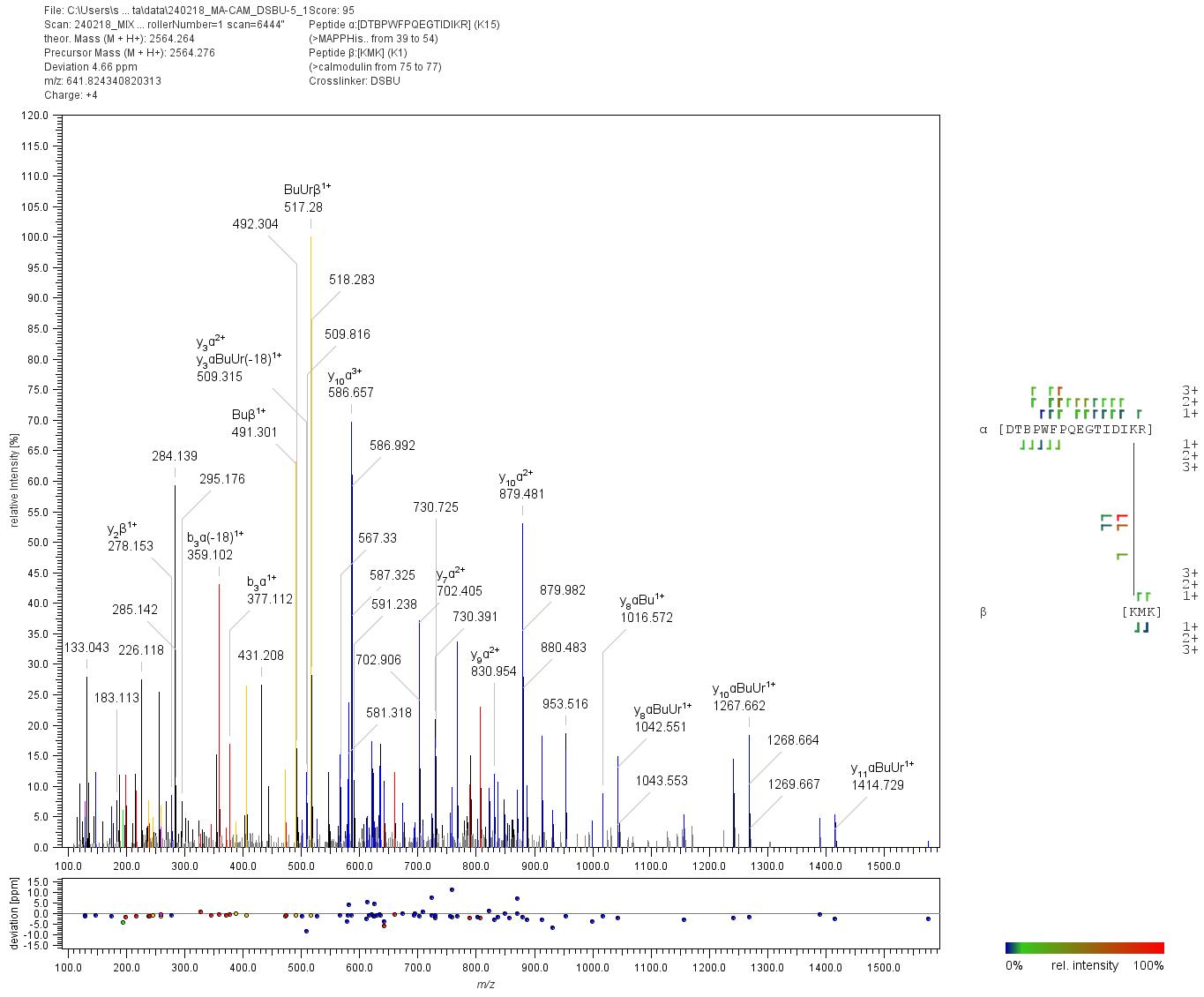


**DSBU: myrMAPP_K54-CaM_K75**

Linkage: [DTBPWFPQEGTIDIKR] - [KMK] (K54-K75)

Peptide α: [DTBPWFPQEGTIDIKR] (K15); (>myrMAPP from 40 to 55)

Peptide β: [KMK] (K1); (>CaM from 75 to 77)


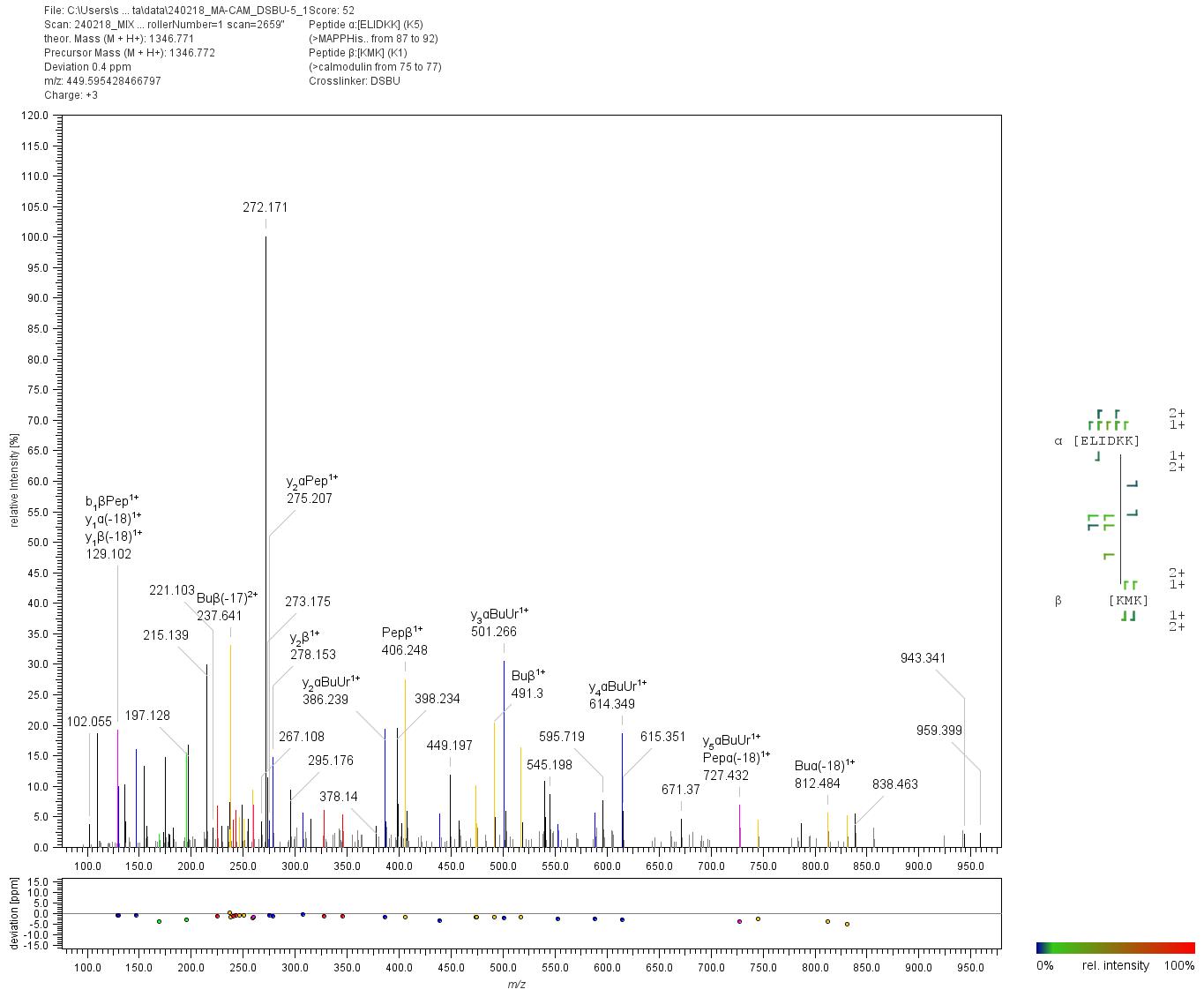


**DSBU: myrMAPP_K92-CaM_K75**

Linkage: [ELIDKK] - [KMK] (K92-K75)

Peptide α: [ELIDKK] (K5); (>myrMAPP from 88 to 93)

Peptide β: [KMK] (K1); (>CaM from 75 to 77)


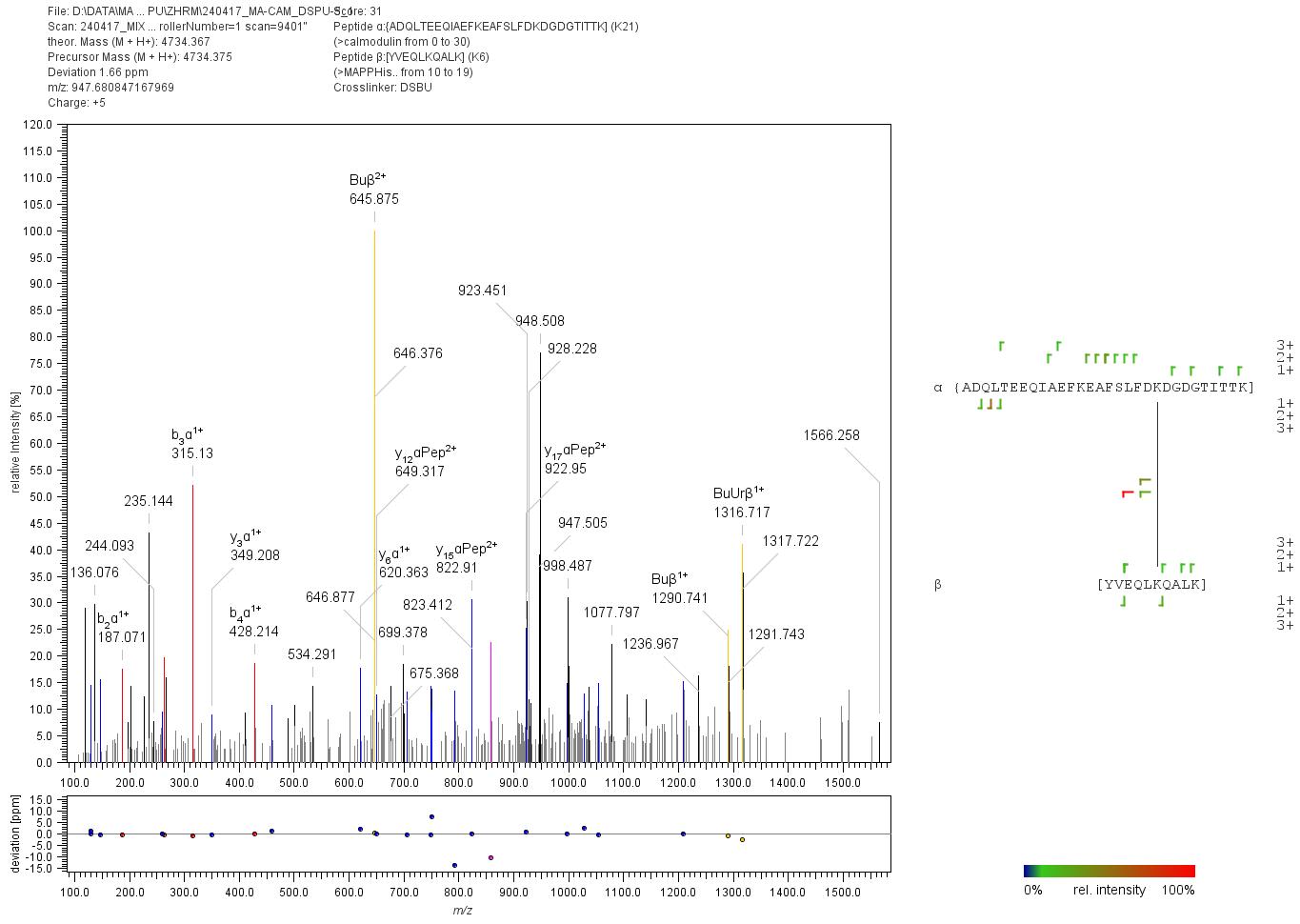


**DSPU: myrMAPP_K16-CaM_K21**

Linkage: {ADQLTEEQIAEFKEAFSLFDKDGDGTITTK] - [YVEQLKQALK] (K21-K16)

Peptide α: {ADQLTEEQIAEFKEAFSLFDKDGDGTITTK] (K21); (> CaM from 0 to 30)

Peptide β: [YVEQLKQALK] (K6); (>myrMAPP from 11 to 20)


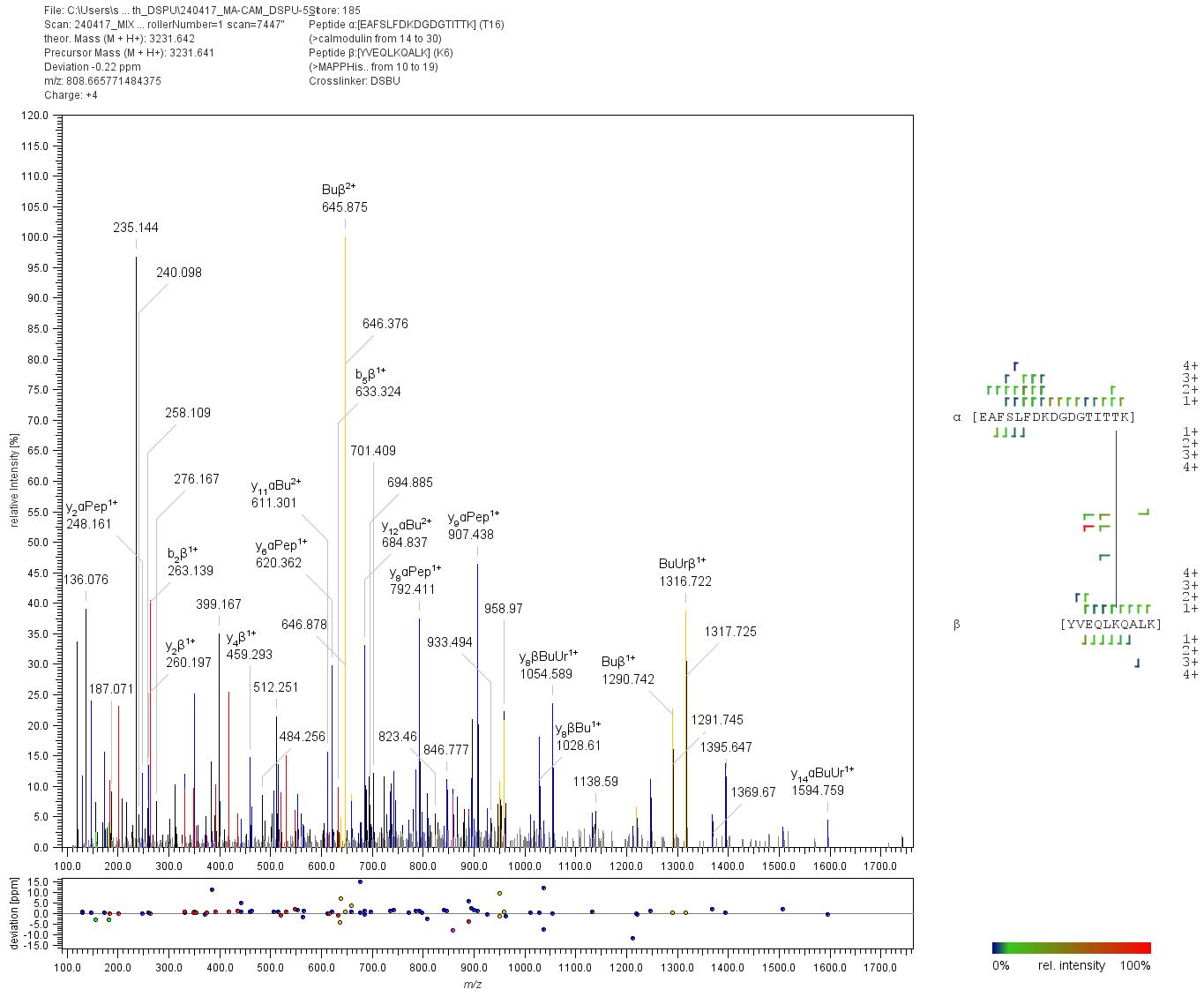


**DSPU: myrMAPP_K16-CaM_T29**

Linkage: [EAFSLFDKDGDGTITTK] - [YVEQLKQALK] (T29-K16)

Peptide α: [EAFSLFDKDGDGTITTK] (T16); (> CaM from 14 to 30)

Peptide β: [YVEQLKQALK] (K6); (>myrMAPP from 11 to 20)


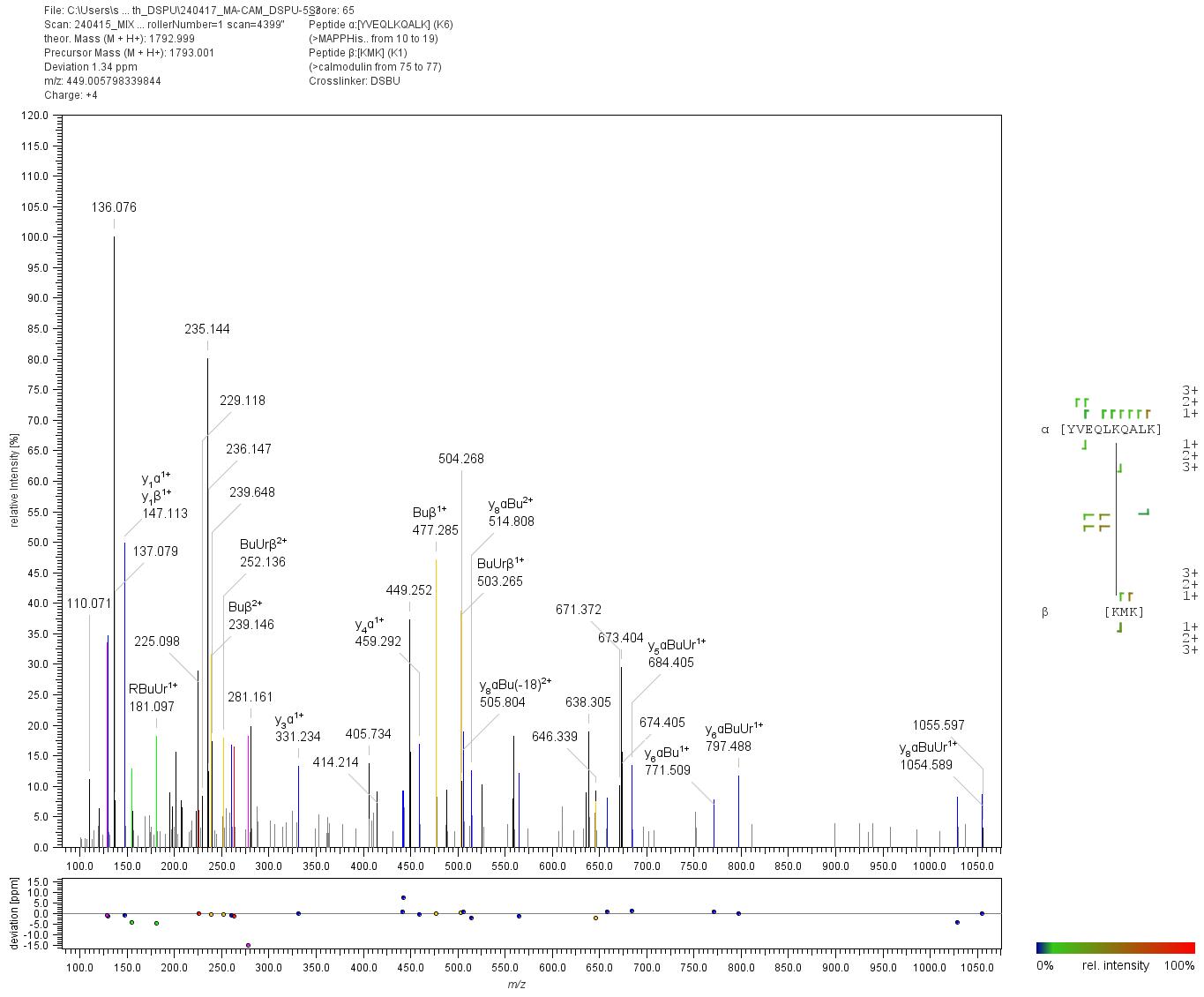


**DSPU: myrMAPP_K16-CaM_K75**

Linkage: [YVEQLKQALK] - [KMK] (K16-K75)

Peptide α: [YVEQLKQALK] (K6); (>myrMAPP from 11 to 20)

Peptide β: [KMK] (K1); (>CaM from 75 to 77)


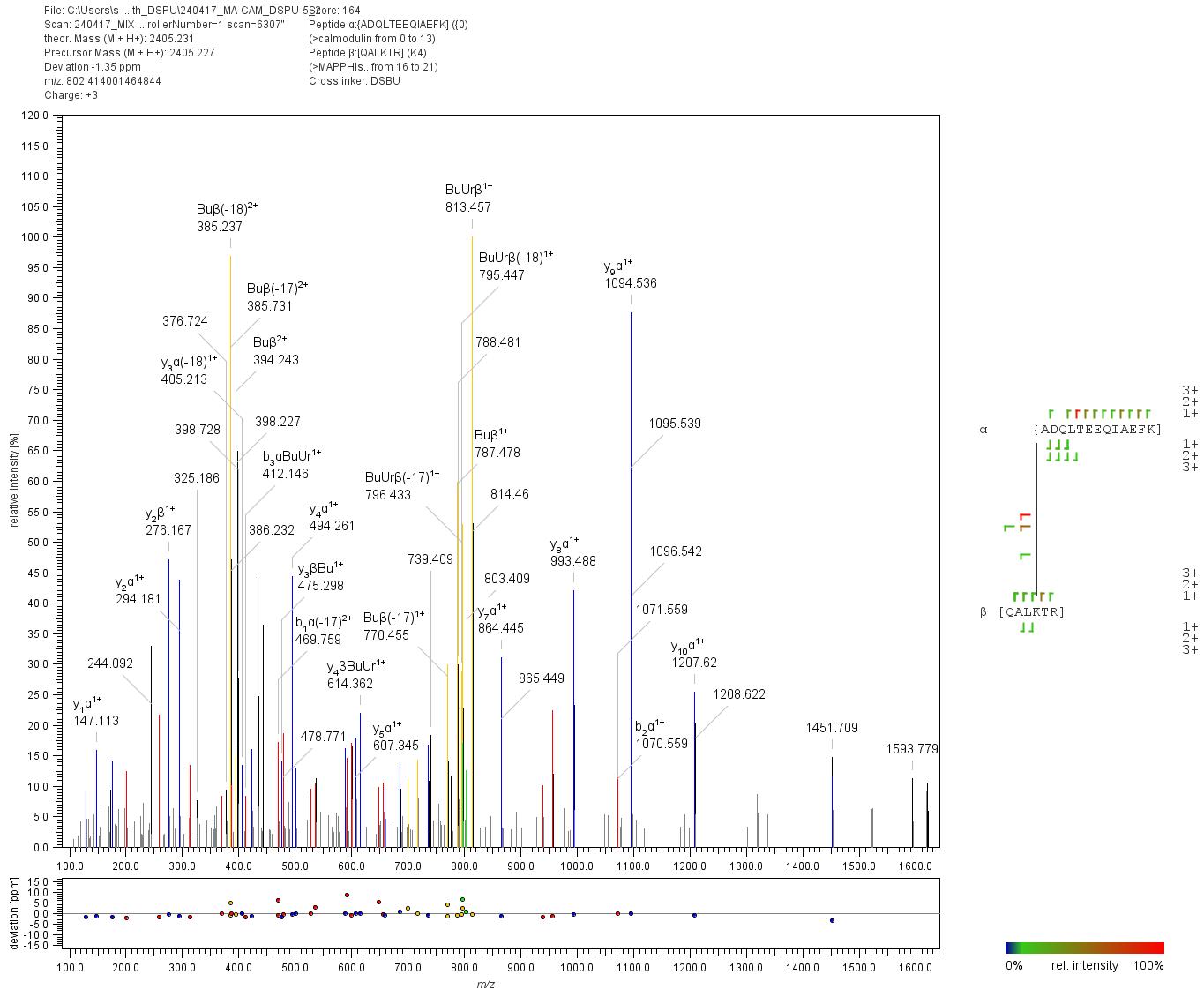


**DSPU: myrMAPP_K20-CaM_A1**

Linkage: {ADQLTEEQIAEFK] - [QALKTR] {0-K20)

Peptide α: {ADQLTEEQIAEFK] {0}; (> CaM from 0 to 13)

Peptide β: [QALKTR] (K4); (>myrMAPP from 17 to 22)


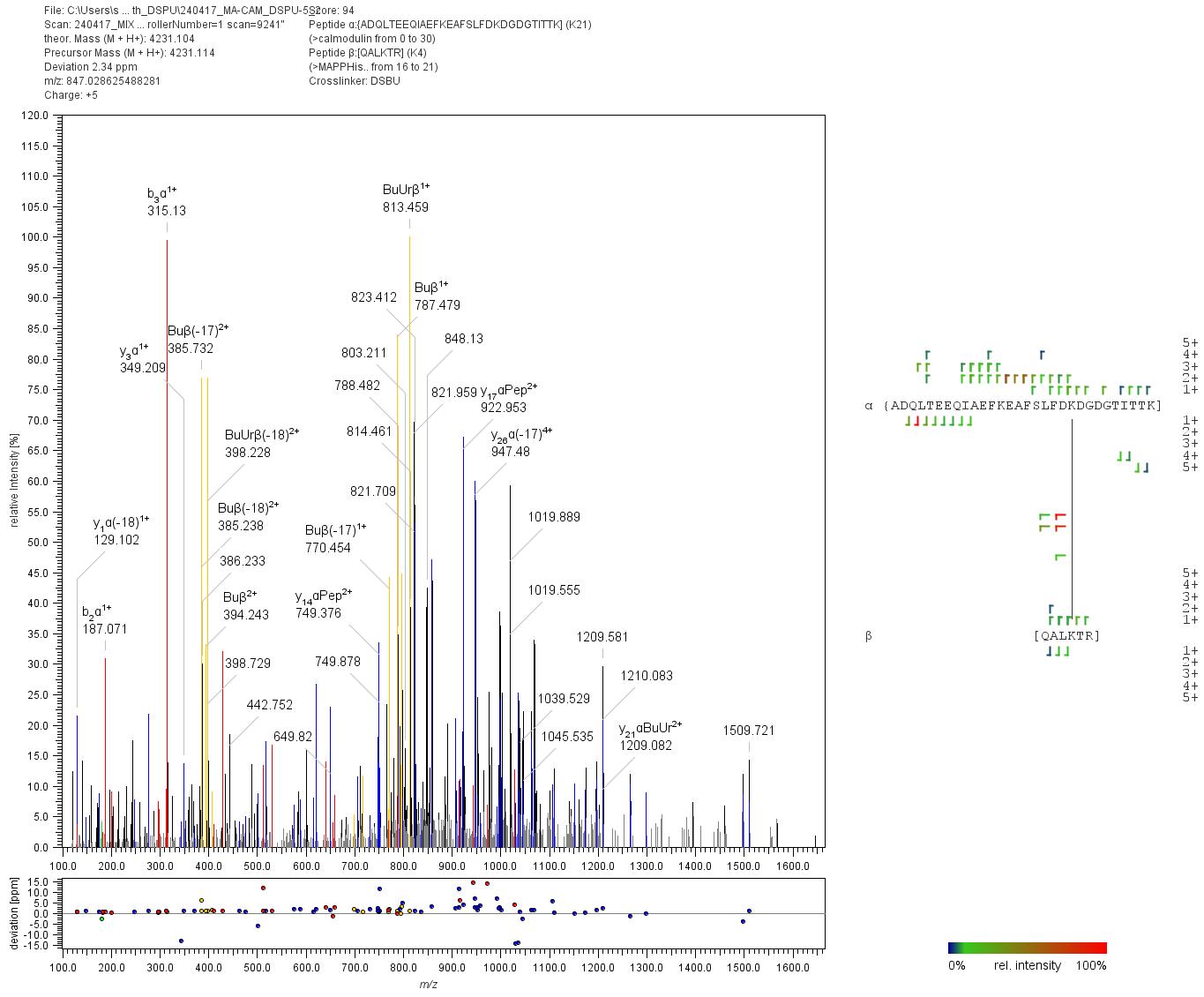


**DSPU: myrMAPP_K20-CaM_K21**

Linkage: {ADQLTEEQIAEFKEAFSLFDKDGDGTITTK] - [QALKTR] (K21-K20)

Peptide α: {ADQLTEEQIAEFKEAFSLFDKDGDGTITTK] (K21); (> CaM from 0 to 30)

Peptide β: [QALKTR] (K4); (>myrMAPP from 17 to 22)


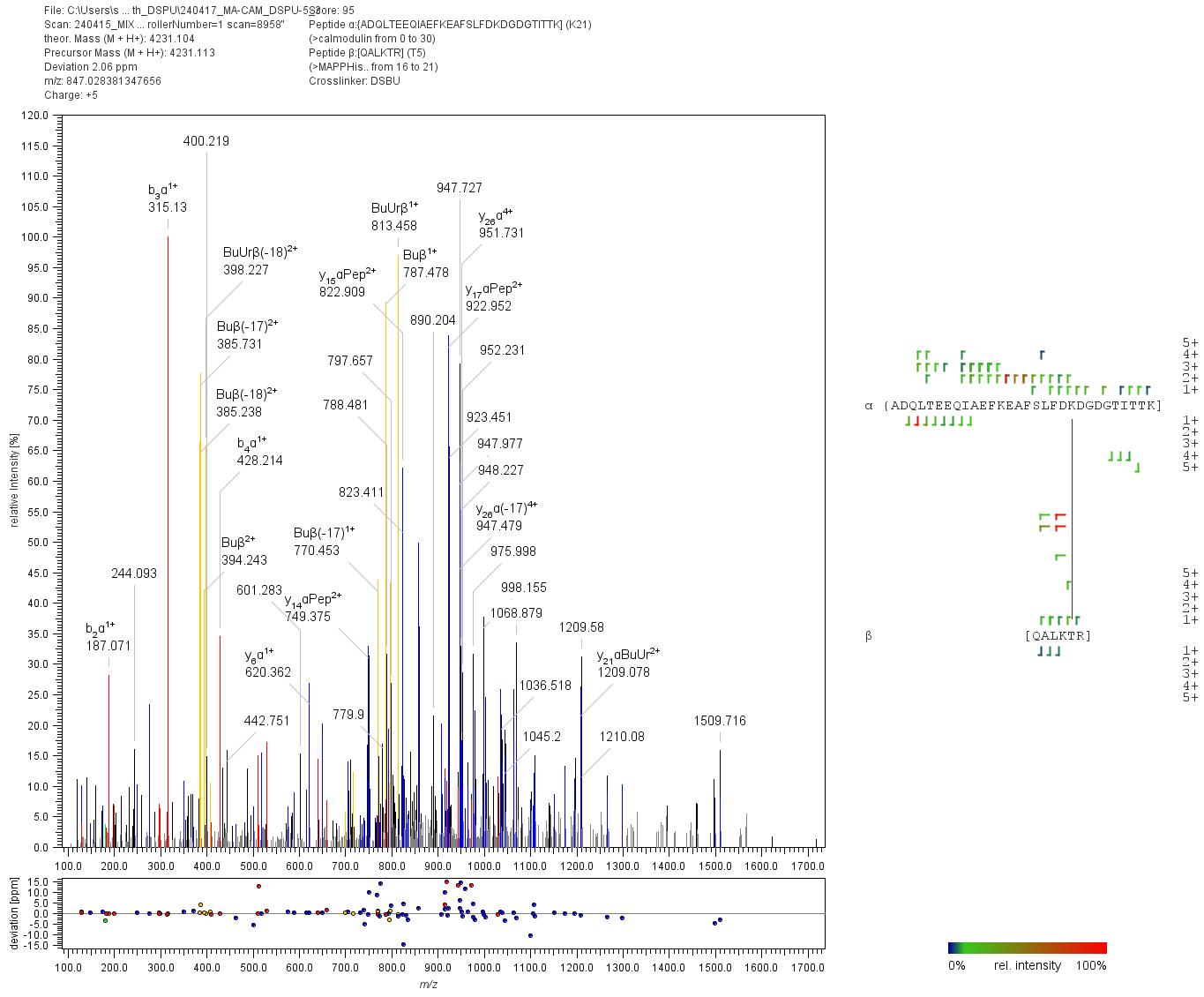


**DSPU: myrMAPP_T21-CaM_K21**

Linkage: {ADQLTEEQIAEFKEAFSLFDKDGDGTITTK] - [QALKTR] (K21-T21)

Peptide α: {ADQLTEEQIAEFKEAFSLFDKDGDGTITTK] (K21); (> CaM from 0 to 30)

Peptide β: [QALKTR] (T5); (>myrMAPP from 17 to 22)


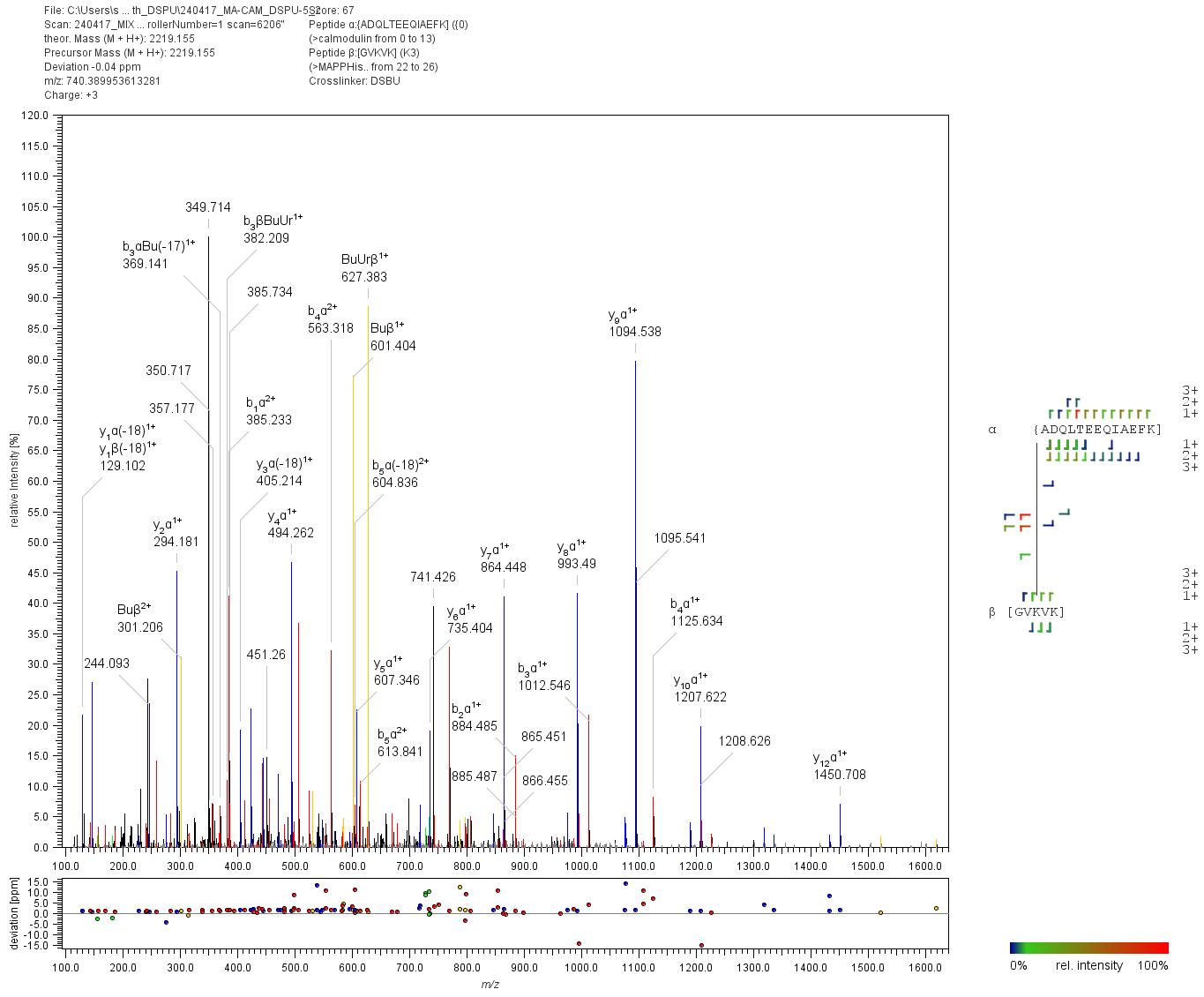


**DSPU: myrMAPP_K25-CaM_A1**

Linkage: {ADQLTEEQIAEFK] - [GVKVK] {0-K25)

Peptide α: {ADQLTEEQIAEFK] {0}; (> CaM from 0 to 13)

Peptide β: [GVKVK] (K3); (>myrMAPP from 23 to 27)


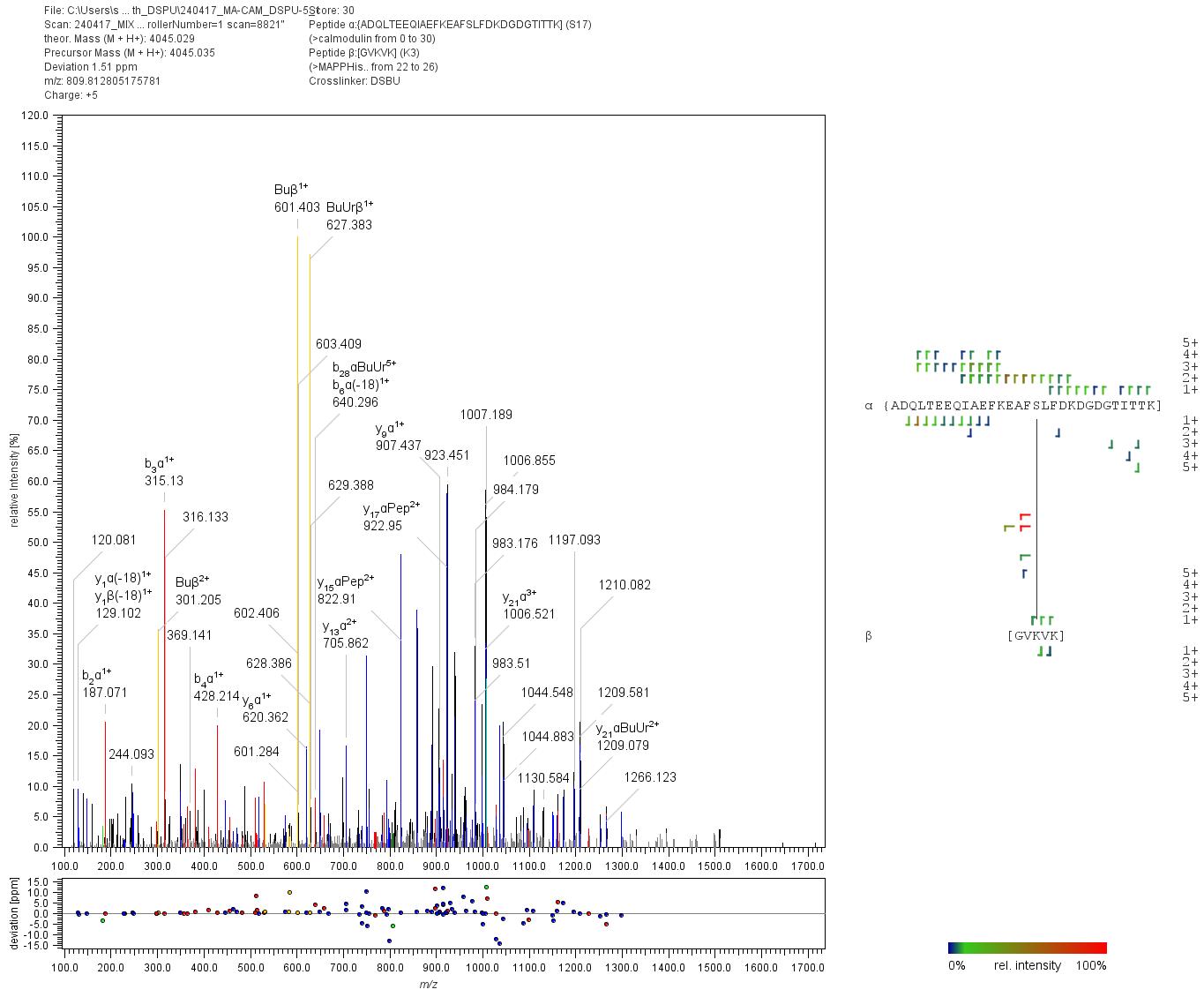


**DSPU: myrMAPP_K25-CaM_S17**

Linkage: {ADQLTEEQIAEFKEAFSLFDKDGDGTITTK] - [GVKVK] (S17-K25)

Peptide α: {ADQLTEEQIAEFKEAFSLFDKDGDGTITTK] (S17); (> CaM from 0 to 30)

Peptide β: [GVKVK] (K3); (>myrMAPP from 23 to 27)


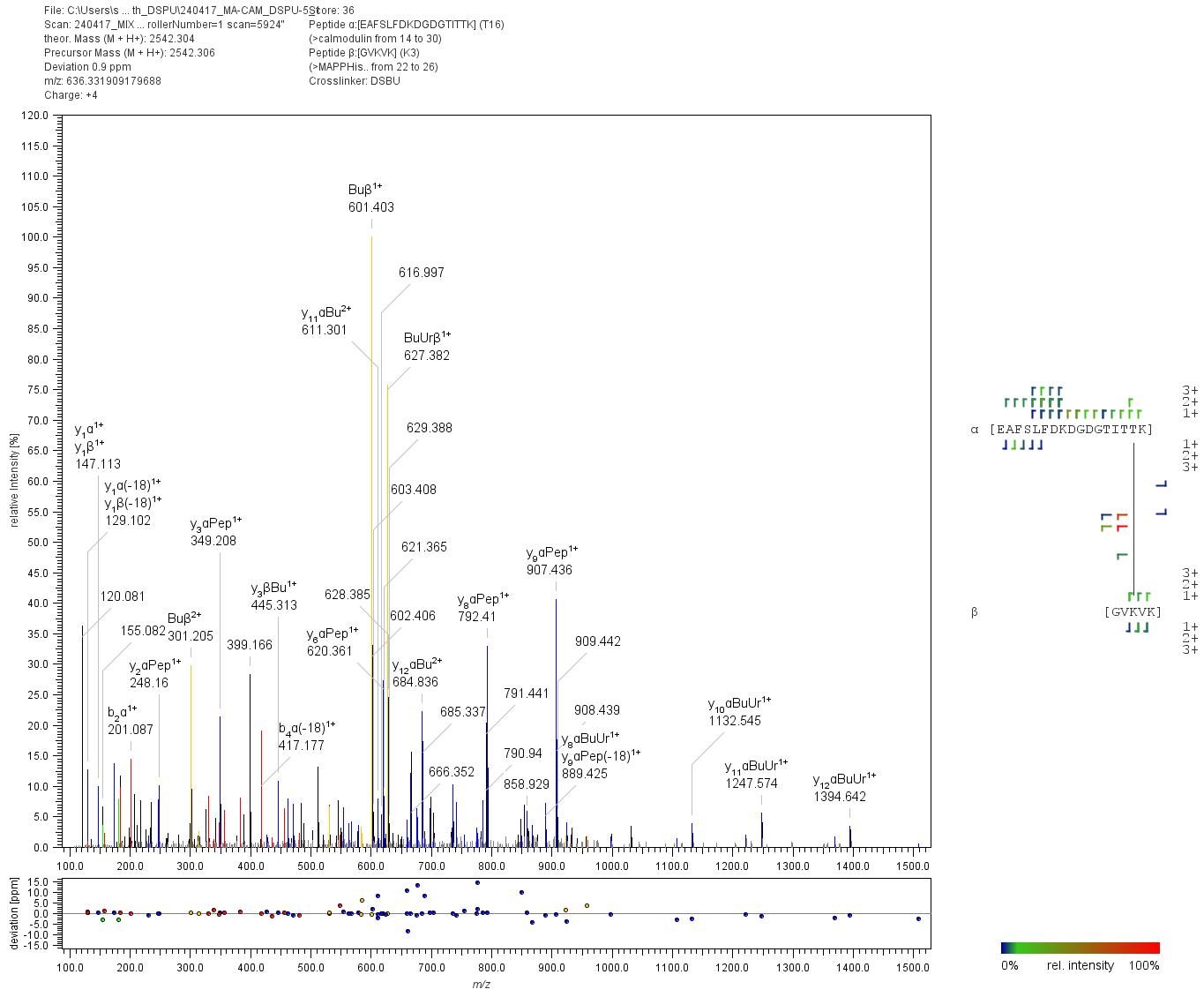


**DSPU: myrMAPP_K25-CaM_T29**

Linkage: [EAFSLFDKDGDGTITTK] - [GVKVK] (T29-K25)

Peptide α: [EAFSLFDKDGDGTITTK] (T16); (> CaM from 14 to 30)

Peptide β: [GVKVK] (K3); (>myrMAPP from 23 to 27)


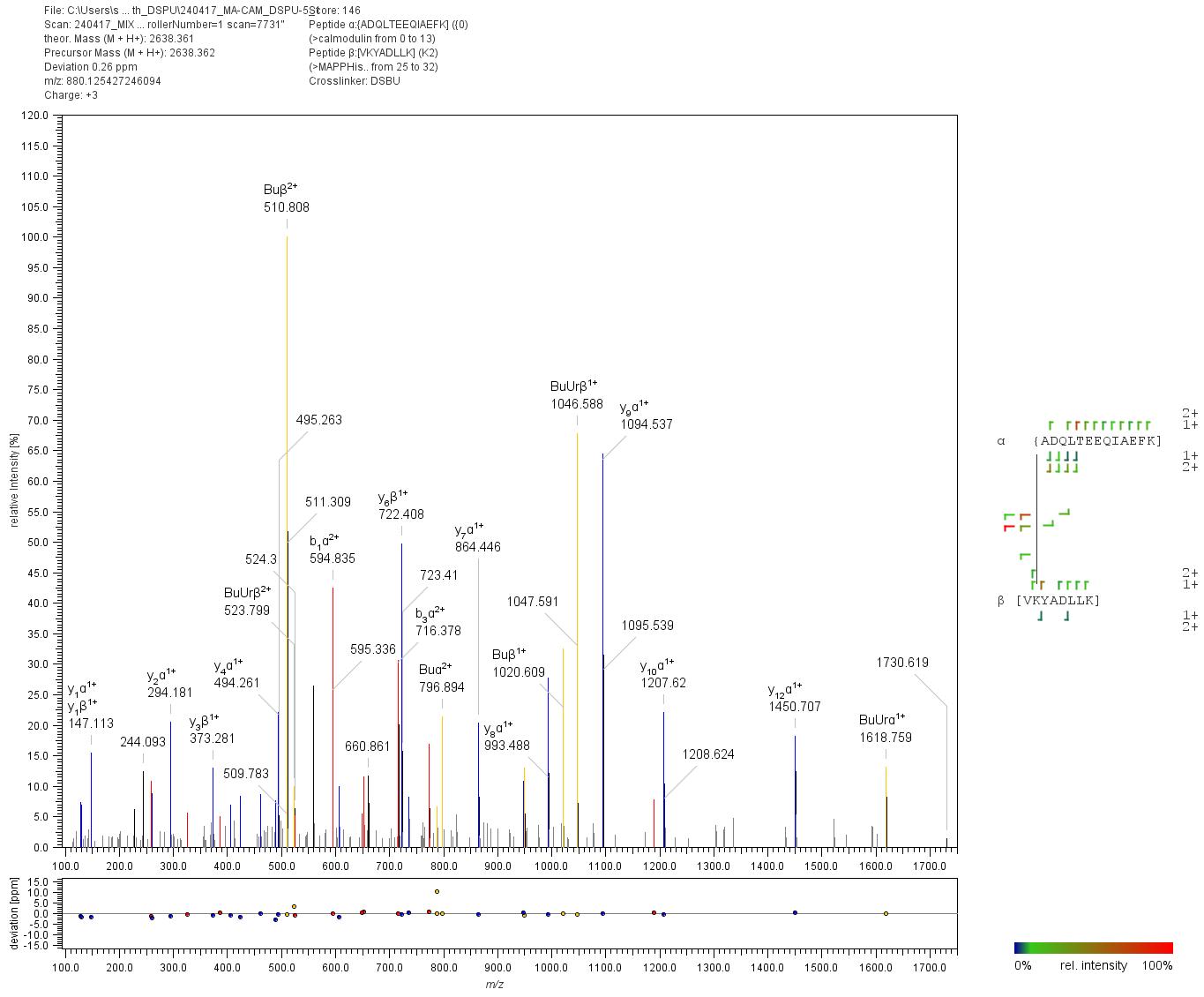


**DSPU: myrMAPP_K27-CaM_A1**

Linkage: {ADQLTEEQIAEFK] - [VKYADLLK] {0-K27)

Peptide α: {ADQLTEEQIAEFK] {0}; (> CaM from 0 to 13)

Peptide β: [VKYADLLK] (K2); (>myrMAPP from 26 to 33)

**DSPU: myrMAPP_K27-CaM_S17**

Linkage: {ADQLTEEQIAEFKEAFSLFDKDGDGTITTK] - [VKYADLLK] (S17-K27)

Peptide α: {ADQLTEEQIAEFKEAFSLFDKDGDGTITTK] (S17); (> CaM from 0 to 30)

Peptide β: [VKYADLLK] (K2); (>myrMAPP from 26 to 33)


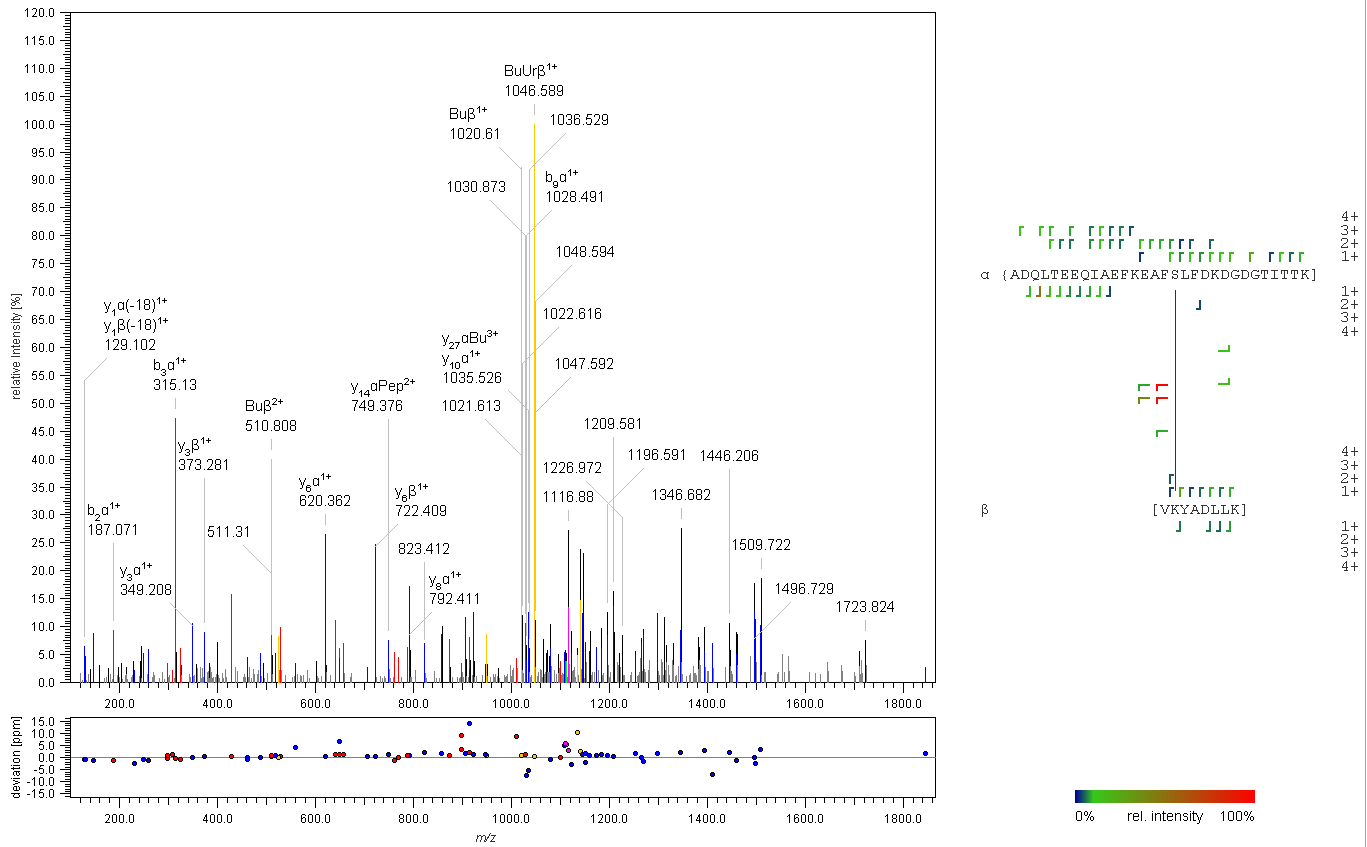

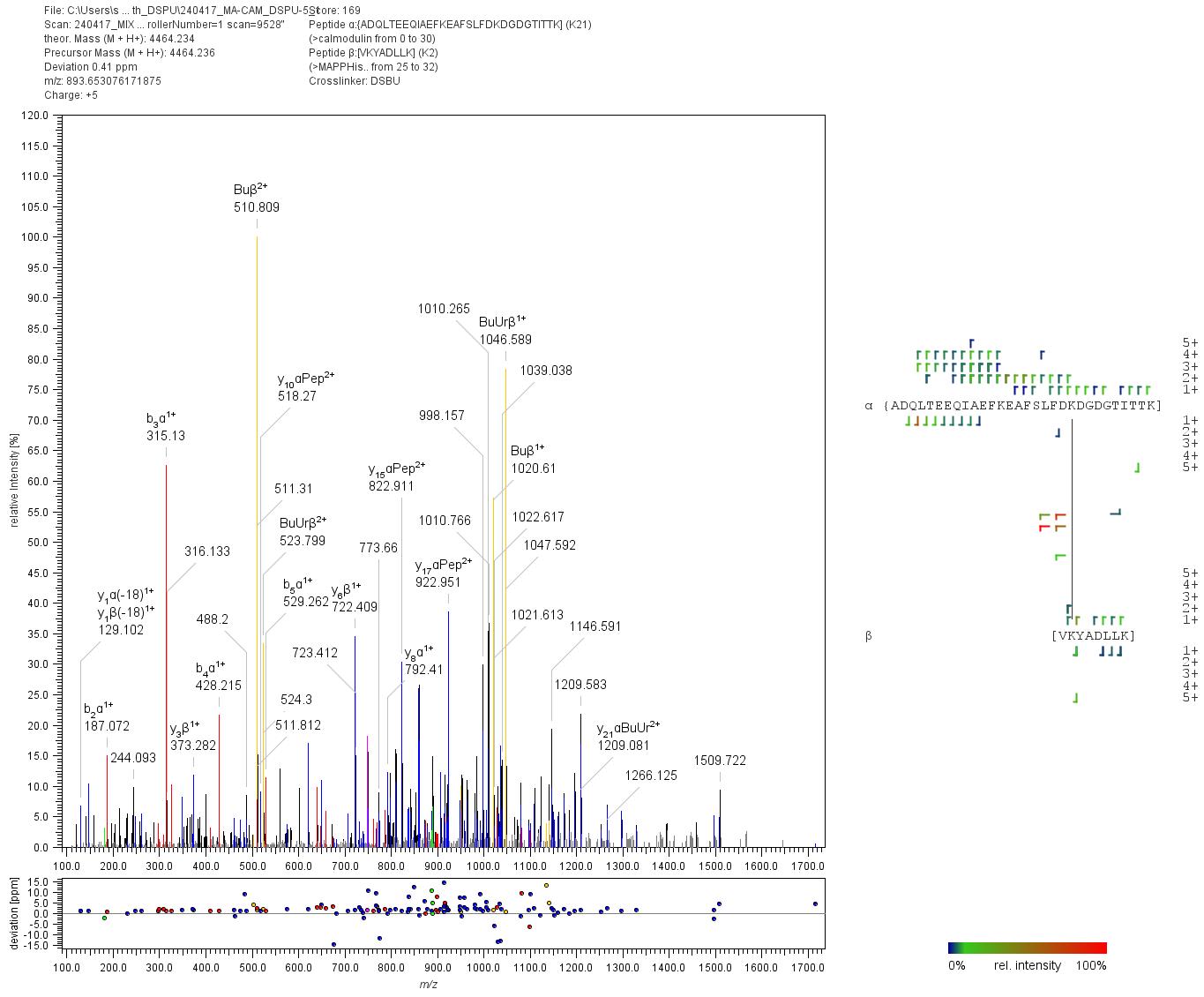


**DSPU: myrMAPP_K27-CaM_K21**

Linkage: {ADQLTEEQIAEFKEAFSLFDKDGDGTITTK] - [VKYADLLK] (K21-K27)

Peptide α: {ADQLTEEQIAEFKEAFSLFDKDGDGTITTK] (K21); (> CaM from 0 to 30)

Peptide β: [VKYADLLK] (K2); (>myrMAPP from 26 to 33)


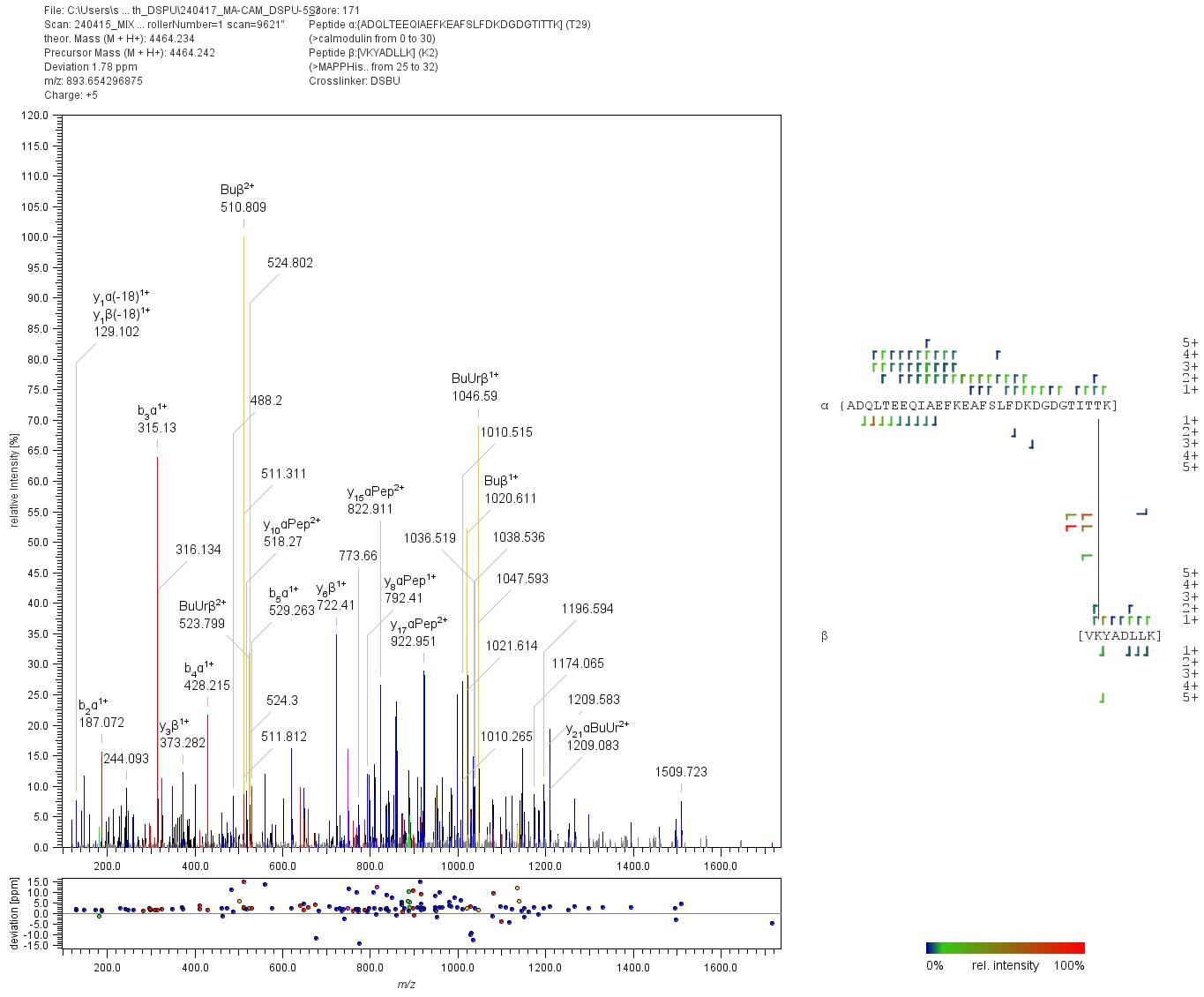


**DSPU: myrMAPP_K27-CaM_T29**

Linkage: {ADQLTEEQIAEFKEAFSLFDKDGDGTITTK] - [VKYADLLK] (T29-K27)

Peptide α: {ADQLTEEQIAEFKEAFSLFDKDGDGTITTK] (T29); (> CaM from 0 to 30)

Peptide β: [VKYADLLK] (K2); (>myrMAPP from 26 to 33)


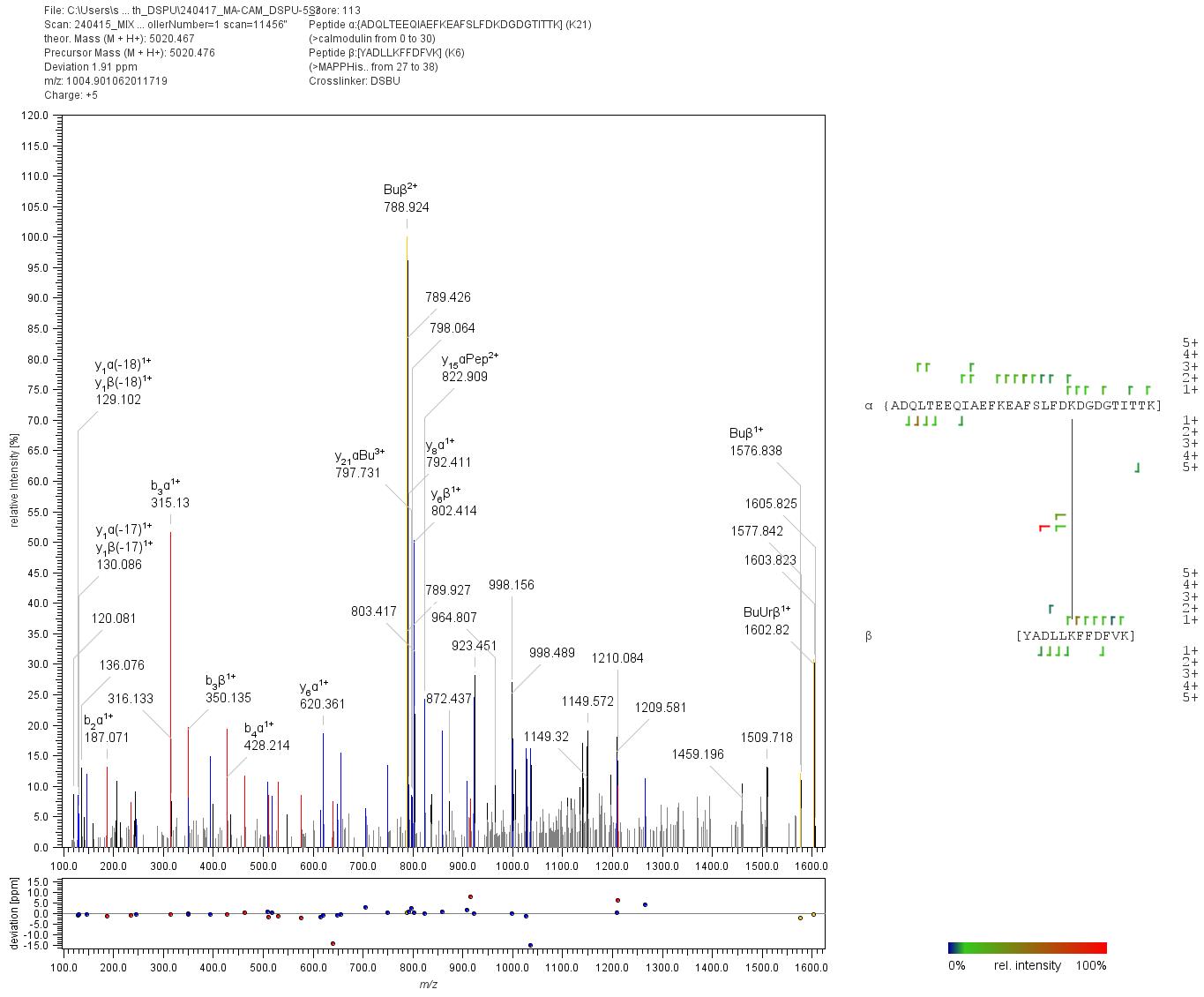


**DSPU: myrMAPP_K33-CaM_K21**

Linkage: {ADQLTEEQIAEFKEAFSLFDKDGDGTITTK] - [YADLLKFFDFVK] (K21-K33)

Peptide α: {ADQLTEEQIAEFKEAFSLFDKDGDGTITTK] (K21); (> CaM from 0 to 30)

Peptide β: [YADLLKFFDFVK] (K6); (>myrMAPP from 28 to 39)


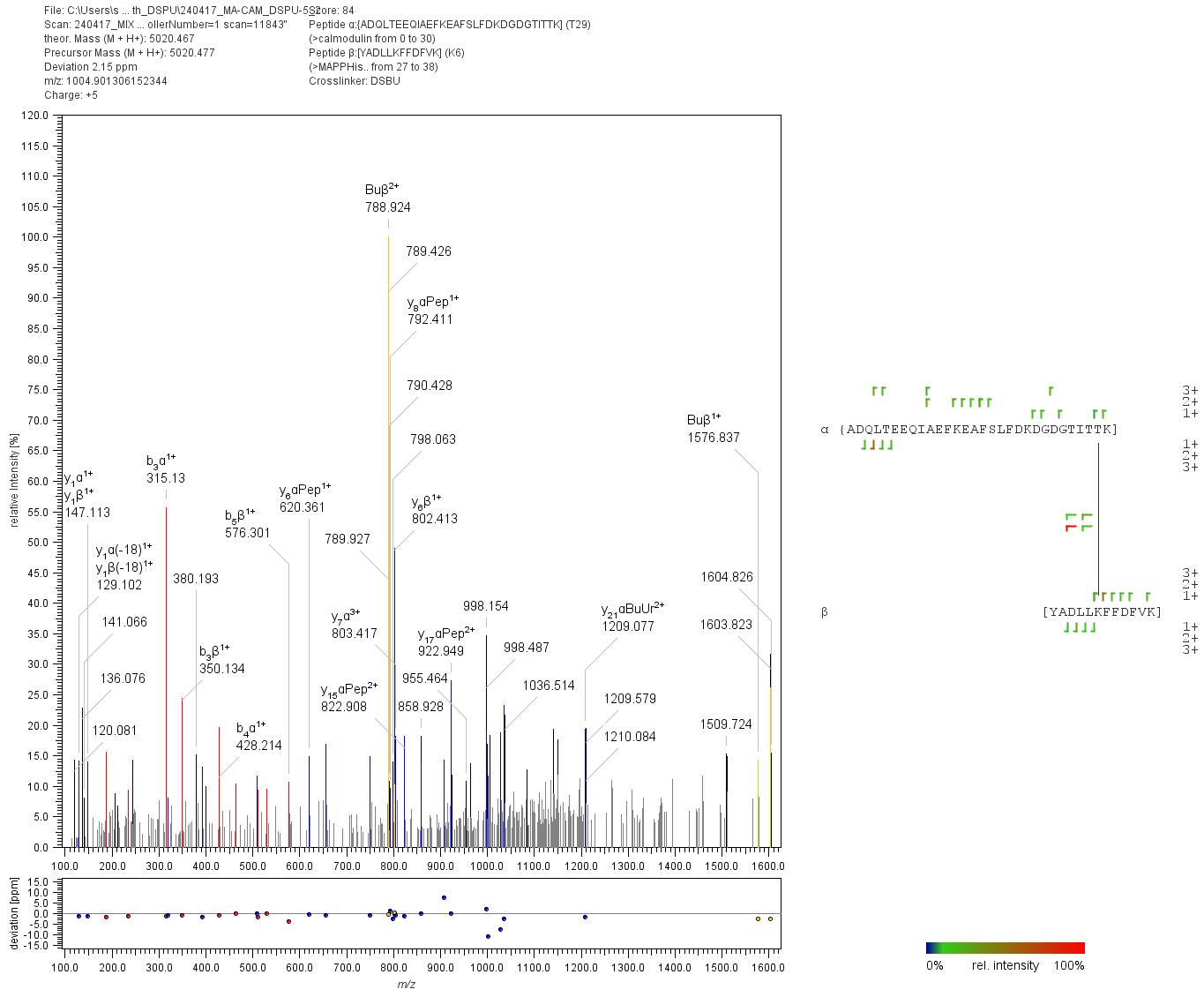


**DSPU: myrMAPP_K33-CaM_T29**

Linkage: {ADQLTEEQIAEFKEAFSLFDKDGDGTITTK] - [YADLLKFFDFVK] (T29-K33)

Peptide α: {ADQLTEEQIAEFKEAFSLFDKDGDGTITTK] (T29); (> CaM from 0 to 30)

Peptide β: [YADLLKFFDFVK] (K6); (>myrMAPP from 28 to 39)


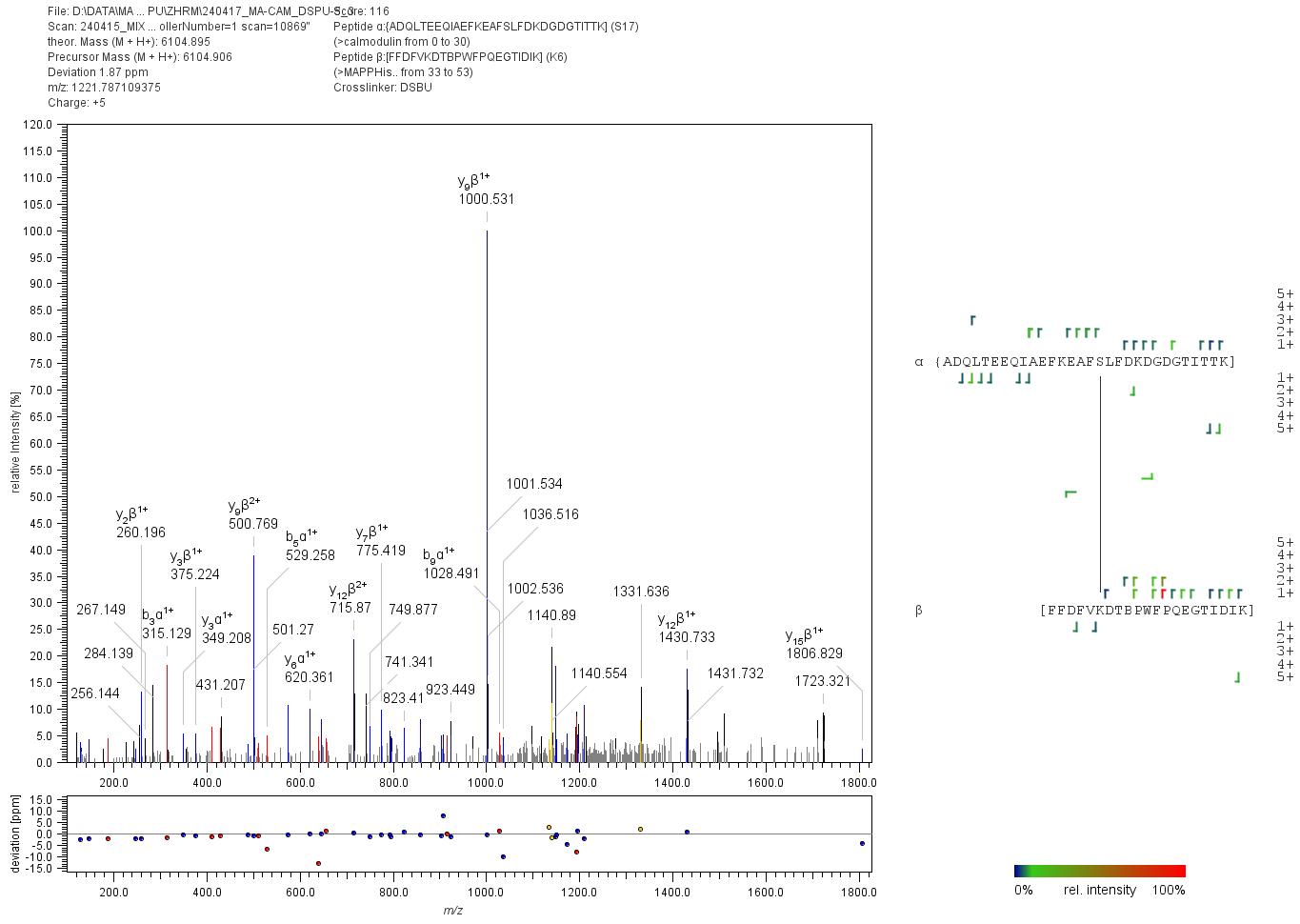


**DSPU: myrMAPP_K39-CaM_S17**

Linkage: {ADQLTEEQIAEFKEAFSLFDKDGDGTITTK] - [FFDFVKDTBPWFPQEGTIDIK] (S17-K39)

Peptide α: {ADQLTEEQIAEFKEAFSLFDKDGDGTITTK] (S17); (> CaM from 0 to 30)

Peptide β: [FFDFVKDTBPWFPQEGTIDIK] (K6); (>myrMAPP from 34 to 54)


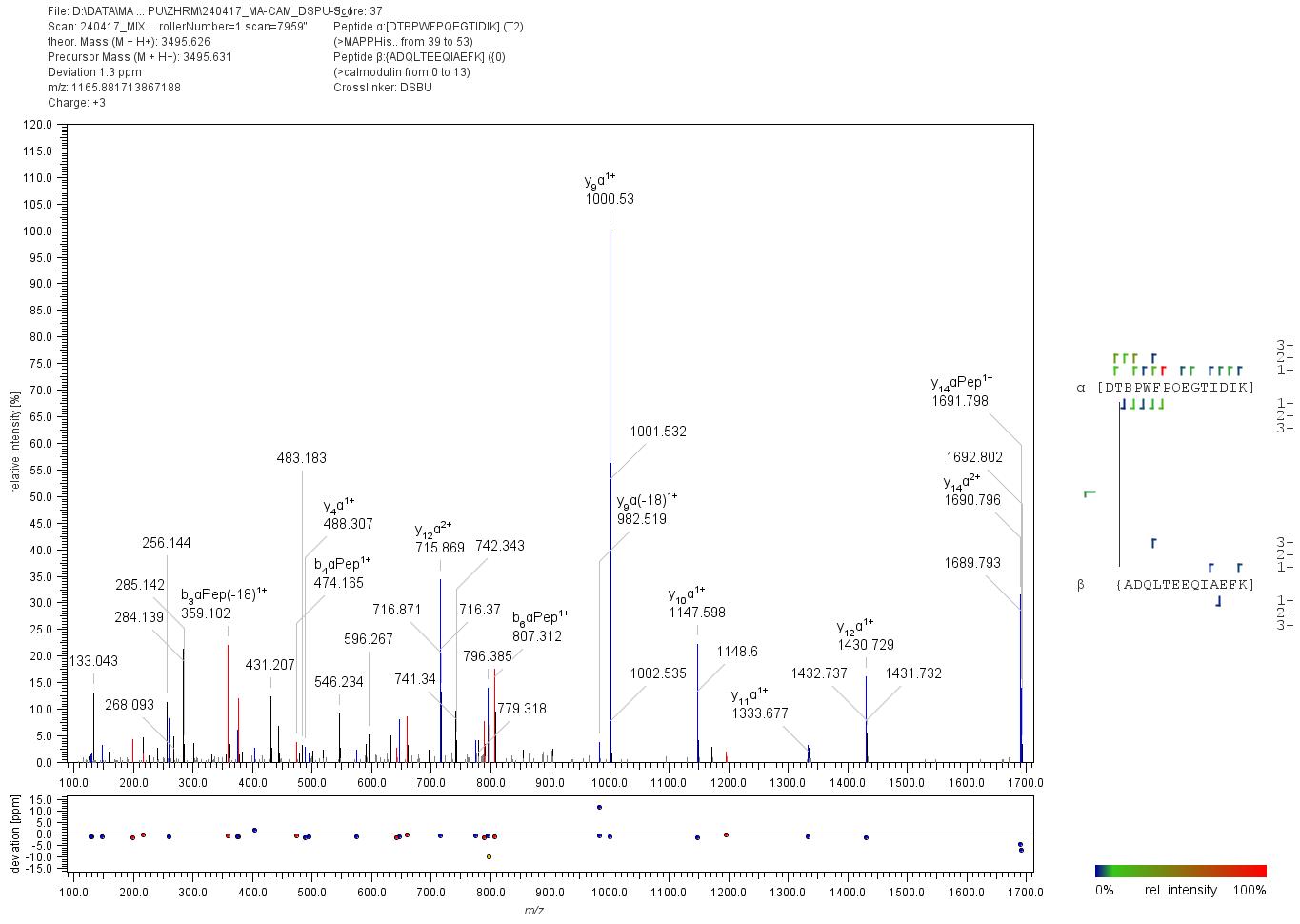


**DSPU: myrMAPP_T41-CaM_A1**

Linkage: [DTBPWFPQEGTIDIKR] - {ADQLTEEQIAEFK] (T41-0}

Peptide α: [DTBPWFPQEGTIDIKR] (T2); (>myrMAPP from 40 to 55)

Peptide β: {ADQLTEEQIAEFK] {0}; (> CaM from 0 to 13)


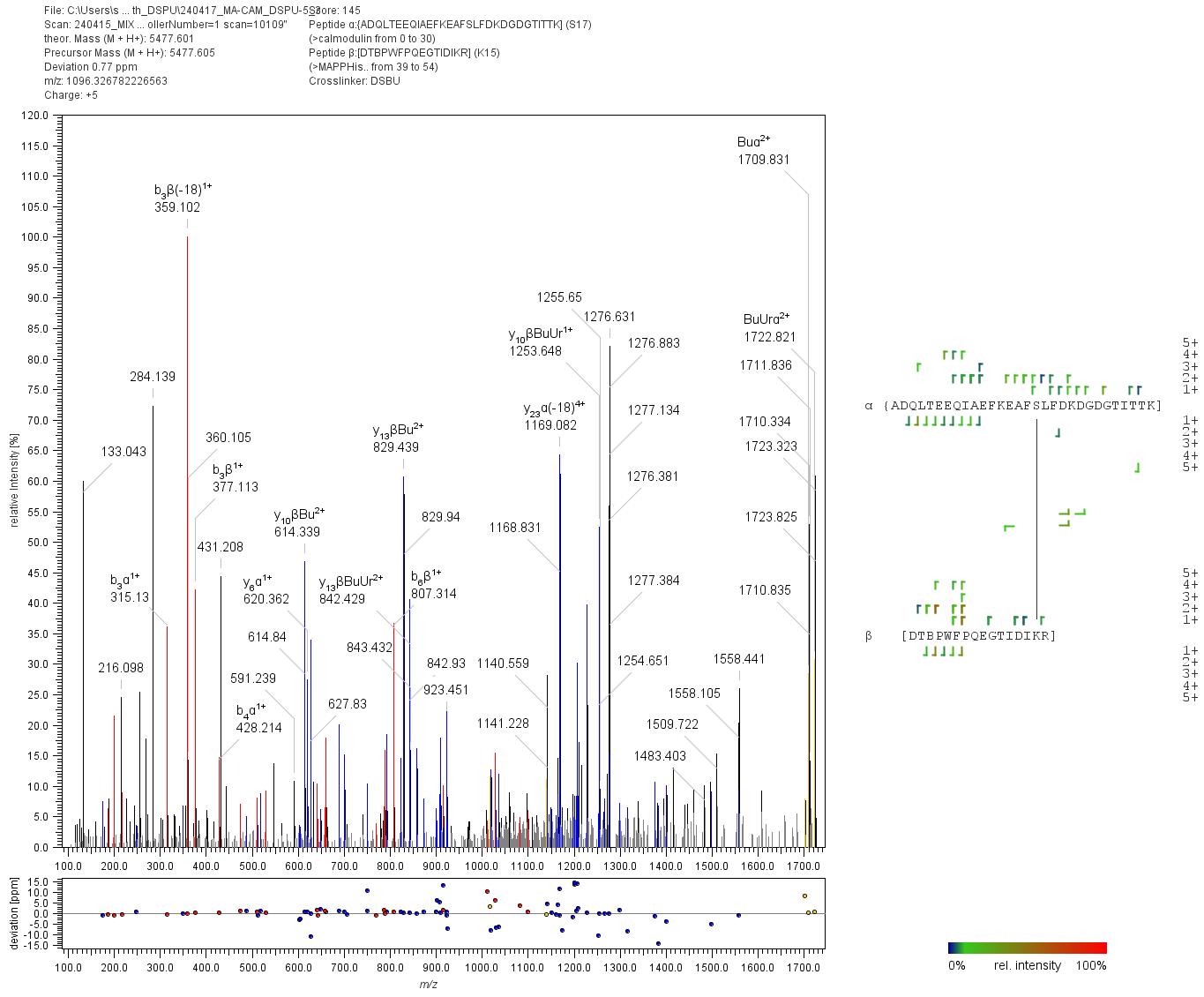


**DSPU: myrMAPP_K54-CaM_S17**

Linkage: {ADQLTEEQIAEFKEAFSLFDKDGDGTITTK] - [DTBPWFPQEGTIDIKR] (S17-K54)

Peptide α: {ADQLTEEQIAEFKEAFSLFDKDGDGTITTK] (S17); (> CaM from 0 to 30)

Peptide β: [DTBPWFPQEGTIDIKR] (K15); (>myrMAPP from 40 to 55)
